# Pore-Engineered Luminescent MOF Sensors for PFAS Recognition in Water

**DOI:** 10.1021/jacs.5c20085

**Published:** 2026-01-14

**Authors:** Zongsu Han, Kun-Yu Wang, Jiatong Huo, Wenyue Cui, Zhaoyi Liu, Yihao Yang, Rong-Ran Liang, Wei Shi, Hong-Cai Zhou

**Affiliations:** † Department of Chemistry, 14736 Texas A&M University, College Station, Texas 77843, United States; ‡ Frontiers Science Center for New Organic Matter, State Key Laboratory of Advanced Chemical Power Sources, and Department of Chemistry, College of Chemistry, 12538 Nankai University, Tianjin 300071, China

## Abstract

Per- and polyfluoroalkyl substances (PFAS) are persistent contaminants in water that pose severe threats to environmental integrity and public health. Luminescent sensing using porous materials has emerged as a highly efficient strategy for daily recognition, owing to its high efficiency, simplicity, and sensitivity. However, systematic investigations into the pore structure–function relationship that govern PFAS detection remain largely lacking, which hindered the rational design of advanced PFAS sensors. Herein, a linker installation strategy is employed to precisely engineer the pore environments of metal–organic frameworks (MOFs) in a modular manner without compromising structural integrity for PFAS recognition in water. A library of 13 PCN-700 derivatives with systematically regulated pore volumes was constructed, revealing that enhanced pore accessibility directly boosts sensing performance. Notably, the amino groups in PCN-700 significantly improve the sensing sensitivity, achieving up to 3-fold higher quenching efficiencies through strengthened host–guest interactions. Further adjustment of functional group densities uncovers a trade-off between functional group loading and pore accessibility. By disentangling the respective contributions of pore volume modulation by various functional groups, the design principles are provided for the development of robust and high-performance MOF-based luminescent sensors to address PFAS monitoring challenges in water.

## Graphical abstract

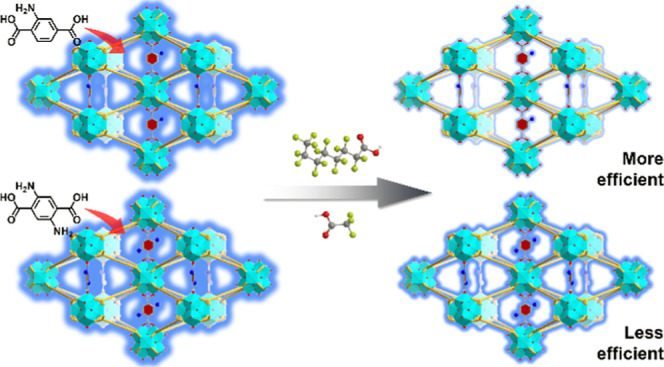

## Introduction

Per- and polyfluoroalkyl substances (PFAS) are synthetic organofluoride compounds widely used in industrial and consumer products due to their exceptional chemical stability and amphiphilic properties.^1,2^ However, the inert chemical properties of PFAS have led to their persistence, bioaccumulation, and widespread distribution in the environment.^3,4^ Long-term exposure to PFAS will arise severe health risks, including developmental toxicity, endocrine disruption, immune system impairment, and various cancers.^5–7^ The low environmental concentration and spectroscopic silence of PFAS pose grand challenges for its detection, creating an urgent demand for highly sensitive and selective detection strategies.^8,9^

Luminescent sensing has emerged as an efficient strategy for detecting various trace-level contaminants, featuring unique merits in convenient operation, on-demand applicability, and timely readout of results, which provides an affordable solution working in the complex natural environment.^10–14^ In principle, luminescent sensing requires precise matching of spectral properties, energy levels, and binding interactions between sensing material and analyte.^15–18^ However, PFAS molecules feature limited absorption in the ultraviolet–visible (UV–vis) region, prohibiting the sensing approach that rely on the competitive absorption of excitation or emission energy. While in practice, however, precise regulation and evaluation of energy levels are highly challenging due to the complex energy level variations. Therefore, systematic investigations into structurally matching between the sensing material and PFAS are scarce due to synthetic difficulty.

Metal–organic frameworks (MOFs), with highly controllable pore environments and modular structures, have been widely studied across catalysis, separation, and sensing.^19–25^ Pore engineering plays a pivotal role in this context, as it allows deliberate tailoring of the pore size, shape, and chemical environment to optimize host–guest interactions and broaden functional applications. Among various pore engineering strategies toward MOFs, linker installation has emerged as a powerful approach for precisely tuning pore environments while preserving the structural integrity of the parent framework.^26,27^ This method allows the sequential incorporation of functional linkers into predefined sites with atomic-level precision, enabling orthogonal control over pore size, pore environment, and spatial arrangement of installed molecules.^28,29^ By leveraging this pore engineering strategy, MOFs can be rationally designed to achieve optimal matching with PFAS molecules, offering a systematic approach to enhance sensing performance and unravel the underlying sensing mechanisms.

In this work, the linker installation approach was employed to construct totally 13 PCN-700 ^30^ derivatives with systematically regulated pore volumes, functional groups, and functional group densities to decouple the effects of these parameters on PFAS recognition. Through detailed analysis, we revealed that pore volume plays a dominant role in this case, while amino functionality enhances PFAS recognition due to the strong host–guest interactions. Furthermore, excessive functionalization was found to compromise pore accessibility, revealing a trade-off between binding affinity and molecular diffusion (Figure 1). This study establishes a clear structure–property correlation for PFAS sensing, offering design principles for luminescent MOF-based recognition systems and showcasing linker installation as a powerful pore engineering strategy for environmental monitoring applications.

**Figure 1:**
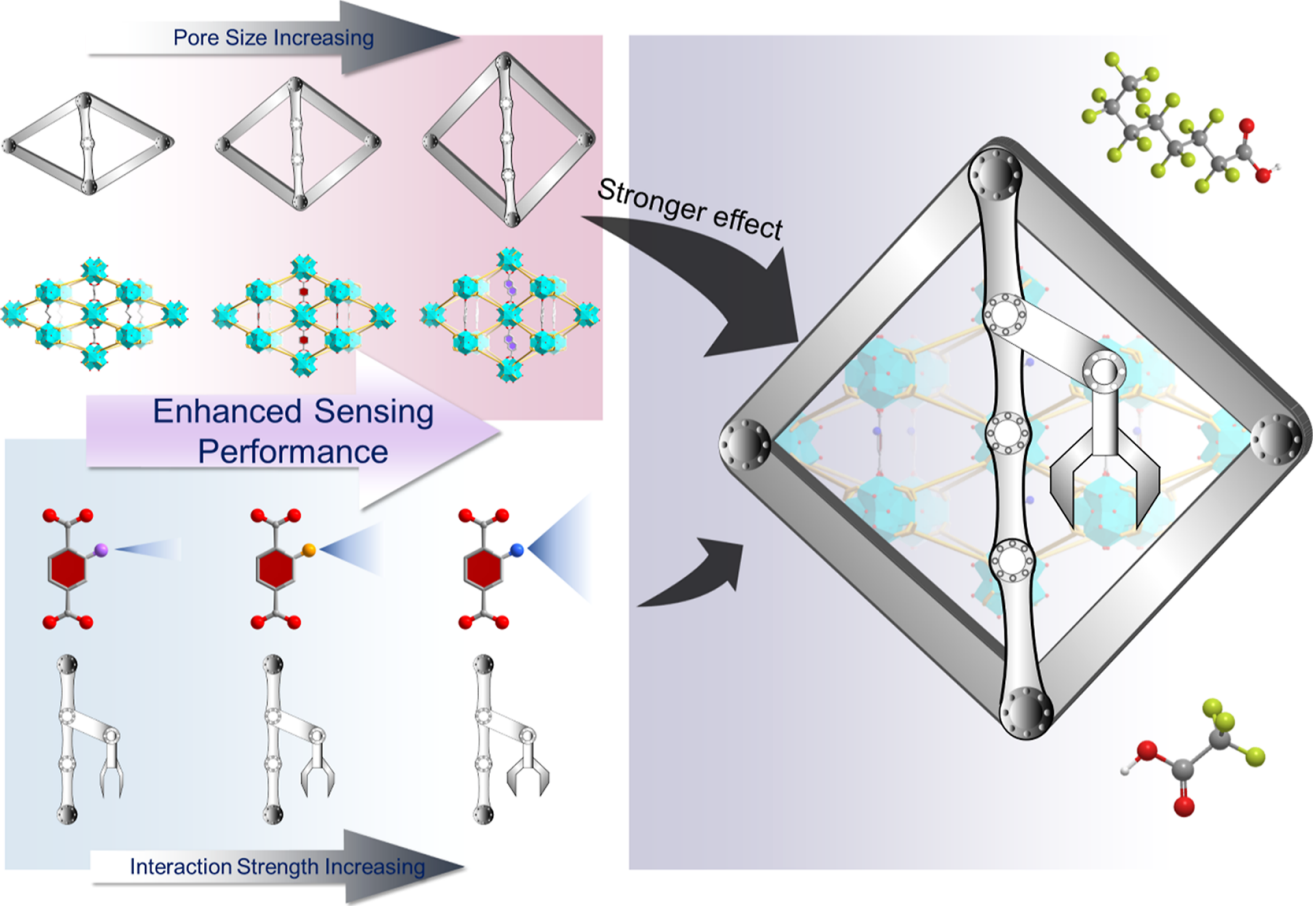
Structural factors influencing the sensing performance toward PFAS. Atom code: C, gray; O, red; H, white; F, lime.

## Results and Discussion

### Structures and Basic Characterizations

PCN-700 is assembled from Zr_6_ secondary building units connected by 2,2′-dimethylbiphenyl-4,4′-dicarboxylate (BPDC-(CH_3_)_2_^2–^) linkers, yielding a chemically robust yet conformationally flexible framework.^30,31^ The steric hindrance of the dimethyl-substituted biphenyl linkers restricts coordination, leaving each Zr_6_ node with eight coordination terminal water or hydroxyl groups. This configuration creates two distinct classes of missing-linker defects located within the pore domains (Figure 2a), thereby providing multiple accessible binding sites for postsynthetic functionalization.

**Figure 2:**
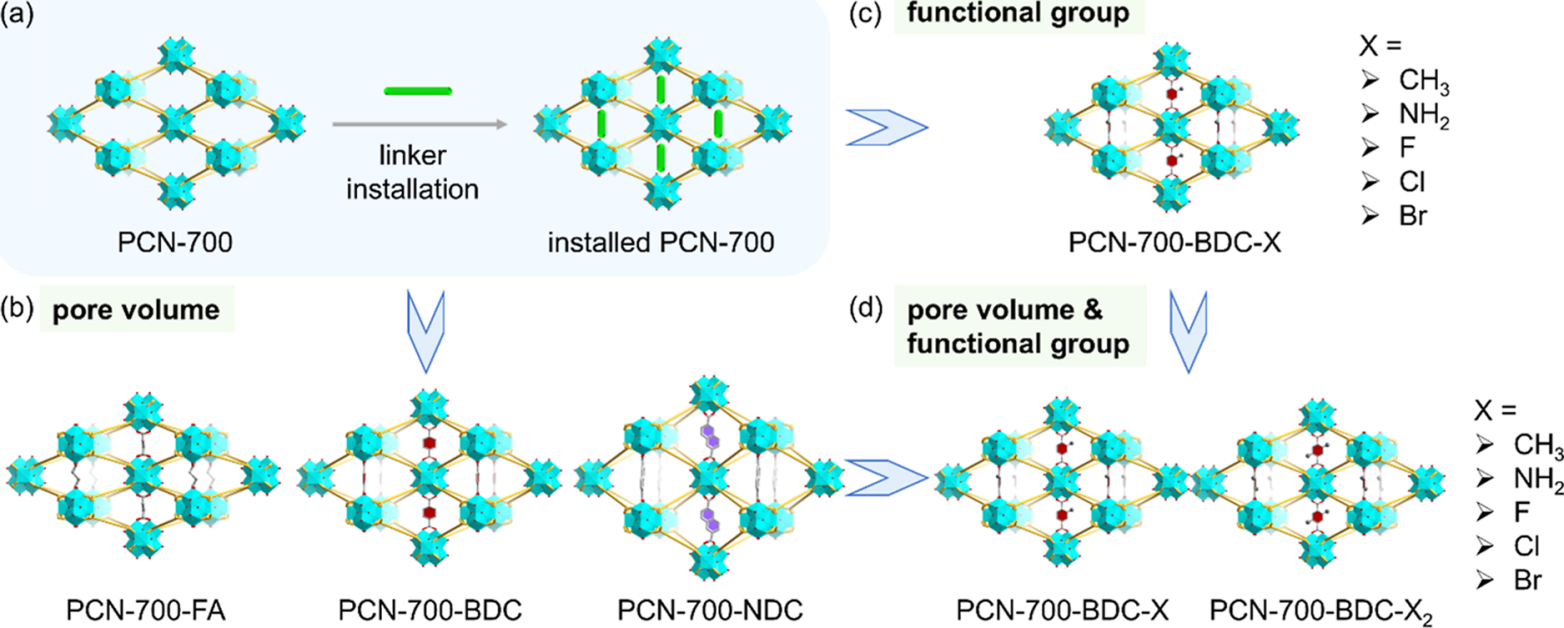
Schematic illustration of linker installation in PCN-700 (a) for systematic investigation of pore volume effects (b), functional group effects (c), and their synergistic effects (d).

Inspired by the highly adaptive nature of PCN-700, 13 linkers were installed onto the framework, respectively, to study the influence of pore volumes, functional groups, and their synergistic effects for sensing performance (Figure S1). To tune the pore volume, fumaric acid (H_2_FA), 1,4-benzenedicarboxylic acid (H_2_BDC), and 2,6-naphthalenedicarboxylic acid (H_2_NDC) were installed in PCN-700 (Figure 2b), resulting in increased cell volume (Table S1) and pore volume.^26^ To study the influence of functional groups, a series of linkers with similar molecular size and shape but different substituent groups, including 2-methyl-1,4-benzenedicarboxylic acid (H_2_BDC–CH_3_), 2-amino-1,4-benzenedicarboxylic acid (H_2_BDC–NH_2_), 2-fluoro-1,4-dicarboxylic acid (H_2_BDC–F), 2-chloro-1,4-dicarboxylic acid (H_2_BDC–Cl), and 2-bromo-1,4-dicarboxylic acid (H_2_BDC–Br), were selected and positioned in the target position in PCN-700 (Figure 2c). Furthermore, to comprehensively account for the combined effects of pore volume and functional group, the density of functional groups was employed as a comparative metric, while 2,5-dimethyl-1,4-benzenedicarboxylic acid (H_2_BDC–(CH_3_)_2_), 2,5-diamino-1,4-benzenedicarboxylic acid (H_2_BDC–(NH_2_)_2_), 2,5-difluoro-1,4-dicarboxylic acid (H_2_BDC–F_2_), 2,5-dichloro-1,4-dicarboxylic acid (H_2_BDC–Cl_2_), and 2,5-dibromo-1,4-dicarboxylic acid (H_2_BDC–Br_2_), were selected for comparison with the monosubstituted analogue (Figure 2d).

Single-crystal X-ray diffraction (SCXRD) analysis unambiguously confirmed the presence and the position of the installed linkers in PCN-700 at the molecular level (Table S1). The phase purities of these PCN-700 derivatives were verified by powder X-ray diffraction (PXRD) patterns (Figures S2–S4), and the incorporation of the linkers was further validated by ^1^H nuclear magnetic resonance (^1^H NMR) spectra of digested samples in *d*_6_-DMSO and D_2_SO_4_ (Figures S5–S20). In addition, luminescence stability measurements showed that the emission intensities of these PCN-700 derivatives remained stable in aqueous media (Figures S21–S33).

### Luminescence Sensing

Trifluoroacetic acid (TFA), an emerging short-chain PFAS, which is highly persistent and widespread in water systems due to its resistance to biodegradation, and perfluorooctanoic acid (PFOA), a long-chain PFAS, which is the mostly produced PFAS associated with bioaccumulation, toxicity, and long-term ecological impacts, were selected as representative analytes.^1–7^ All luminescence responses stabilized within 1 min, highlighting the applicability for the rapid detection of PFAS (Figures S34–S59).

The sensing abilities of these MOFs toward TFA and PFOA were tested in details (Figures S60–S85). The data were analyzed and fitted by Stern–Volmer (S–V) equation^32,33^ (Figures S86–S99). For the sensing by PCN-700-FA, PCN-700-BDC, and PCN-700-NDC featuring progressively enlarged pore volumes, the simultaneous increased quenching efficiencies (Figure 3a) and S–V quenching constants (*K*_SV_) (Figure 3b) clearly demonstrates that the enlarged pore directly facilitates more effective luminescence quenching toward both TFA and PFOA. Among the PCN-700-BDC derivatives, PCN-700-BDC-NH_2_ shows markedly superior quenching efficiencies compared to others (Figure 3), highlighting the optimal interaction and structural complementarity between the amino functionality and TFA/PFOA. Moreover, increasing the density of functional groups leads to lower quenching efficiencies toward TFA and PFOA (Figure 3b), indicating that the concomitant reduction in pore volume exerts a stronger influence on the sensing toward TFA and PFOA than the functional groups themselves. The limit of detection (LOD) of these materials was further calculated (Table S2). Interference experiments confirm that common coexisting species in aqueous media exert minor influence on the detection performance of PCN-700-BDC-NH_2_ toward PFOA and TFA (Figures S100–S103). Cycling experiments demonstrate that PCN-700-BDC-NH_2_ maintains a stable luminescence response over multiple cycles, confirming its good reversibility and reusability (Figures S104–S107). Benchmarking against representative materials shows that these MOFs exhibit sensing behavior that is broadly comparable to previously reported PFAS sensing materials (Table S3). Besides, the *K*_SV_ values of these MOFs toward PFOA are obviously higher than those toward TFA, which may be attributed to the longer chain of PFOA, enabling multivalent interactions with the framework and longer residence time within the pores, thereby accounting for the superior sensing performance.

**Figure 3:**
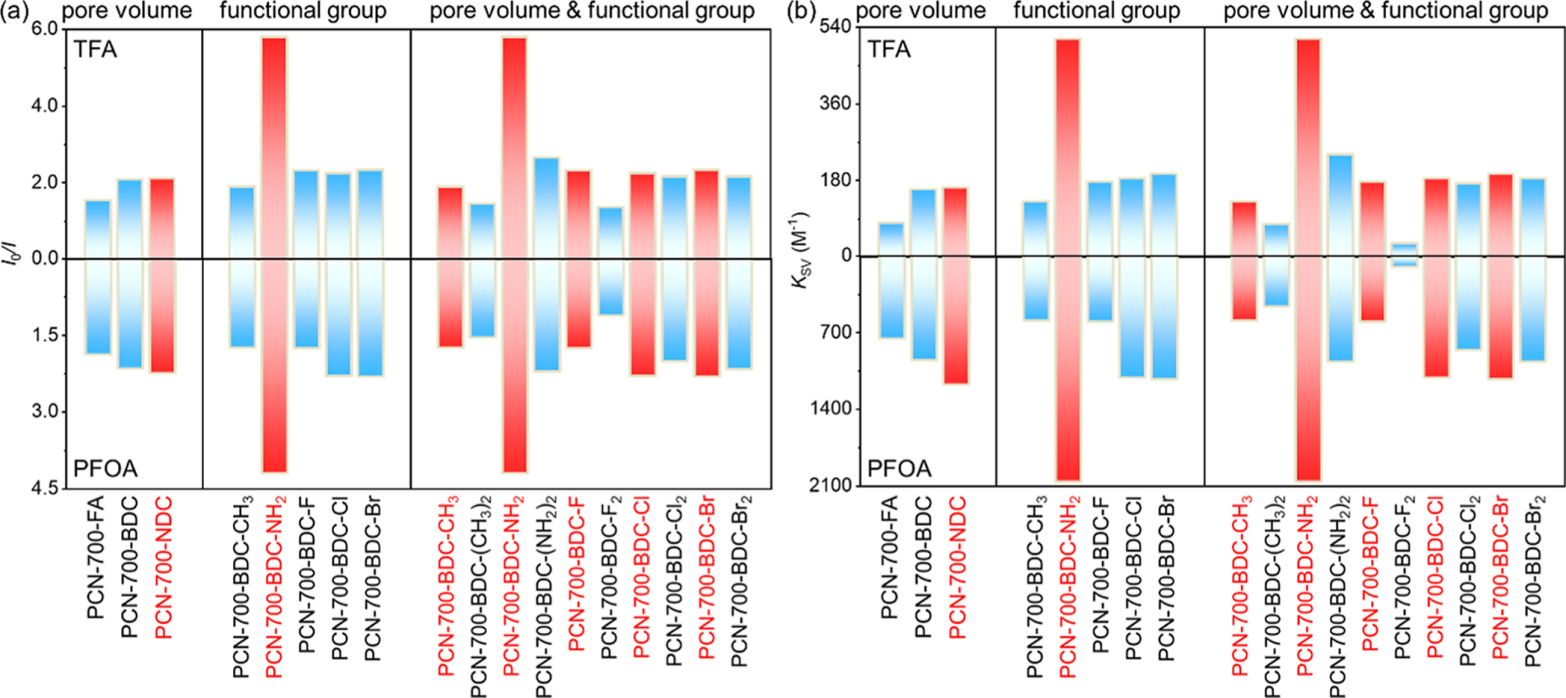
Luminescence intensity variations with 6.0 mM of TFA and 1.2 mM of PFOA (a) and quenching constants (b) of installed PCN-700 with different pore volume, functional group type, and functional group density. Blue and red pillars represent the quenching efficiencies, while red pillars stand for the samples with better performance.

### Sensing Mechanism

The sensing mechanism was studied in detail through a series of characterizations. There are no apparent changes in PXRD patterns of these installed PCN-700 before and after the sensing processes (Figures S108–S120), confirming that the main framework structures remain.^18^ According to the ultraviolet–visible (UV–vis) absorption spectra, TFA and PFOA possess extremely low light absorption capacities (Figure 4a), which induces no obvious overlap with the UV–vis absorption spectra (Figures S121–S133) or emission spectra (Figures S134–S146) of these installed PCN-700, excluding the competitive absorption and Förster resonance energy transfer (FRET) mechanisms based on the energy transfer processes.^18^ As for the electron transfer process, the highest occupied molecular orbital (HOMO) and lowest unoccupied molecular orbital (LUMO) energy levels of the linkers are calculated (Figures 4b,c and S147). The HOMO energy levels of TFA and PFOA are lower than all of the linkers, and the LUMO energy levels are higher than them, indicating the absence of photoinduced electron transfer (PET) mechanism.^18^ The lifetime changes with the additions of the analytes were measured (Figures S148 and S149), while no obvious variations were observed (Figures S150 and S151), indicating a static quenching process, caused by the binding between the MOF and analytes.

**Figure 4:**
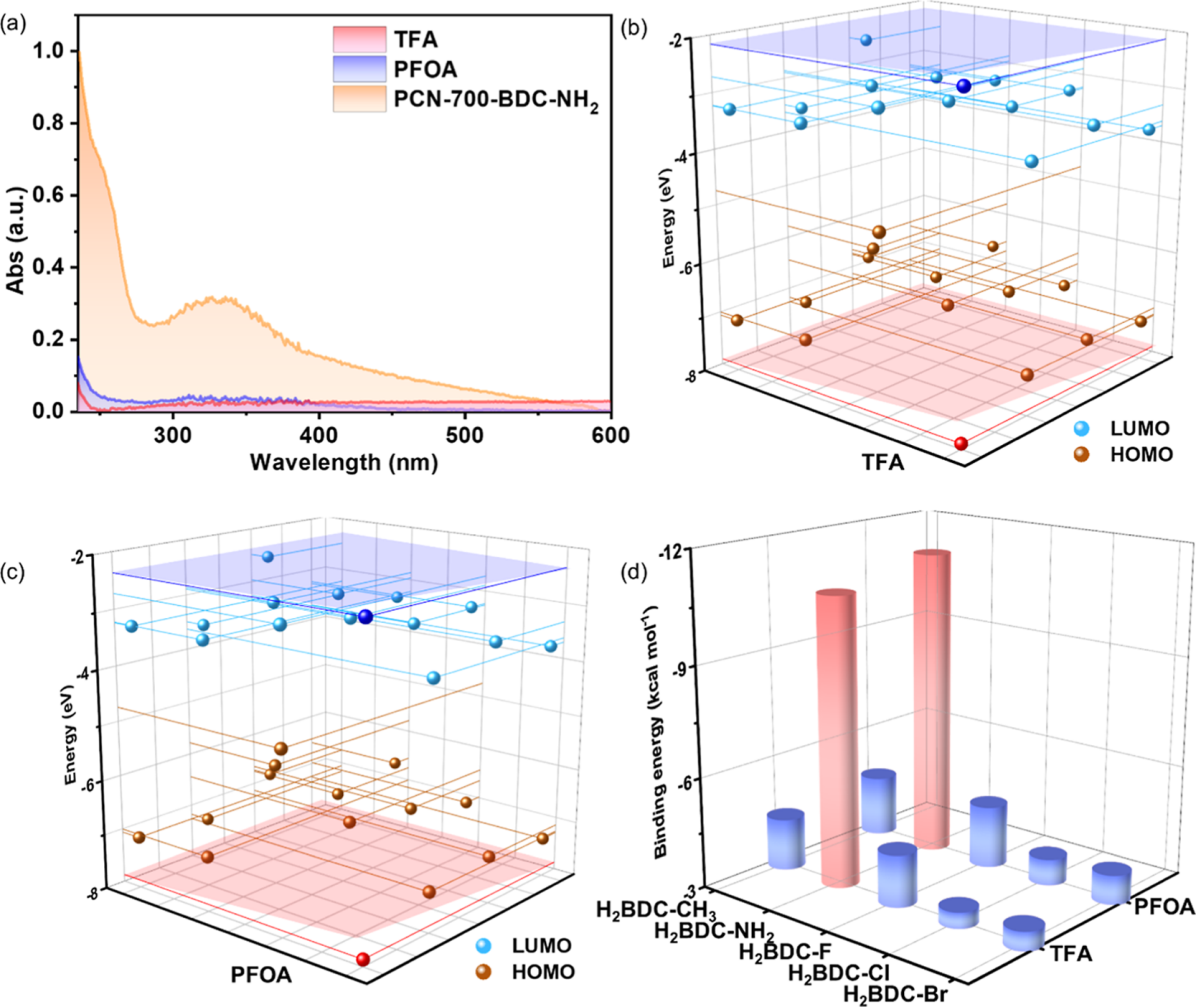
(a) Comparisons of the UV–vis spectra of TFA, PFOA, and PCN-700-BDC-NH_2_. (b) HOMO and LUMO energy levels of TFA (red and blue points) and the ligands (gold and azure points). (c) HOMO and LUMO energy levels of PFOA (red and blue points) and the ligands (gold and azure points). (d) Binding energies between the ligands and TFA/PFOA. Blue and red pillars represent the energies, while red pillars stand for the stronger binding.

To further evaluate the structural factors influencing the sensing performance, the combinations of the linkers with different functional groups with TFA and PFOA were calculated (Figures S152–S161). The results show that the amino groups possess obviously stronger interactions with TFA and PFOA than others (Figures 4d and S162), which is responsible for the best sensing performance. For MOFs with larger pore volumes, sufficient void space promotes efficient analyte diffusion and enables multiple host–guest interactions within the pores, resulting in enhanced luminescence quenching. In contrast, frameworks bearing densely installed functional groups can provide stronger local interactions with PFAS molecules, but excessive functionalization inevitably reduces pore accessibility and limits analyte transport. Consequently, the overall sensing efficiency is governed by a delicate balance between spatial accessibility and interaction strength, arising from the cooperative interplay between pore architecture and functional group chemistry. These findings highlight that the optimal luminescent sensing requires a synergistic tuning of pore architecture and functional group chemistry, rather than relying solely on either factor.

## Conclusions

In summary, this study establishes linker installation as a powerful and generalizable strategy for achieving precise control over the structure–function relationships in MOF-based PFAS sensors. Through a systematic combination of experimental characterization and theoretical analysis, the respective roles of pore volume, functional group type, and functional group density were quantitatively disentangled. The results reveal that pore accessibility primarily governs the sensing kinetics and efficiency, while the amino-functionalized linkers provide strong host–guest interactions that amplify luminescence responses toward TFA and PFOA. Together, these insights elucidate the molecular-level sensing mechanism and define a structure-guided design framework for advanced MOF-based luminescent sensors.

Beyond PFAS sensing, this work demonstrates a transferable framework for dissecting the structure–property correlations in luminescent MOF-based sensing, particularly valuable for systems where direct spectral or energy-level matching with the analyte faces challenges. The combined experimental-theoretical strategy introduced here enables the quantitative evaluation of the cooperative effects of linker chemistry and pore geometry on sensing behavior. This methodological paradigm can guide the rational design of MOF-based sensing platforms for challenging recognition processes and offers a systematic route toward developing intelligent framework materials with tunable photophysical responses and programmable host–guest interactions.

## Supplementary Material



## Notes

The experimental details, basic characterizations of the relative MOFs, luminescence sensing, sensing mechanism and relative tables for these MOFs toward analytes (PDF)

## References

[1] Ameduri B., Hori H. (2023). Recycling and the end of life assessment of fluoropolymers: recent
developments, challenges and future trends. Chem. Soc. Rev..

[2] Lim X. (2023). Can the world
leave ‘forever chemicals’ behind?. Nature.

[3] Zhang C., Yan K., Fu C., Peng H., Hawker C. J., Whittaker A. K. (2022). Biological
utility of fluorinated compounds: from materials design to molecular
imaging, therapeutics and environmental remediation. Chem. Rev..

[4] Liang R.-R., Fu Y., Han Z., Yang Y., Bakhmutov V. I., Liu Z., Rushlow J., Zhou H.-C. (2024). A robust pyrazolate metal-organic
framework for integrated perfluorooctanoic acid concentration and
degradation. Nat. Water.

[5] Taibl K. R., Dunlop A. L., Barr D. B., Li Y.-Y., Eick S. M., Kannan K., Ryan P. B., Schroder M., Rushing B., Fennell T., Chang C.-J., Tan Y., Marsit C. J., Jones D. P., Liang D. (2023). Newborn metabolomic
signatures of
maternal per- and polyfluoroalkyl substance exposure and reduced length
of gestation. Nat. Commun..

[6] Manning I. M., Guan Pin Chew N., Macdonald H. P., Miller K. E., Strynar M. J., Coronell O., Leibfarth F. A. (2022). Hydrolytically stable ionic fluorogels
for high-performance remediation of per- and polyfluoroalkyl substances
(PFAS) from natural water. Angew. Chem., Int.
Ed..

[7] Sheldon D. J., Parr J. M., Crimmin M. R. (2023). Room temperature
defluorination of
poly­(tetrafluoroethylene) by a magnesium reagent. J. Am. Chem. Soc..

[8] Concellon A., Castro-Esteban J., Swager T. M. (2023). Ultratrace PFAS detection using amplifying
fluorescent polymers. J. Am. Chem. Soc..

[9] Ji W., Xiao L., Ling Y., Ching C., Matsumoto M., Bisbey R. P., Helbling D. E., Dichtel W. R. (2018). Removal of GenX
and perfluorinated alkyl substances from water by amine-functionalized
covalent organic frameworks. J. Am. Chem. Soc..

[10] Yang M., Guo X., Mou F., Guan J. (2023). Lighting up
micro-/nanorobots with
fluorescence. Chem. Rev..

[11] Gutiérrez M., Zhang Y., Tan J. C. (2022). Confinement of luminescent
guests
in metal-organic frameworks: understanding pathways from synthesis
and multimodal characterization to potential applications of LG@MOF
systems. Chem. Rev..

[12] Jang W., Yoo H., Shin D., Noh S., Kim J. Y. (2025). Colorimetric identification
of colorless acid vapors using a metal-organic framework-based sensor. Nat. Commun..

[13] Feng X., Wang X., Redshaw C., Tang B. Z. (2023). Aggregation behaviour
of pyrene-based luminescent materials, from molecular design and optical
properties to application. Chem. Soc. Rev..

[14] Kreno L. E., Leong K., Farha O. K., Allendorf M., Van Duyne R. P., Hupp J. T. (2012). Metal-organic framework materials
as chemical sensors. Chem. Rev..

[15] Han Z., Wang K., Zhou H.-C., Cheng P., Shi W. (2023). Preparation
and quantitative analysis of multicenter luminescence materials for
sensing function. Nat. Protoc..

[16] Sun C.-Y., Wang X.-L., Zhang X., Qin C., Li P., Su Z.-M., Zhu D.-X., Shan G.-G., Shao K.-Z., Wu H., Li J. (2013). Efficient and tunable
white-light emission of metal-organic
frameworks by iridium-complex encapsulation. Nat. Commun..

[17] Perego J., Villa I., Pedrini A., Padovani E. C., Crapanzano R., Vedda A., Dujardin C., Bezuidenhout C. X., Bracco S., Sozzani P. E., Comotti A., Gironi L., Beretta M., Salomoni M., Kratochwil N., Gundacker S., Auffray E., Meinardi F., Monguzzi A. (2021). Composite
fast scintillators based on high-*Z* fluorescent metal-organic
framework nanocrystals. Nat. Photonics.

[18] Zhao Y., Zeng H., Zhu X.-W., Lu W., Li D. (2021). Metal-organic
frameworks as photoluminescent biosensing platforms: mechanisms and
applications. Chem. Soc. Rev..

[19] Batten S. R., Champness N. R., Chen X. M., Garcia-Martinez J., Kitagawa S., Ohrstrom L., O’Keeffe M., Paik Suh M., Reedijk J. (2013). Terminology of metal-organic frameworks
and coordination polymers (IUPAC Recommendations 2013). Pure Appl. Chem..

[20] Chakraborty G., Park I.-H., Medishetty R., Vittal J. J. (2021). Two-dimensional
metal-organic framework materials: synthesis, structures, properties
and applications. Chem. Rev..

[21] Cai G., Yan P., Zhang L., Zhou H.-C., Jiang H.-L. (2021). Metal-organic
framework-based
hierarchically porous materials: synthesis and applications. Chem. Rev..

[22] Xu L.-H., Li S.-H., Mao H., Li Y., Zhang A.-S., Wang S., Liu W.-M., Lv J., Wang T., Cai W.-W., Sang L., Xie W.-W., Pei C., Li Z.-Z., Feng Y.-N., Zhao Z.-P. (2022). Highly flexible
and superhydrophobic MOF nanosheet membrane for ultrafast alcohol-water
separation. Science.

[23] Datta S. J., Mayoral A., Murthy Srivatsa Bettahalli N., Bhatt P. M., Karunakaran M., Carja I. D., Fan D., Graziane
M Mileo P., Semino R., Maurin G., Terasaki O., Eddaoudi M. (2022). Rational design of mixed-matrix metal-organic framework
membranes for molecular separations. Science.

[24] Zhou S., Shekhah O., Ramirez A., Lyu P., Abou-Hamad E., Jia J., Li J., Bhatt P. M., Huang Z., Jiang H., Jin T., Maurin G., Gascon J., Eddaoudi M. (2022). Asymmetric pore windows
in MOF membranes for natural gas valorization. Nature.

[25] Zhang W., Jiang H., Liu Y., Hu Y., Palakkal A. S., Zhou Y., Sun M., Du E., Gong W., Zhang Q., Jiang J., Dong J., Liu Y., Li D., Zhu Y., Cui Y., Duan X. (2025). Metal-halide
porous
framework superlattices. Nature.

[26] Yuan S., Chen Y.-P., Qin J.-S., Lu W., Zou L., Zhang Q., Wang X., Sun X., Zhou H.-C. (2016). Linker
installation: engineering pore environment with precisely placed functionalities
in zirconium MOFs. J. Am. Chem. Soc..

[27] Pang J., Yuan S., Qin J.-S., Lollar C. T., Huang N., Li J., Wang Q., Wu M., Yuan D., Hong M., Zhou H.-C. (2019). Tuning the ionicity
of stable metal-organic frameworks
through ionic linker installation. J. Am. Chem.
Soc..

[28] Han Z., Wang K.-Y., Liang R., Yang Y., Huo J., Zhou H.-C. (2025). Linker installation
in a metal-organic framework for
enhanced quantitative redox species recognition. Angew. Chem., Int. Ed..

[29] Han Z., Sun T., Liang R.-R., Guo Y., Yang Y., Wang M., Mao Y., Taylor P. R., Shi W., Wang K.-Y., Zhou H.-C. (2024). Chiral
linker installation in a metal-organic framework for enantioselective
luminescent sensing. J. Am. Chem. Soc..

[30] Yuan S., Lu W., Chen Y.-P., Zhang Q., Liu T.-F., Feng D., Wang X., Qin J., Zhou H.-C. (2015). Sequential linker
installation: precise placement of functional groups in multivariate
metal-organic frameworks. J. Am. Chem. Soc..

[31] Qin J.-S., Yuan S., Alsalme A., Zhou H.-C. (2017). Flexible zirconium
MOF as the crystalline sponge for coordinative alignment of dicarboxylates. ACS Appl. Mater. Interfaces.

[32] Wei W., Lu R., Tang S., Liu X. (2015). Highly cross-linked fluorescent poly
(cyclotriphosphazene-co-curcumin) microspheres for the selective detection
of picric acid in solution phase. J. Mater.
Chem. A.

[33] Dinda D., Gupta A., Shaw B. K., Sadhu S., Saha S. K. (2014). Highly
selective detection of trinitrophenol by luminescent functionalized
reduced graphene oxide through FRET mechanism. ACS Appl. Mater. Interfaces.

