# Supplementary material for: Pore-Engineered Luminescent MOF Sensors for PFAS Recognition in Water
Source: J Am Chem Soc. 2026 Jan 14;148(3):3697–702. doi: 10.1021/jacs.5c20085 (PMC12856896; doi:10.1021/jacs.5c20085)
Supplement: Supplementary file 1 [file ja5c20085_si_001.pdf]

Supporting Information

Pore-Engineered Luminescent MOF Sensors for PFAS Recognition in Water

Zongsu Han,† Kun-Yu Wang,† Jiatong Huo,† Wenyue Cui,‡ Zhaoyi Liu,† Yihao Yang,† Rong-Ran Liang,† Wei Shi,‡,* and Hong-Cai Zhou†,*

†Department of Chemistry, Texas A&M University, College Station, Texas 77843, United States. E-mail: zhou@chem.tamu.edu.

‡Frontiers Science Center for New Organic Matter, State Key Laboratory of Advanced Chemical Power Sources, and Department of Chemistry, College of Chemistry, Nankai University, Tianjin 300071, China. E-mail: shiwei@nankai.edu.cn.

Contents

Experimental section 2

Structures and Basic Characterizations 4

Luminescence Sensing 19

Sensing Mechanisum 47

Tables 75

References 83

Experimental section

Materials and Methods

All reagents were commercially available and used without further purification. Liquid ^{1}H NMR spectra were recorded on a Bruker Avance NEO 400 NMR spectrometer. SCXRD patterns were collected by a Bruker-Axs Venture Ius Cmos Kappa X-ray Apex2 diffractometer with Cu-Kα radiation and a Bruker-Axs Quest Ius Three-Circle X-ray Apex2 diffractometer with Mo-Kα radiation. PXRD measurements were performed using a Bruker Powder-ECO X-ray diffractometer with Cu-Kα radiation. Luminescence spectra were recorded on a Horiba Fluoromax-4 fluorescence spectrophotometer. UV-vis absorption spectra were measured by a Shimadzu UV-2450 absorption spectrometer. For NMR tests, 3 mg linkers were dissolved by 0.5 mL d_6-DMSO, while 3 mg MOFs were dissolved by 0.5 mL d_6-DMSO and 5 μL D_2SO_4.

Synthesis

PCN-700 was synthesized according to the literature.[1] For linker installations in PCN-700, 10 mg PCN-700 was treated with a solution of 20 mg linkers in 10 mL DMF at 80 ºC for several days to ensure that the amount of incorporated linkers was approximately the same among different samples. Then the crystals were collected by filtration, washed with fresh DMF, and soaked in fresh DMF overnight.

Luminescence experiments

All samples were grinded into fine powder before use. The samples for luminescence experiments were dispersed in water by ultrasound for 10 minutes to form a clear suspension with a concentration of 0.3 mg mL^{-1}. Each line was tested after 10 seconds ultrasound.

Calculation methods

All calculations were performed using ORCA software package (version 5.0.4).[2,3] The geometry optimization for all molecules were carried out at r^2SCAN-3c/def2-mTZVVP/def2-mTZVVP/J[4] level. All calculations were corrected utilizing the geometrical counterpoise correction gCP[5] and the atom-pairwise dispersion correction based on tight binding partial charges (D4).[6,7] Single-point energy refinements were performed using the PWPB95-D3(BJ) functional with the def2-TZVPP basis set and the def2/J and def2-TZVPP/C auxiliary basis sets. The RIJCOSX approximation was used to accelerate hybrid calculations. The final binding energies were corrected for the basis set superposition error with the counterpoise method. To visualize the nature of the intermolecular interactions, an analysis based on the Independent Gradient Model by Hirshfeld partitioning[8] was conducted using the Multiwfn program,[9,10] and all figures were rendered with Visual Molecular Dynamics.[11] No additional structural constraints were applied in the optimization process.

Structures and Basic Characterizations

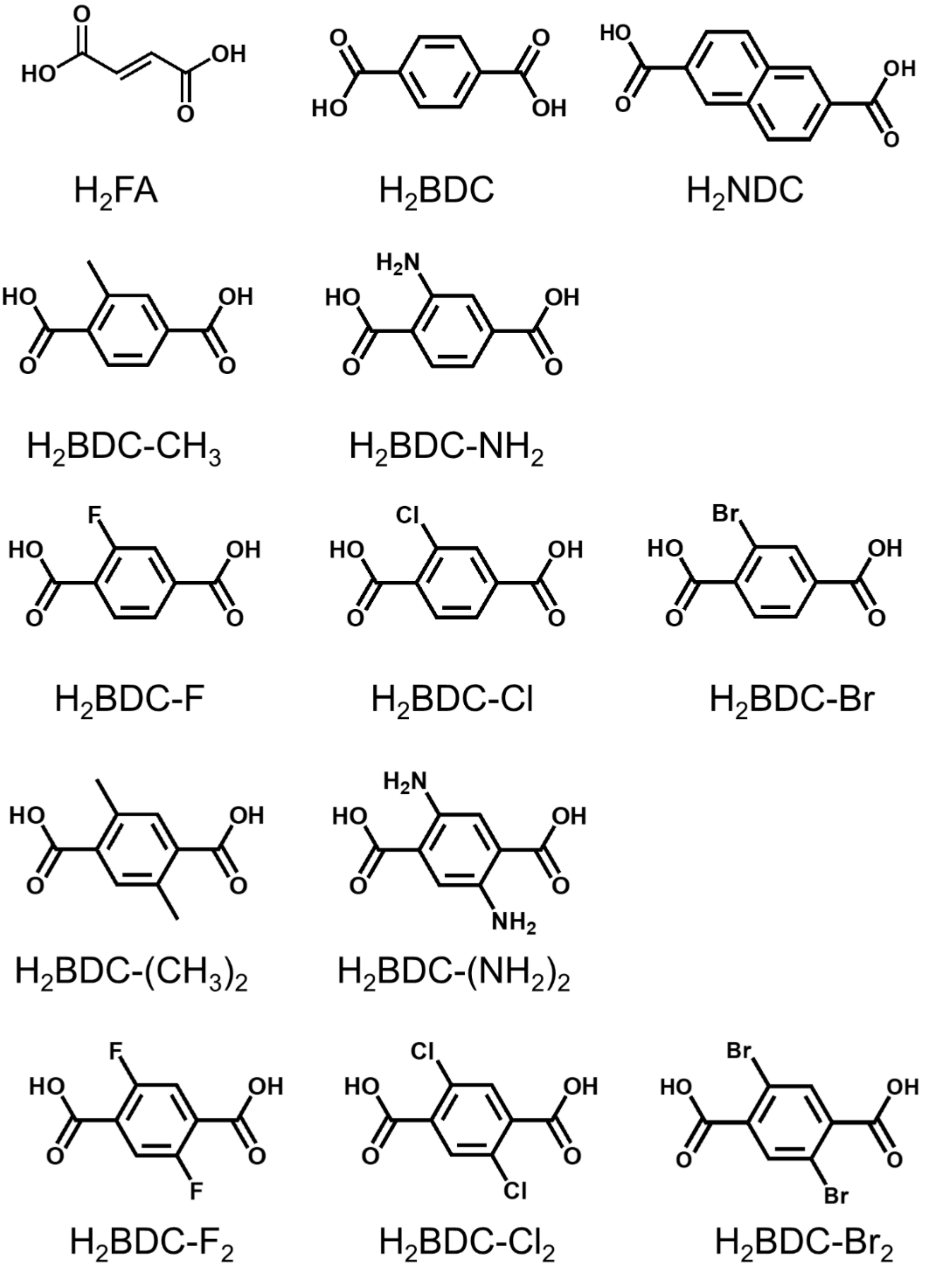

Figure S1. Linkers installed into PCN-700 in this work.

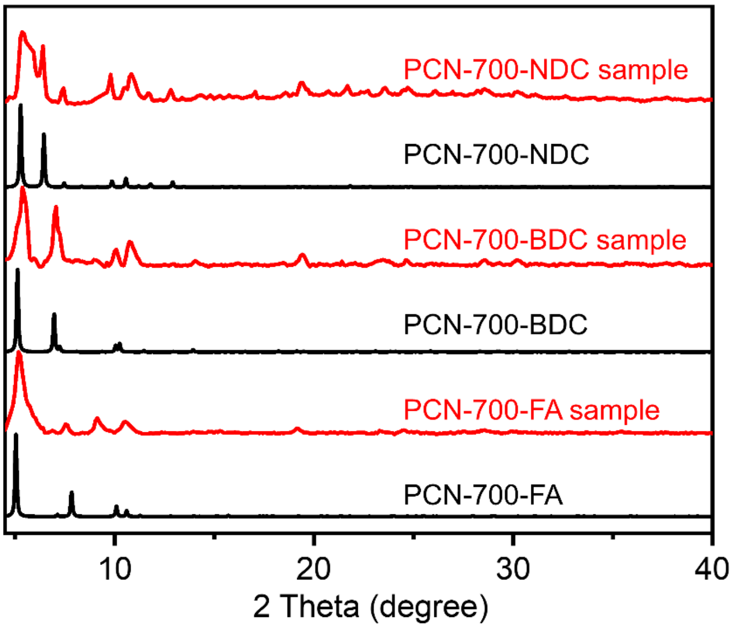

Figure S2. PXRD patterns of simulated and as-synthesized PCN-700-FA, PCN-700-BDC and PCN-700-NDC.

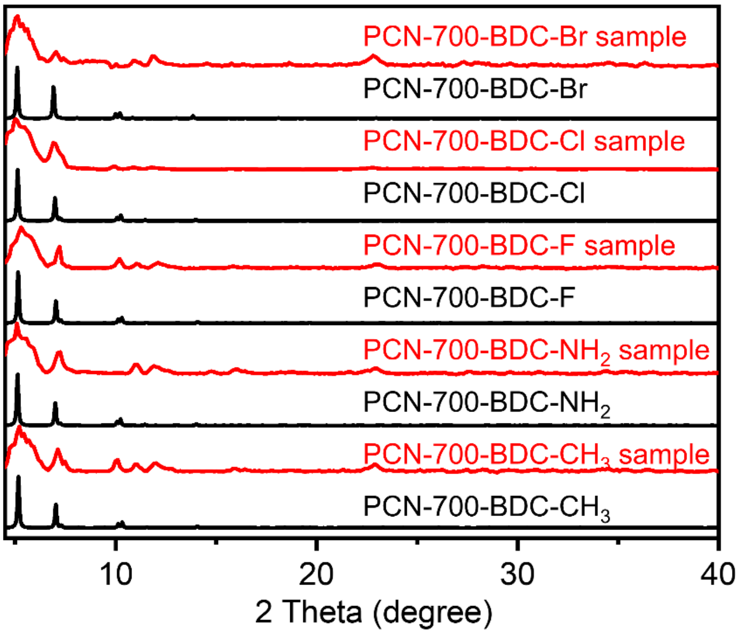

Figure S3. PXRD patterns of simulated and as-synthesized PCN-700-BDC-CH_3, PCN-700-BDC-NH_2, PCN-700-BDC-F, PCN-700-BDC-Cl and PCN-700-BDC-Br.

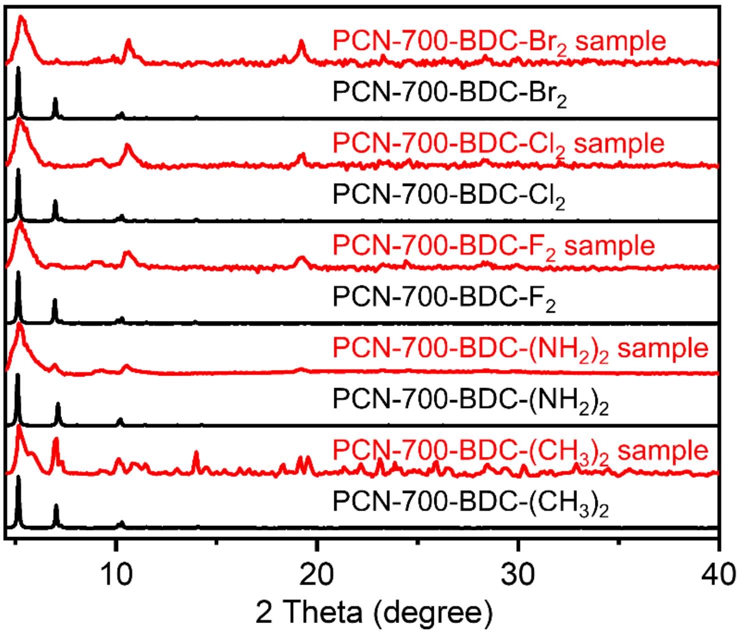

Figure S4. PXRD patterns of simulated and as-synthesized PCN-700-BDC-$(CH_3)_2$, PCN-700-BDC-$(NH_2)_2$, PCN-700-BDC-$(OH)_2$, PCN-700-BDC-F_2, PCN-700-BDC-Cl_2 and PCN-700-BDC-Br_2.

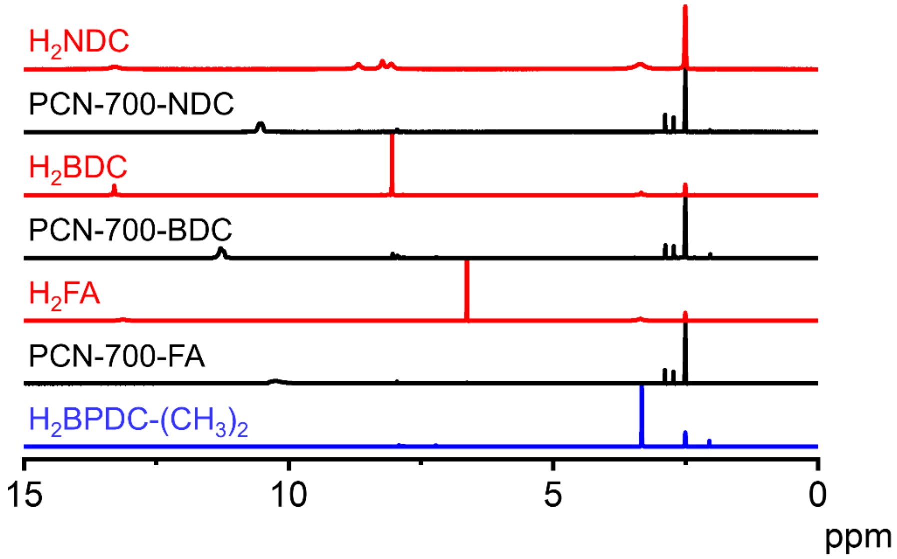

Figure S5. Liquid ^{1}H NMR spectra of H_2BPDC-$(CH_3)_2$, PCN-700-FA, H_2FA, PCN-700-BDC, H_2BDC, PCN-700-NDC and H_2NDC.

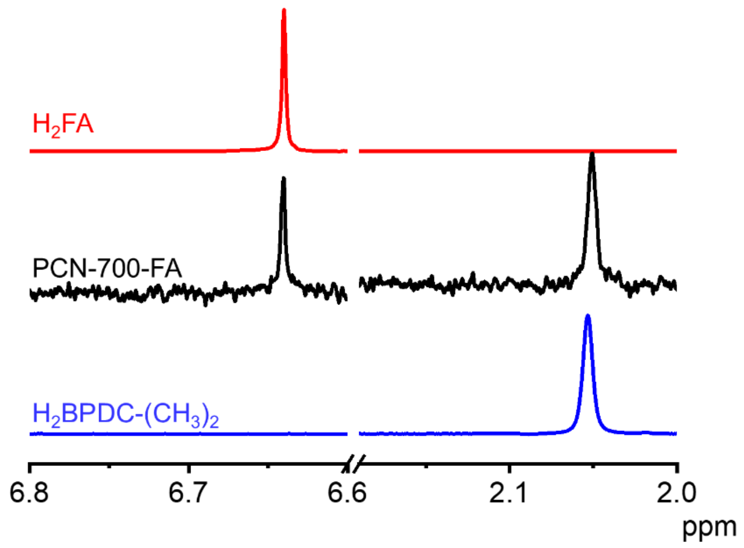

Figure S6. Liquid ^{1}H NMR spectra of $H_2BPDC-(CH_3)_2$, PCN-700-FA and H_2FA.

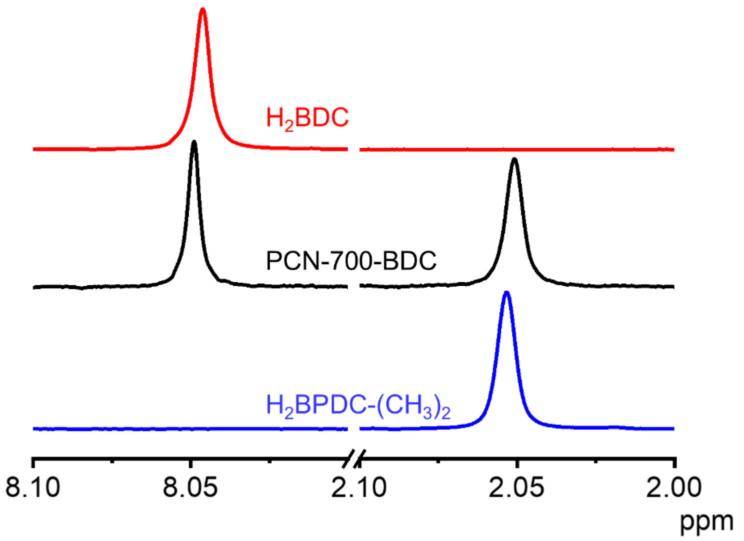

Figure S7. Liquid ^{1}H NMR spectra of $H_2BPDC-(CH_3)_2$, PCN-700-BDC and H_2BDC.

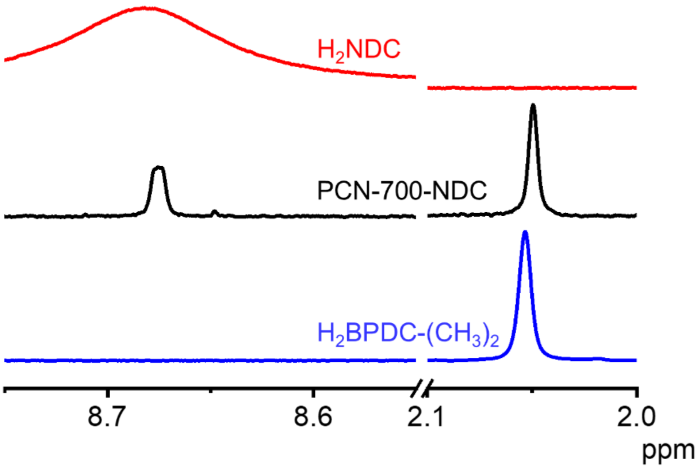

Figure S8. Liquid ^{1}H NMR spectra of $H_2BPDC-(CH_3)_2$, PCN-700-NDC and H_2NDC.

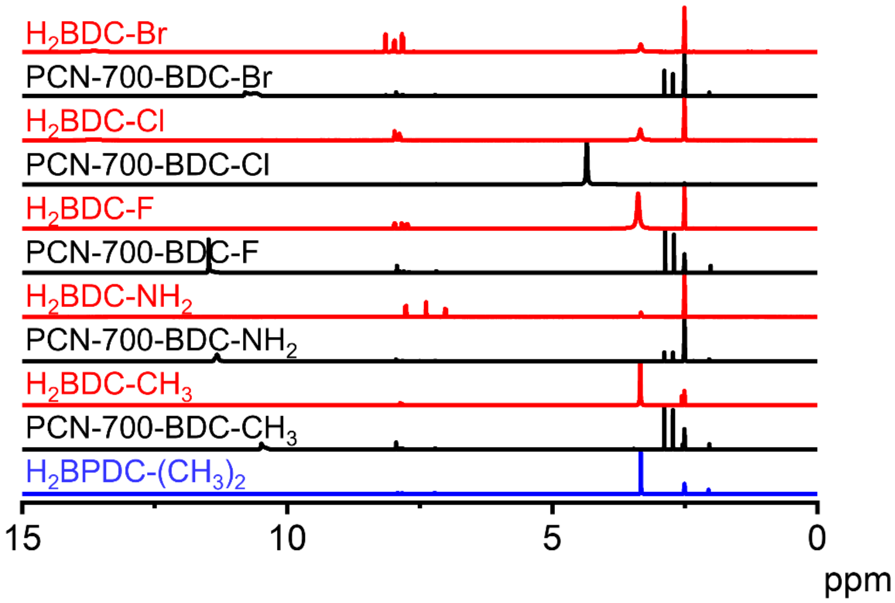

Figure S9. Liquid ^{1}H NMR spectra of $H_2BPDC-(CH_3)_2$, PCN-700-BDC-CH_3, $H_2BDC-CH_3$, PCN-700-BDC-NH_2, $H_2BDC-NH_2$, PCN-700-BDC-F, H_2BDC-F, PCN-700-BDC-Cl, H_2BDC-Cl, PCN-700-BDC-Br and H_2BDC-Br.

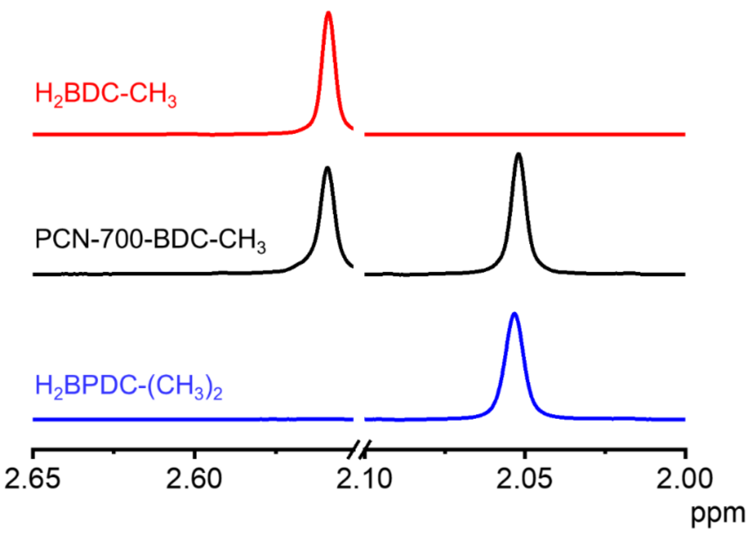

Figure S10. Liquid ^{1}H NMR spectra of $H_2BPDC-(CH_3)_2$, PCN-700-BDC-CH_3 and $H_2BDC-CH_3$.

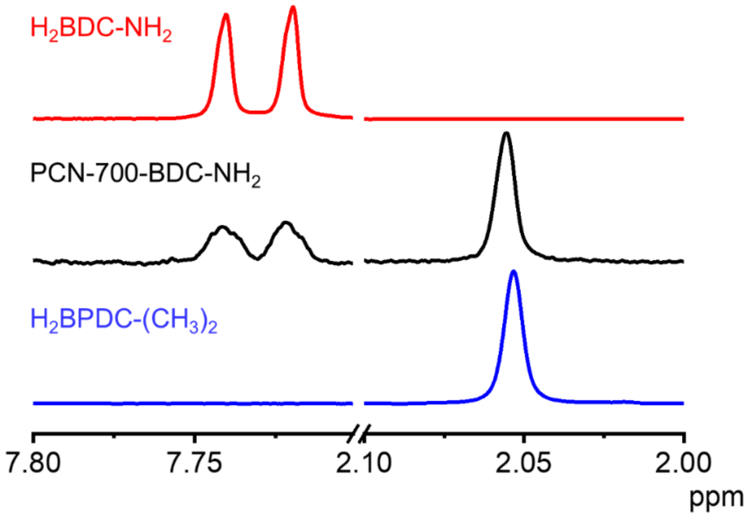

Figure S11. Liquid ^{1}H NMR spectra of $H_2BPDC-(CH_3)_2$, PCN-700-BDC-NH_2 and $H_2BDC-NH_2$.

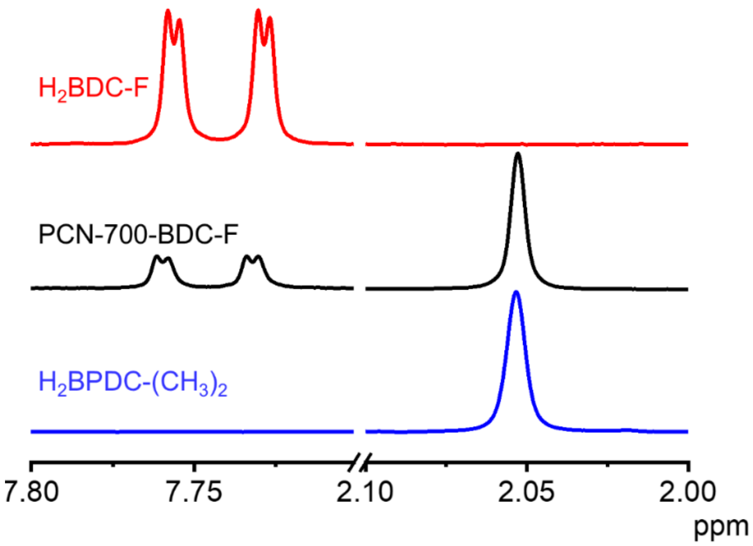

Figure S12. Liquid ^{1}H NMR spectra of $H_2BPDC-(CH_3)_2$, PCN-700-BDC-F and H_2BDC-F.

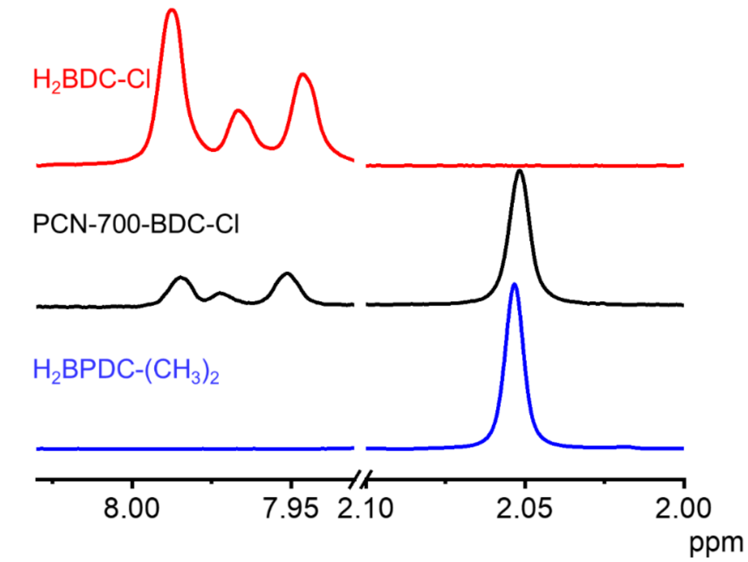

Figure S13. Liquid ^{1}H NMR spectra of $H_2BPDC-(CH_3)_2$, PCN-700-BDC-Cl and $H_2BDC-Cl$.

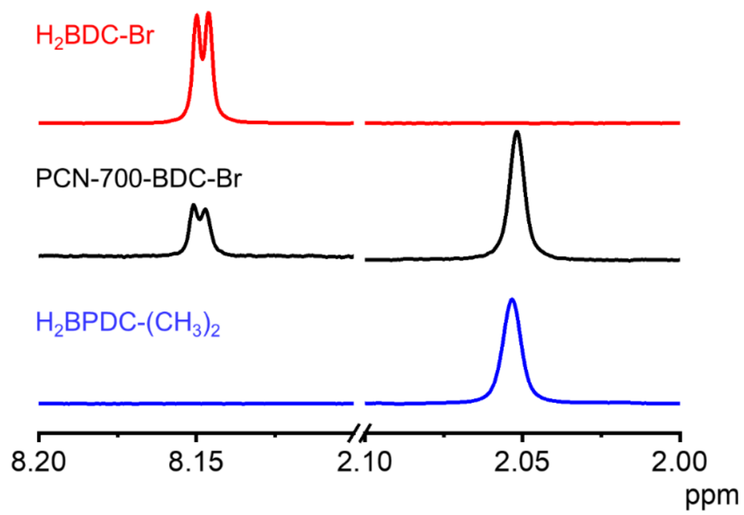

Figure S14. Liquid ^{1}H NMR spectra of $H_2BPDC-(CH_3)_2$, PCN-700-BDC-Br and H_2BDC-Br.

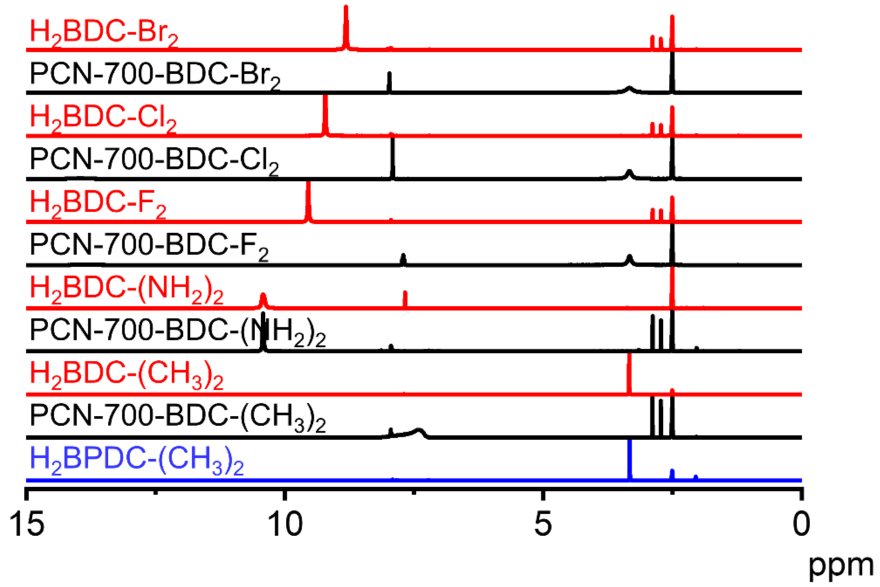

Figure S15. Liquid ^{1}H NMR spectra of $H_2BPDC-(CH_3)_2$, PCN-700-BDC-$(CH_3)_2$, $H_2BDC-(CH_3)_2$, PCN-700-BDC-$(NH_2)_2$, $H_2BDC-(NH_2)_2$, PCN-700-BDC-F_2, H_2BDC-F_2, PCN-700-BDC-Cl_2, $H_2BDC-Cl_2$, PCN-700-BDC-Br_2 and $H_2BDC-Br_2$.

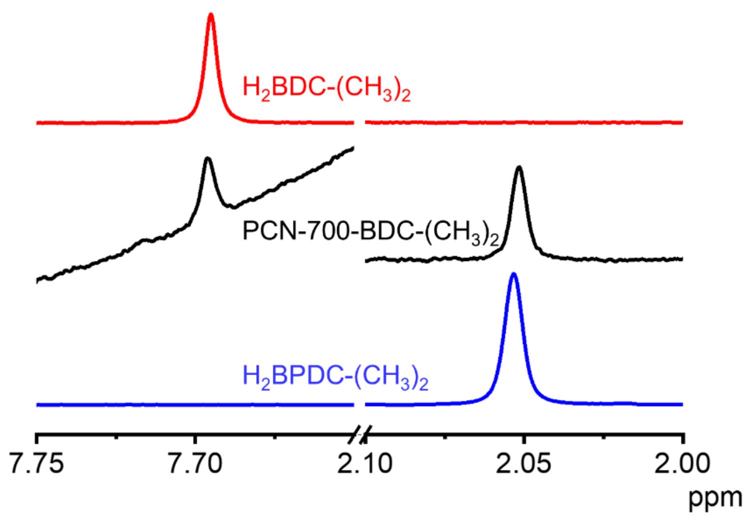

Figure S16. Liquid ^{1}H NMR spectra of $H_2BPDC-(CH_3)_2$, PCN-700-BDC-$(CH_3)_2$ and $H_2BDC-(CH_3)_2$.

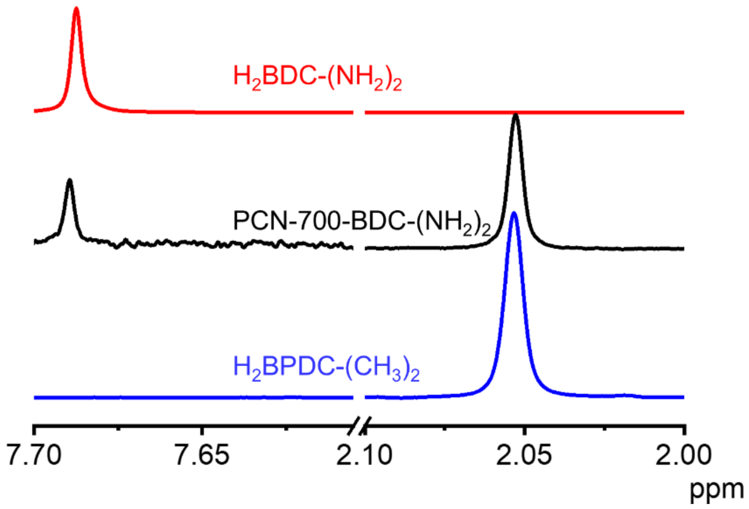

Figure S17. Liquid ^{1}H NMR spectra of $H_2BPDC-(CH_3)_2$, PCN-700-BDC-$(NH_2)_2$ and $H_2BDC-(NH_2)_2$.

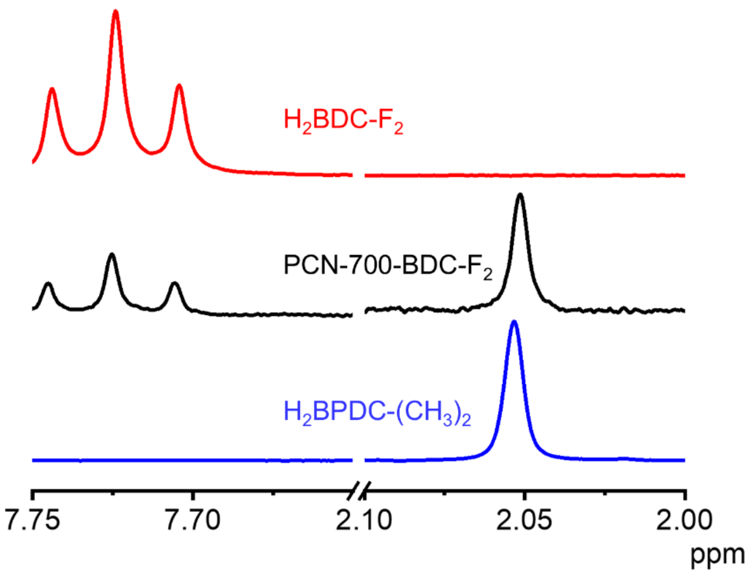

Figure S18. Liquid ^{1}H NMR spectra of H_2BPDC-$(CH_3)_2$, PCN-700-BDC-F_2 and H_2BDC-F_2.

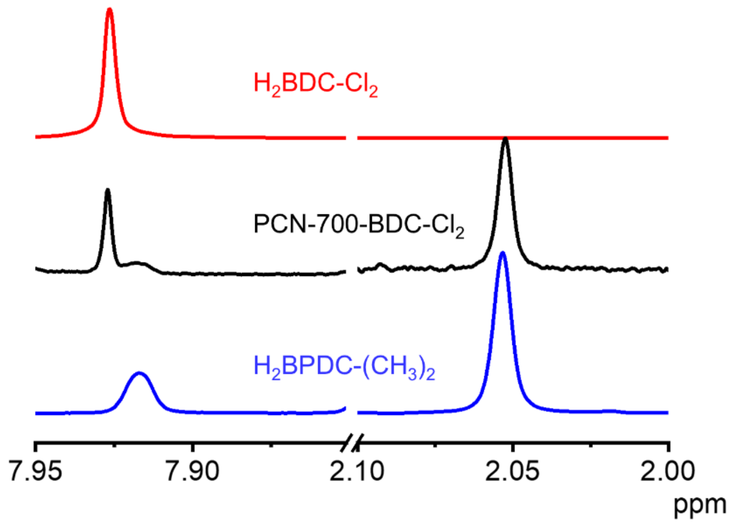

Figure S19. Liquid ^{1}H NMR spectra of H_2BPDC-$(CH_3)_2$, PCN-700-BDC-Cl_2 and H_2BDC-Cl_2.

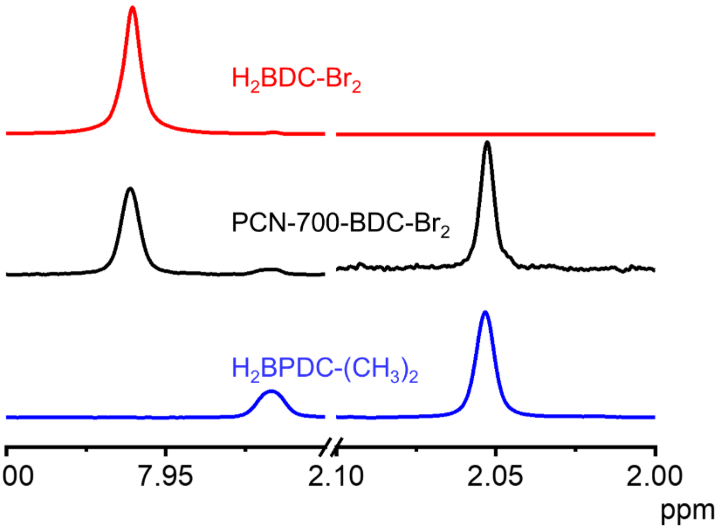

Figure S20. Liquid ^{1}H NMR spectra of H_2BPDC-$(CH_3)_2$, PCN-700-BDC-Br_2 and H_2BDC-Br_2.

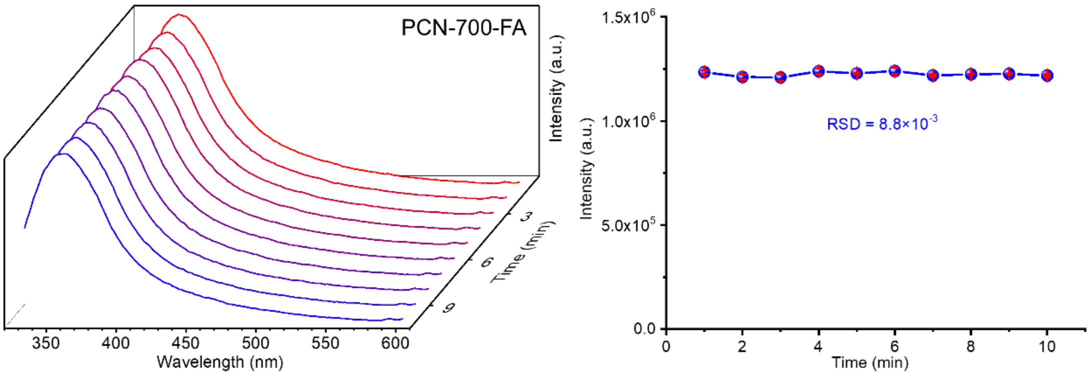

Figure S21. Emission spectra and intensity changes of PCN-700-FA with time.

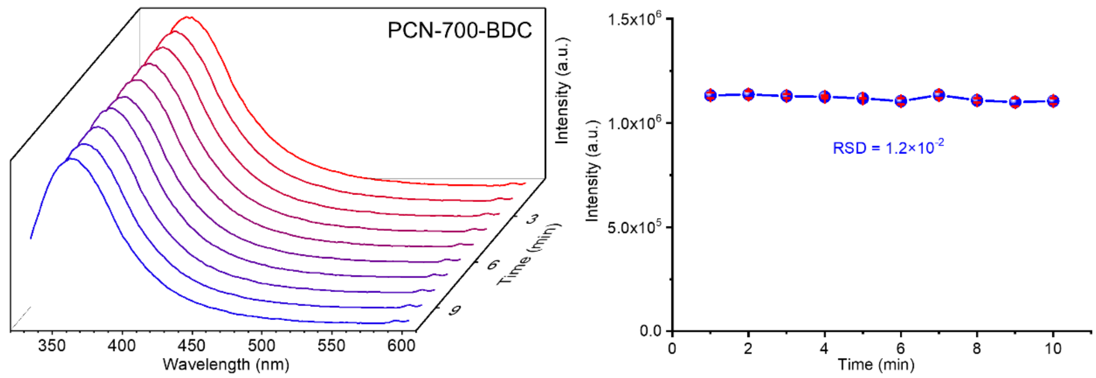

Figure S22. Emission spectra and intensity changes of PCN-700-BDC with time.

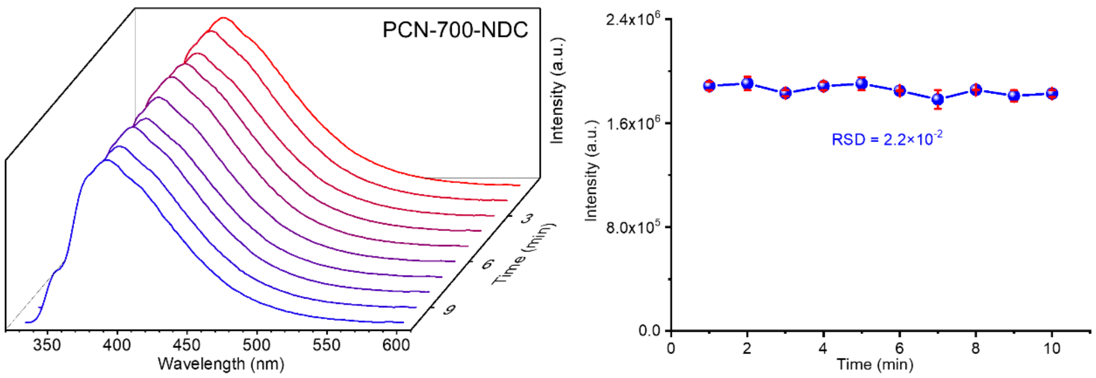

Figure S23. Emission spectra and intensity changes of PCN-700-NDC with time.

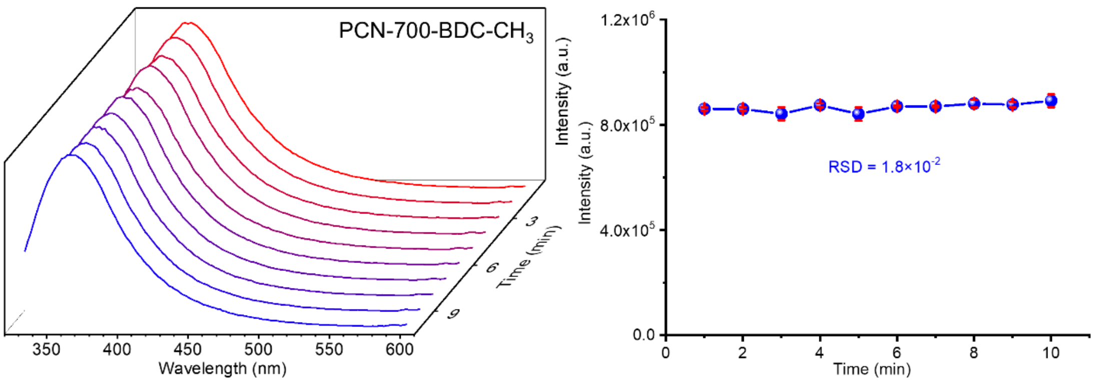

Figure S24. Emission spectra and intensity changes of PCN-700-BDC-CH_3 with time.

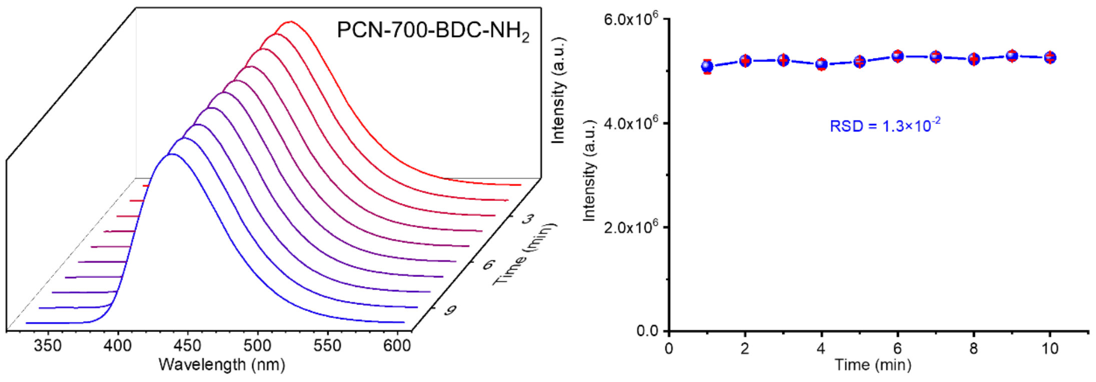

Figure S25. Emission spectra and intensity changes of PCN-700-BDC-NH$_2$ with time.

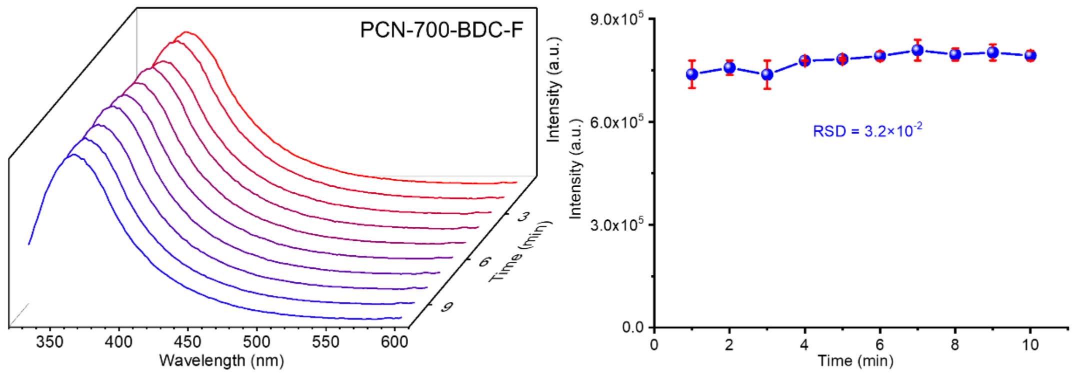

Figure S26. Emission spectra and intensity changes of PCN-700-BDC-F with time.

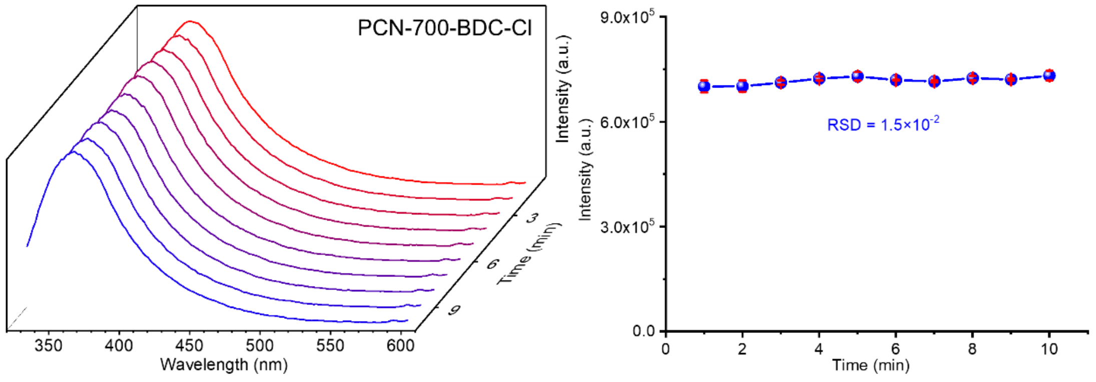

Figure S27. Emission spectra and intensity changes of PCN-700-BDC-Cl with time.

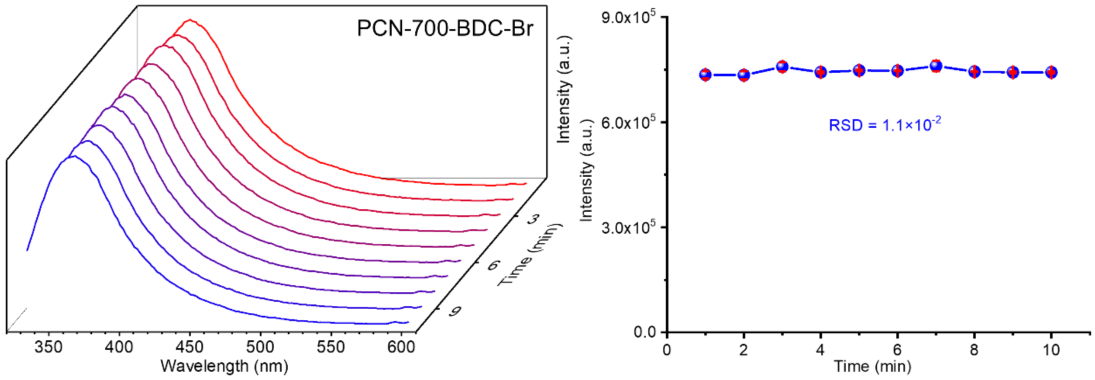

Figure S28. Emission spectra and intensity changes of PCN-700-BDC-Br with time.

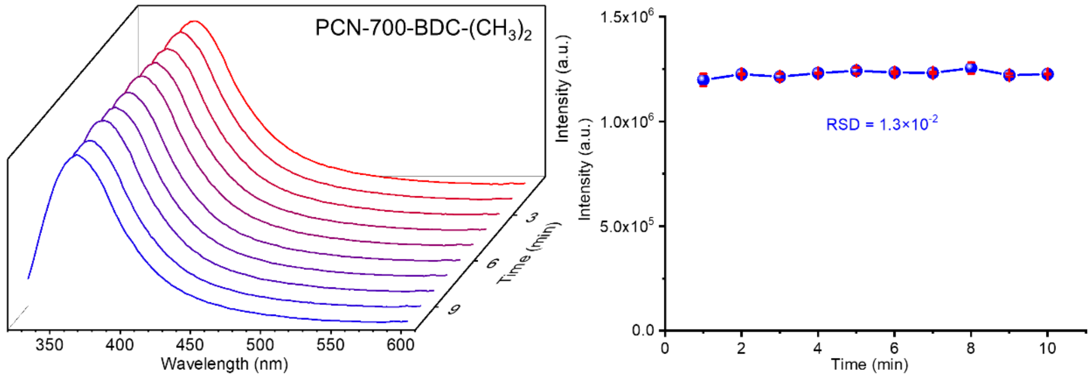

Figure S29. Emission spectra and intensity changes of PCN-700-BDC-$(CH_3)_2$ with time.

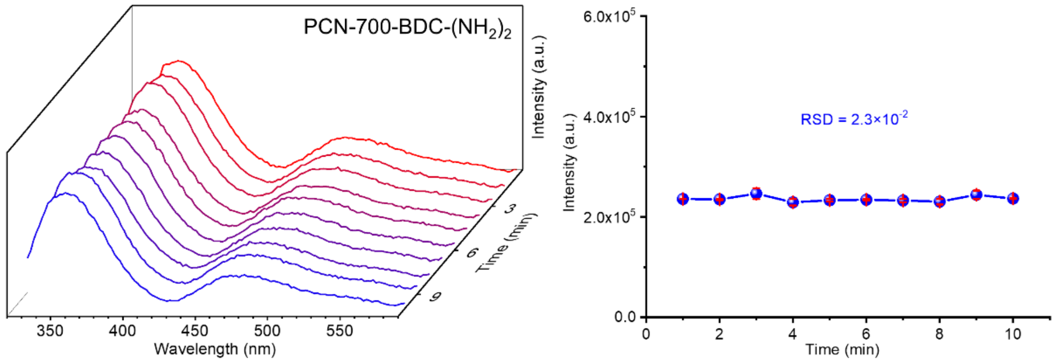

Figure S30. Emission spectra and intensity changes of PCN-700-BDC-$(NH_2)_2$ with time.

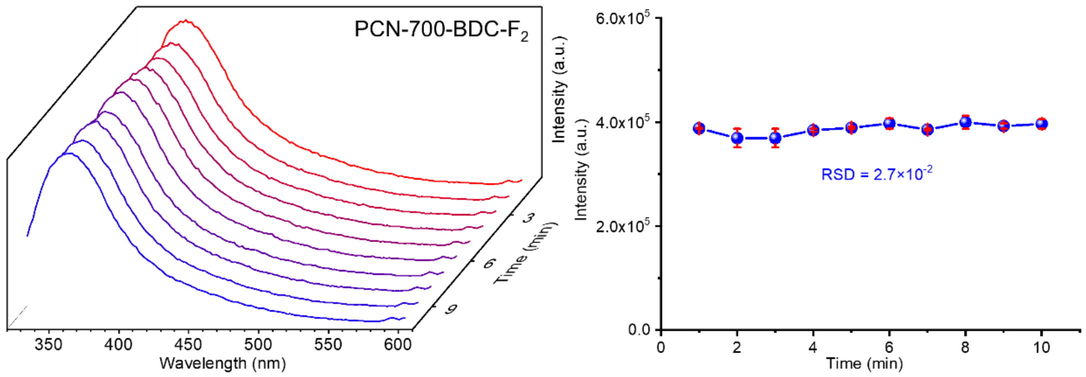

Figure S31. Emission spectra and intensity changes of PCN-700-BDC-F_2 with time.

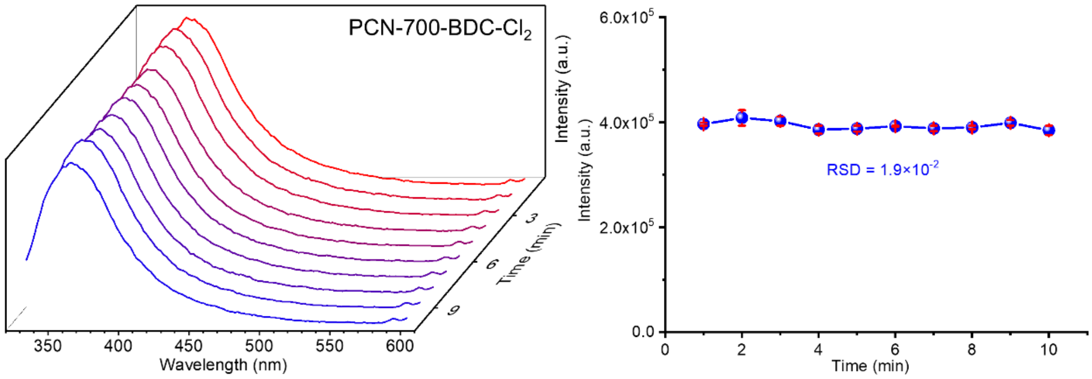

Figure S32. Emission spectra and intensity changes of PCN-700-BDC-Cl_2 with time.

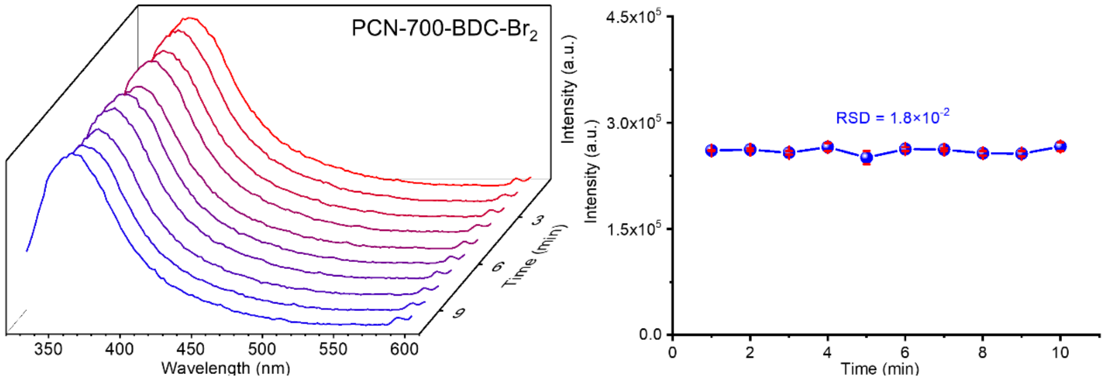

Figure S33. Emission spectra and intensity changes of PCN-700-BDC-Br_2 with time.

Luminescence Sensing

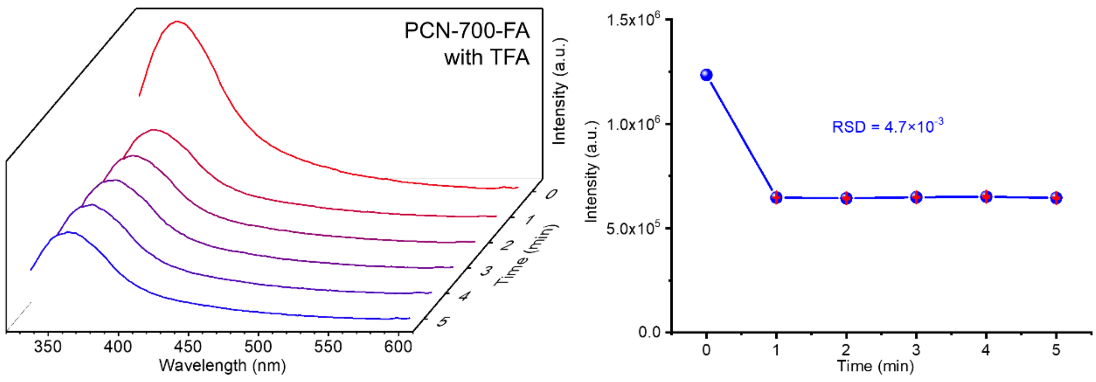

Figure S34. Emission spectra and intensity changes of PCN-700-FA with TFA.

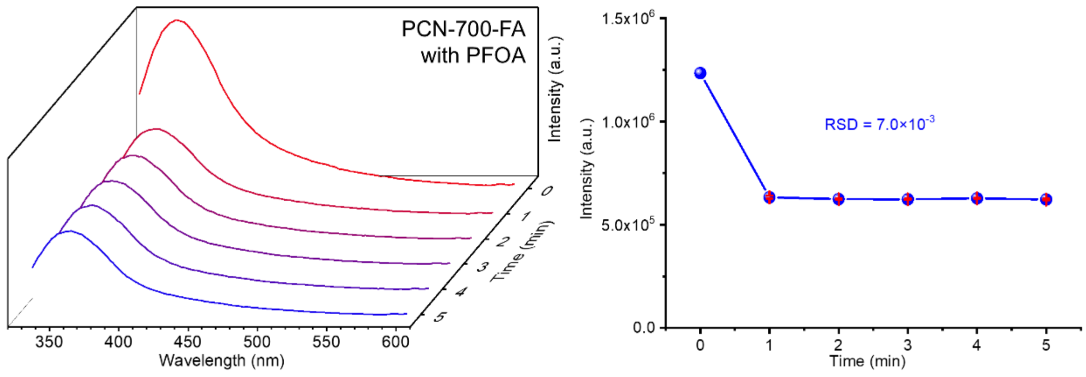

Figure S35. Emission spectra and intensity changes of PCN-700-FA with PFOA.

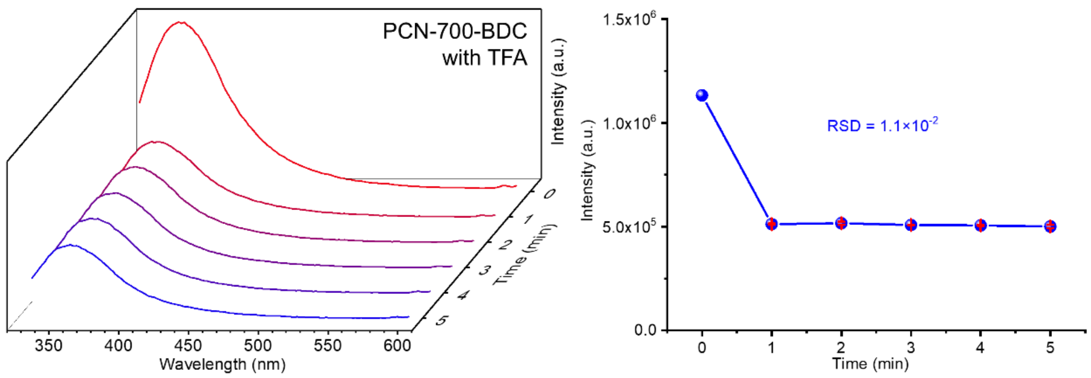

Figure S36. Emission spectra and intensity changes of PCN-700-BDC with TFA.

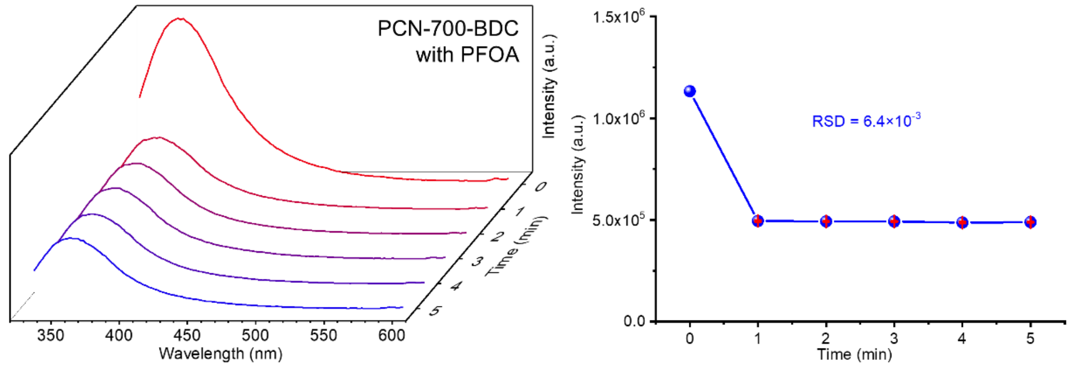

Figure S37. Emission spectra and intensity changes of PCN-700-BDC with PFOA.

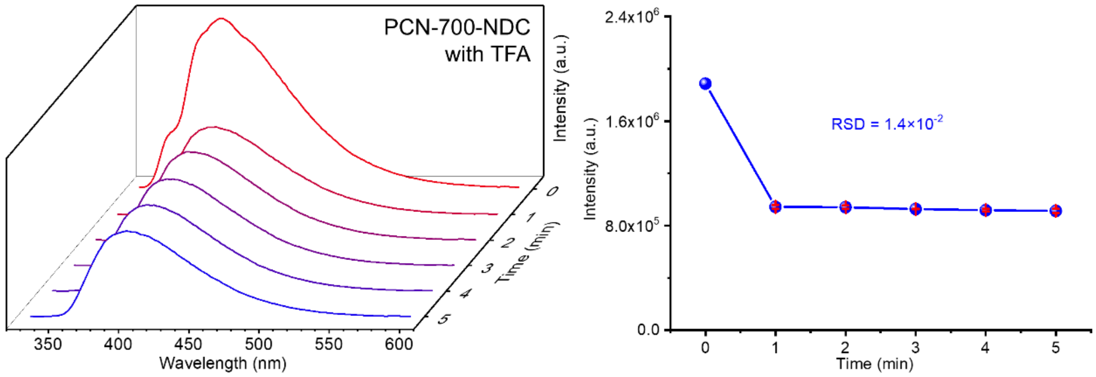

Figure S38. Emission spectra and intensity changes of PCN-700-NDC with TFA.

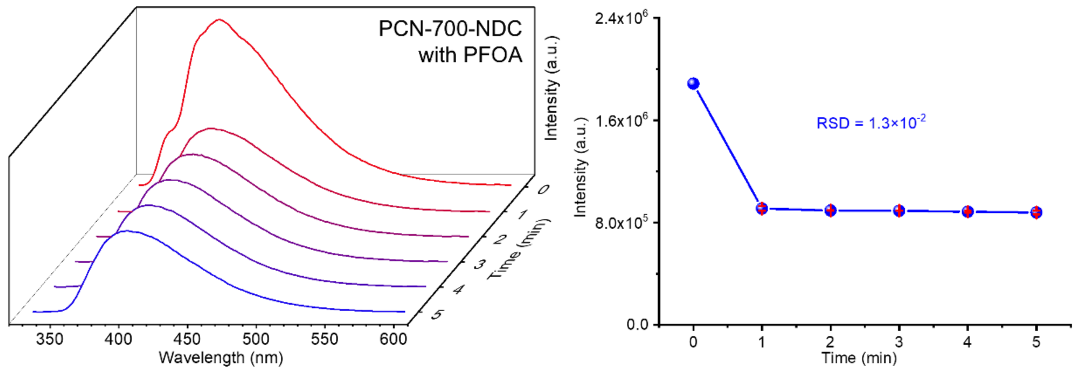

Figure S39. Emission spectra and intensity changes of PCN-700-NDC with PFOA.

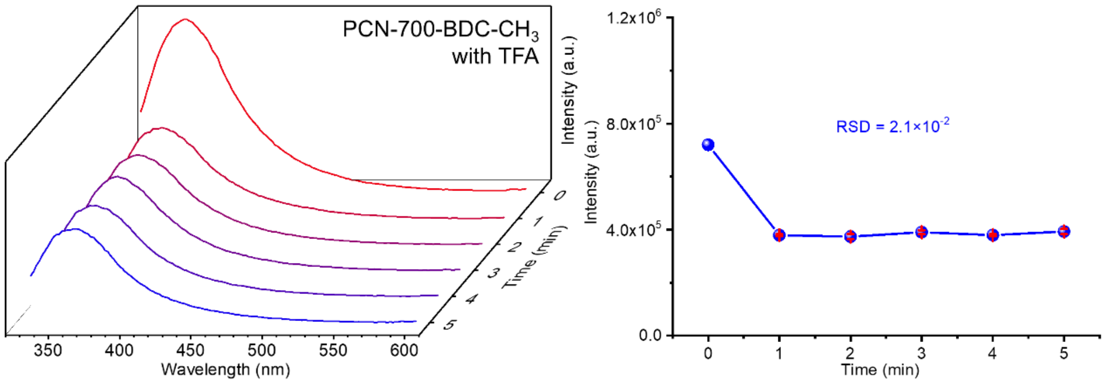

Figure S40. Emission spectra and intensity changes of PCN-700-BDC-CH_3 with TFA.

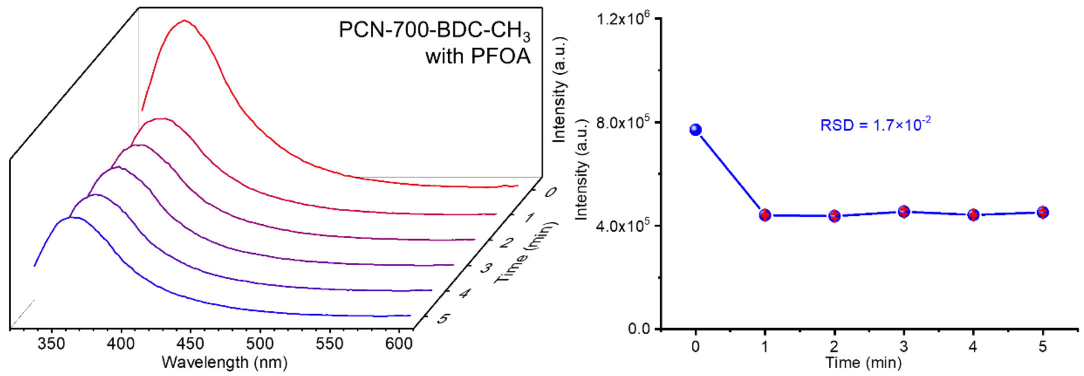

Figure S41. Emission spectra and intensity changes of PCN-700-BDC-CH_3 with PFOA.

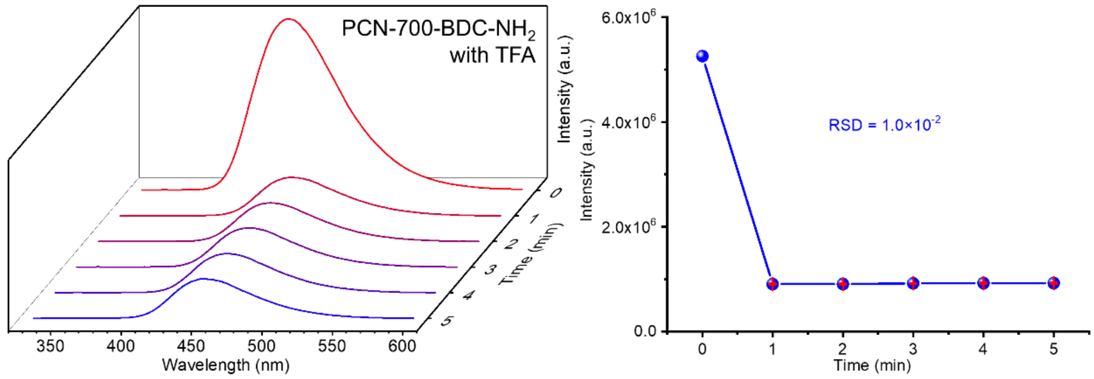

Figure S42. Emission spectra and intensity changes of PCN-700-BDC-NH_2 with TFA.

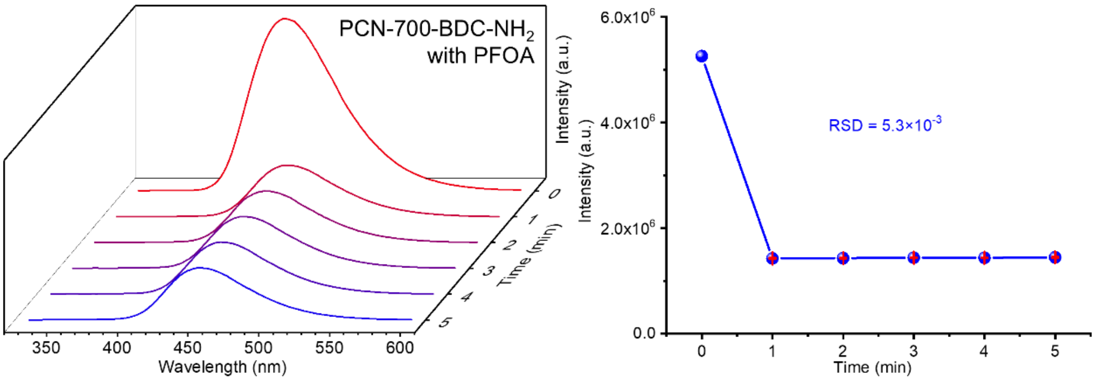

Figure S43. Emission spectra and intensity changes of PCN-700-BDC-NH2 with PFOA.

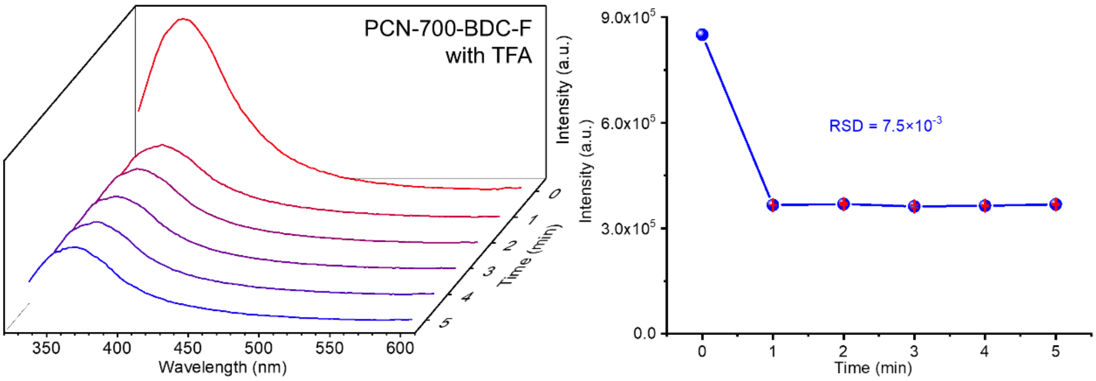

Figure S44. Emission spectra and intensity changes of PCN-700-BDC-F with TFA.

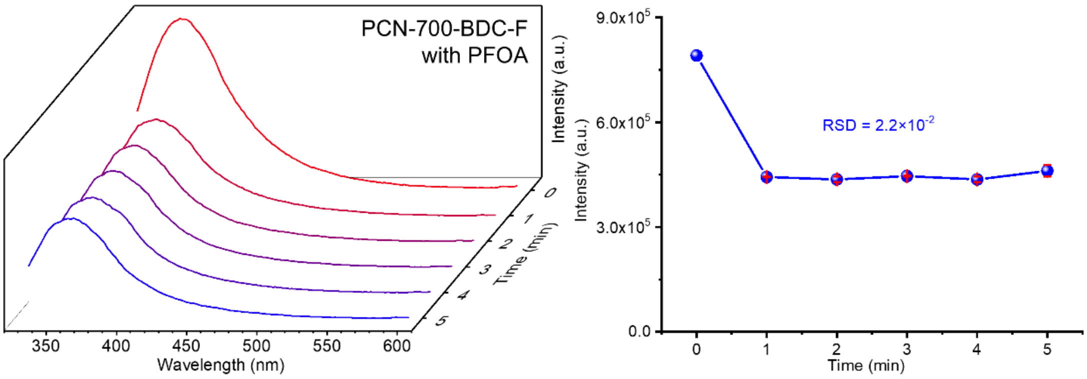

Figure S45. Emission spectra and intensity changes of PCN-700-BDC-F with PFOA.

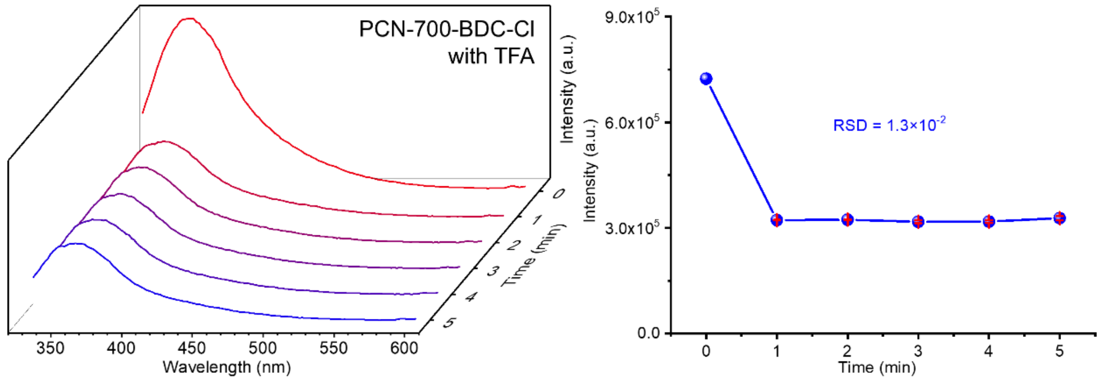

Figure S46. Emission spectra and intensity changes of PCN-700-BDC-Cl with TFA.

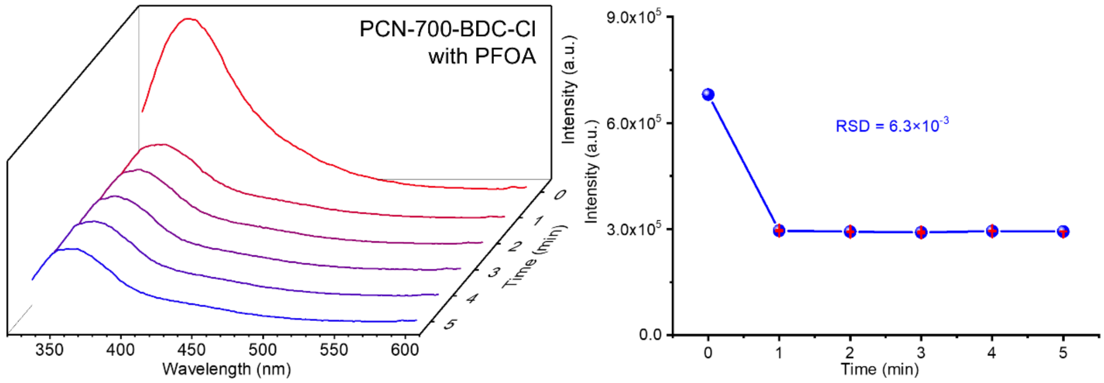

Figure S47. Emission spectra and intensity changes of PCN-700-BDC-Cl with PFOA.

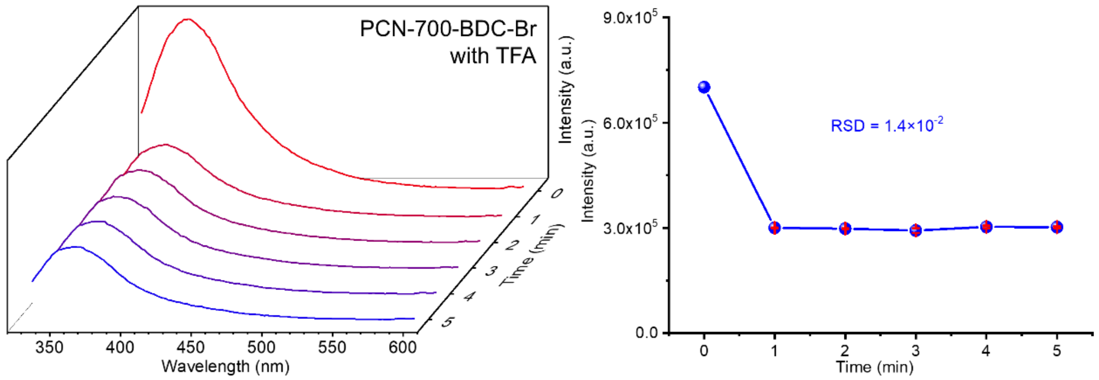

Figure S48. Emission spectra and intensity changes of PCN-700-BDC-Br with TFA.

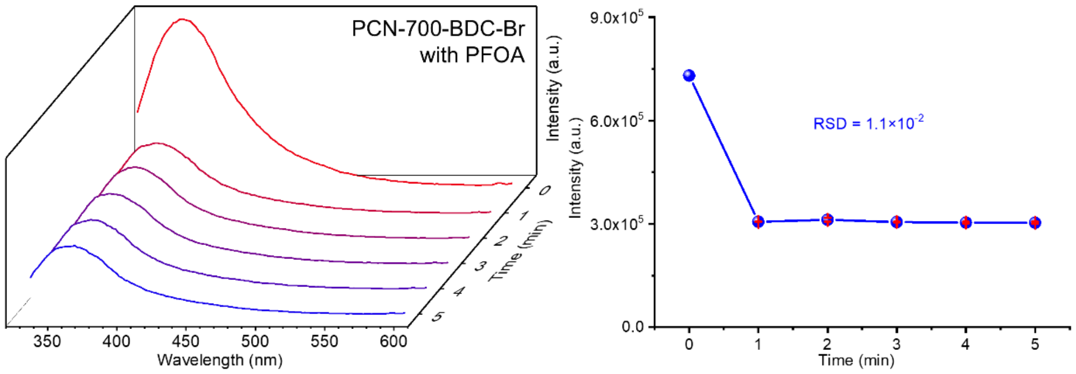

Figure S49. Emission spectra and intensity changes of PCN-700-BDC-Br with PFOA.

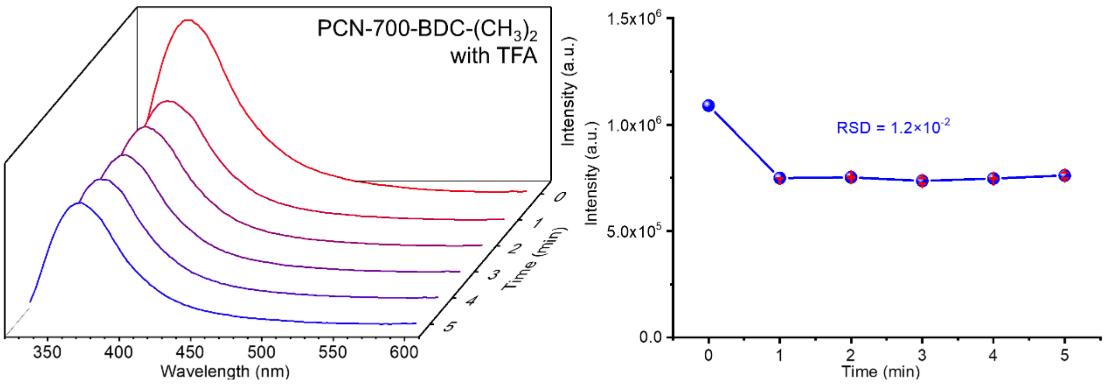

Figure S50. Emission spectra and intensity changes of PCN-700-BDC-$(CH_3)_2$ with TFA.

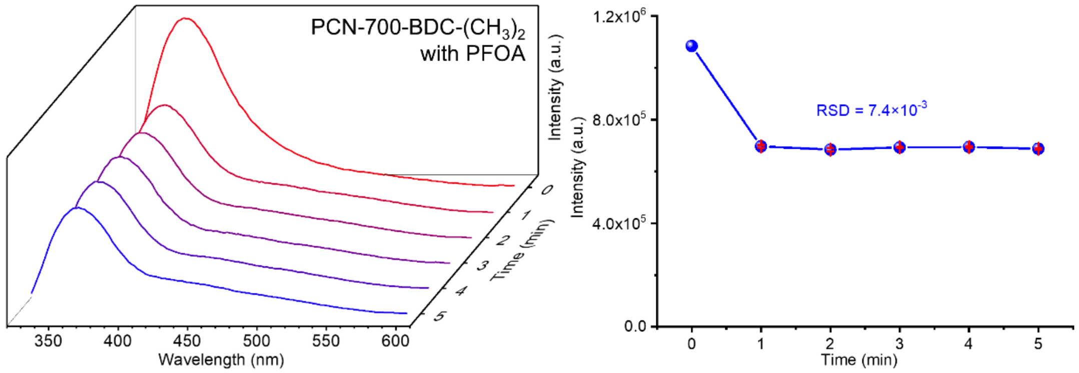

Figure S51. Emission spectra and intensity changes of PCN-700-BDC-$(CH_3)_2$ with PFOA.

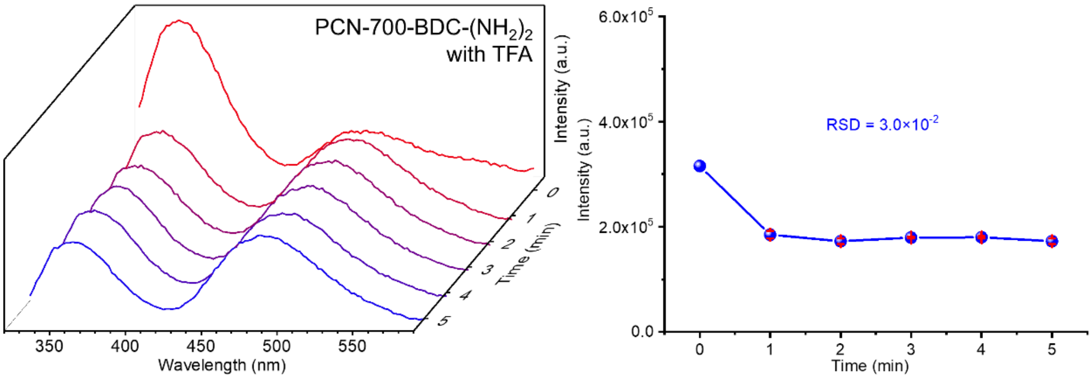

Figure S52. Emission spectra and intensity changes of PCN-700-BDC-$(NH_2)_2$ with TFA.

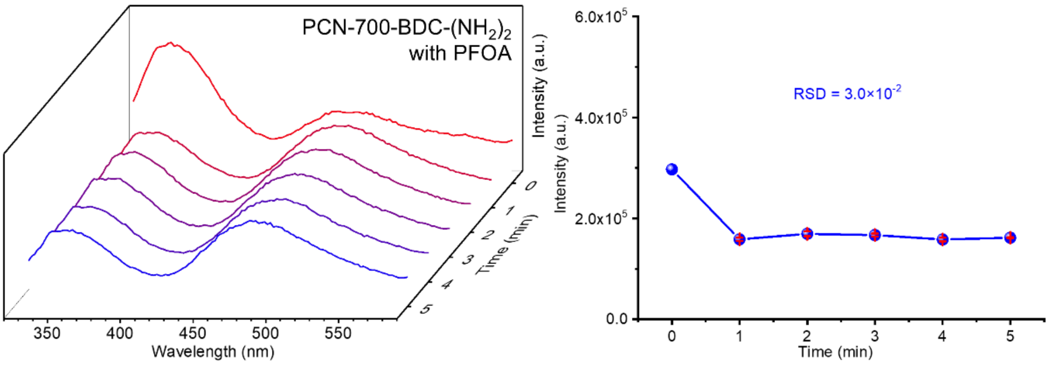

Figure S53. Emission spectra and intensity changes of PCN-700-BDC-$(NH_2)_2$ with PFOA.

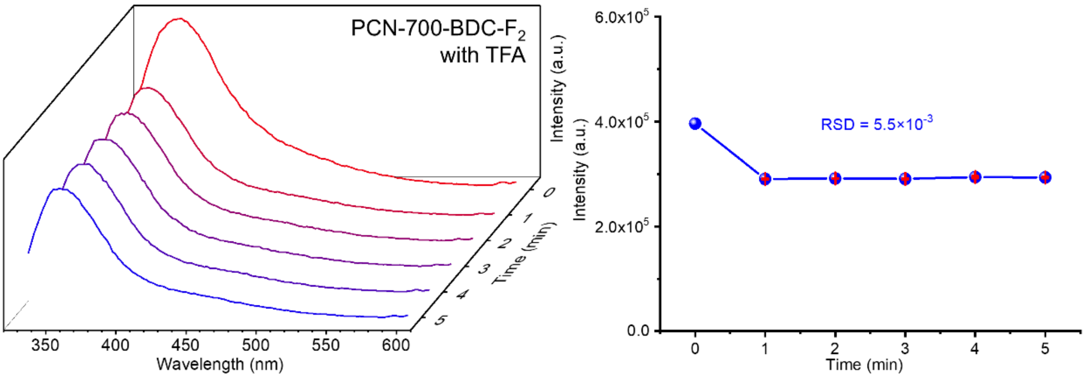

Figure S54. Emission spectra and intensity changes of PCN-700-BDC-F_2 with TFA.

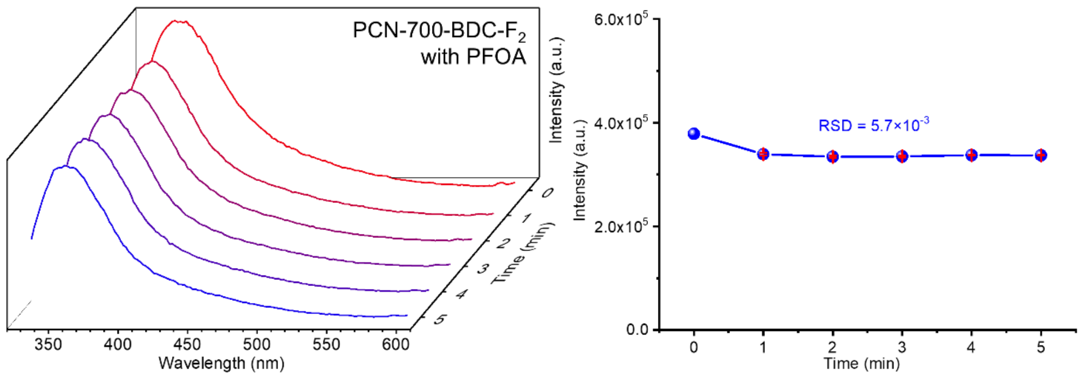

Figure S55. Emission spectra and intensity changes of PCN-700-BDC-F_2 with PFOA.

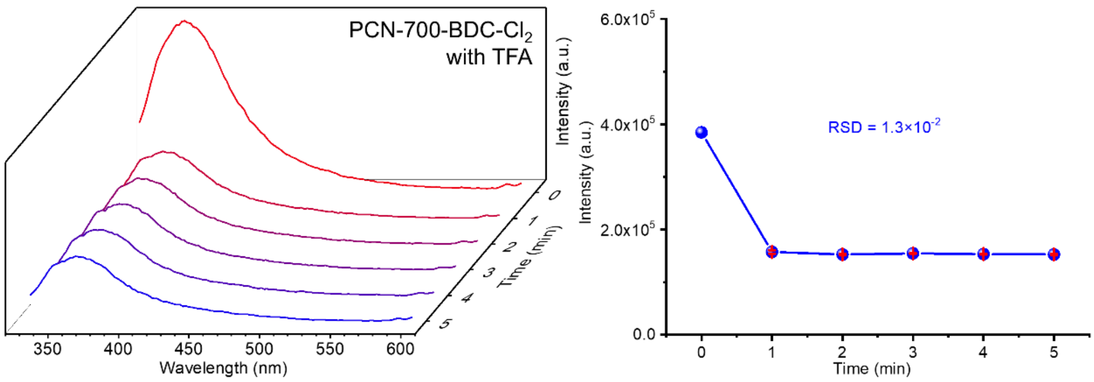

Figure S56. Emission spectra and intensity changes of PCN-700-BDC-Cl_2 with TFA.

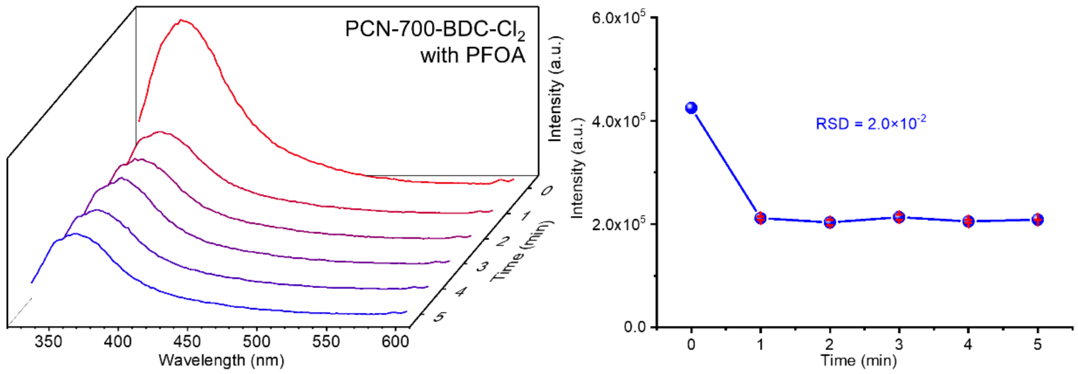

Figure S57. Emission spectra and intensity changes of PCN-700-BDC-Cl_2 with PFOA.

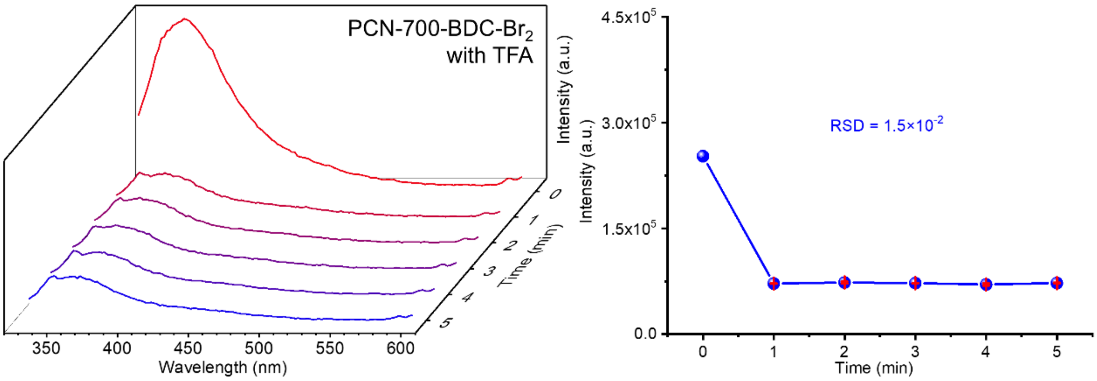

Figure S58. Emission spectra and intensity changes of PCN-700-BDC-Br_2 with TFA.

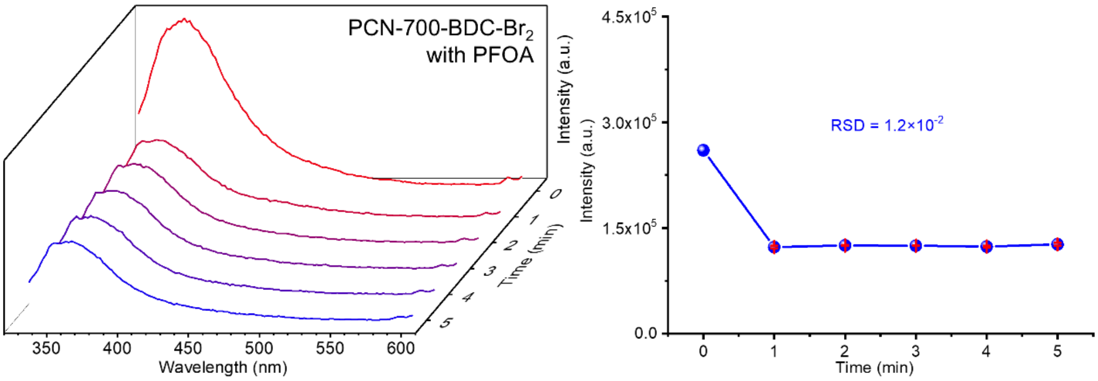

Figure S59. Emission spectra and intensity changes of PCN-700-BDC-Br_2 with PFOA.

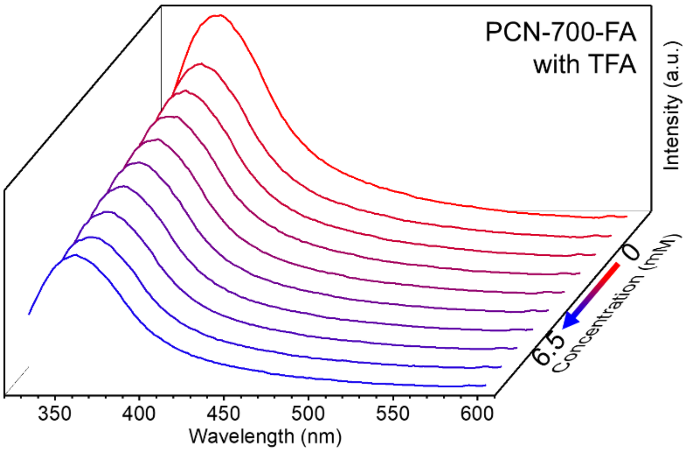

Figure S60. Emission spectra of PCN-700-FA with TFA.

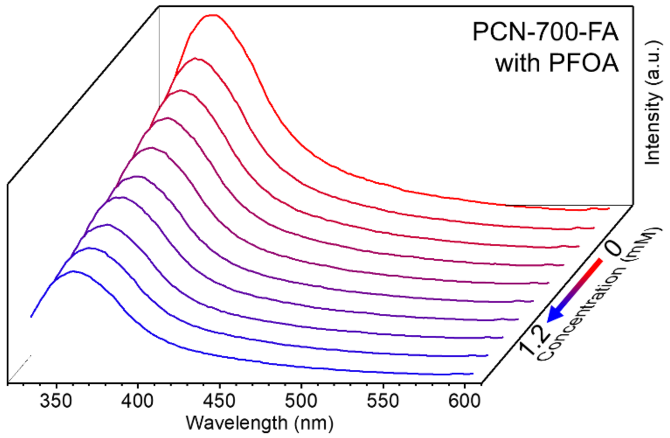

Figure S61. Emission spectra of PCN-700-FA with PFOA.

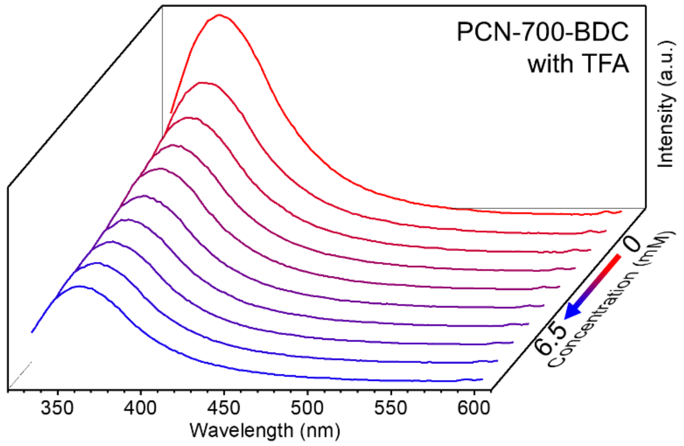

Figure S62. Emission spectra of PCN-700-BDC with TFA.

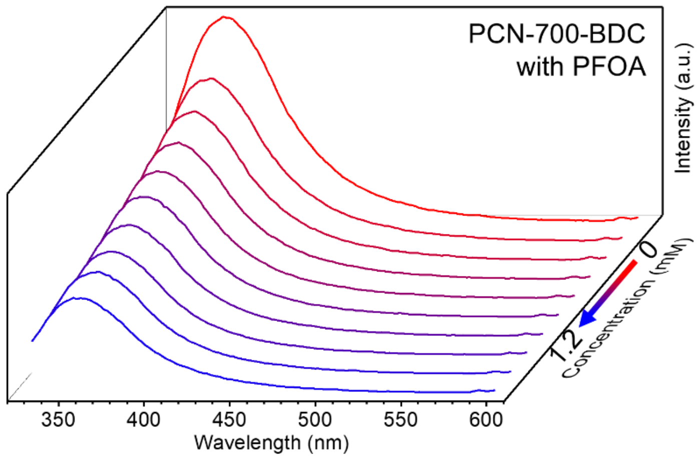

Figure S63. Emission spectra of PCN-700-BDC with PFOA.

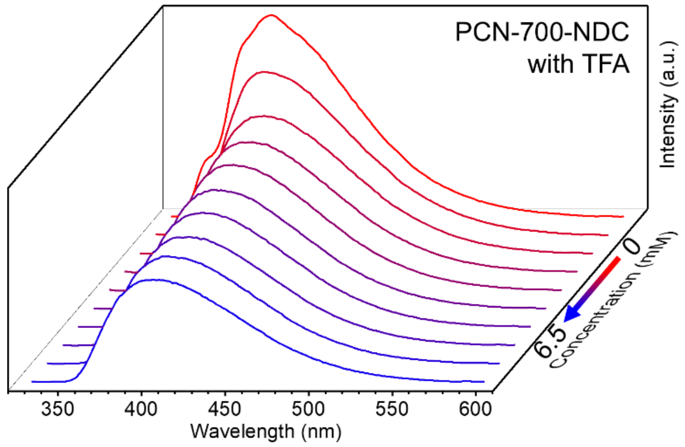

Figure S64. Emission spectra of PCN-700-NDC with TFA.

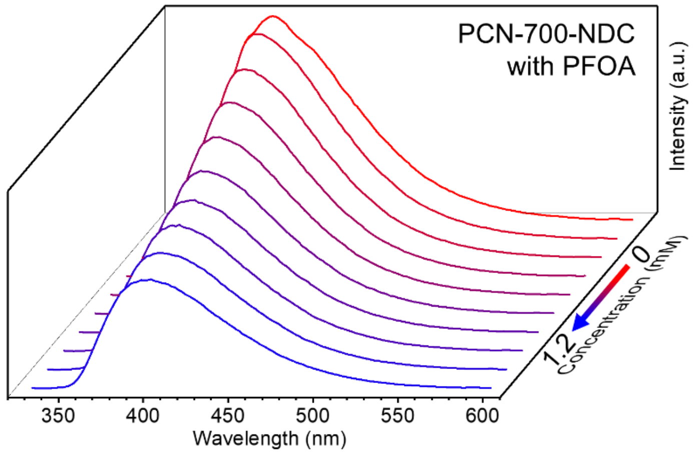

Figure S65. Emission spectra of PCN-700-NDC with PFOA.

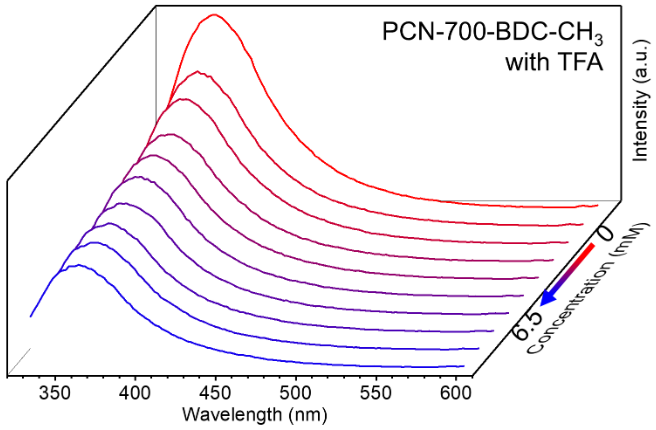

Figure S66. Emission spectra of PCN-700-BDC-CH_3 with TFA.

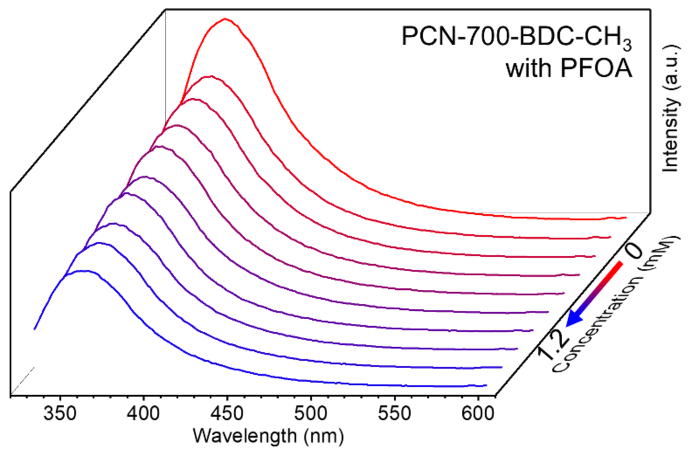

Figure S67. Emission spectra of PCN-700-BDC-CH_3 with PFOA.

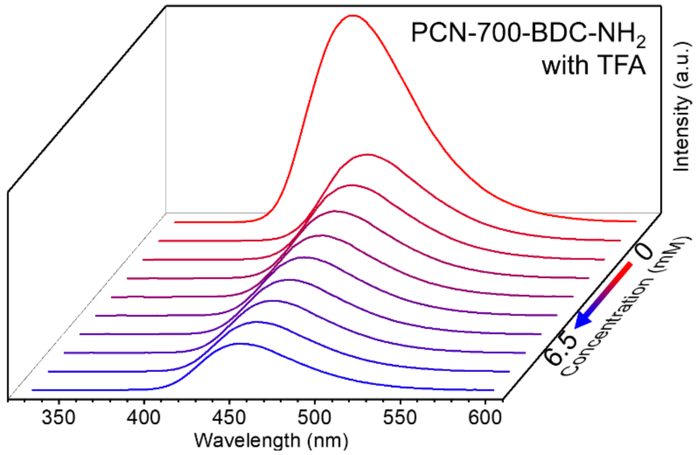

Figure S68. Emission spectra of PCN-700-BDC-NH_2 with TFA.

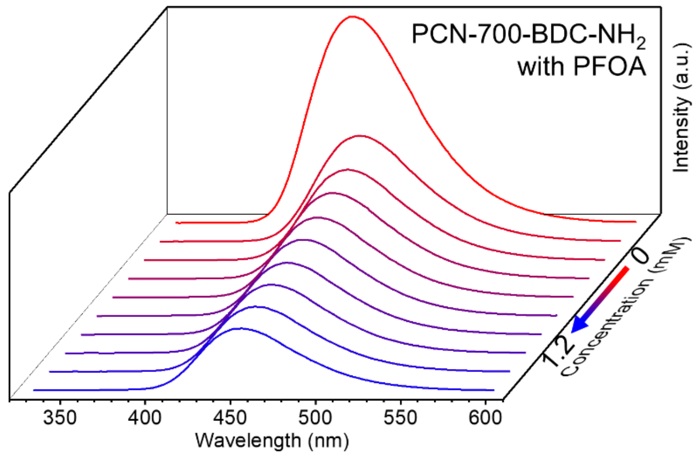

Figure S69. Emission spectra of PCN-700-BDC-NH_2 with PFOA.

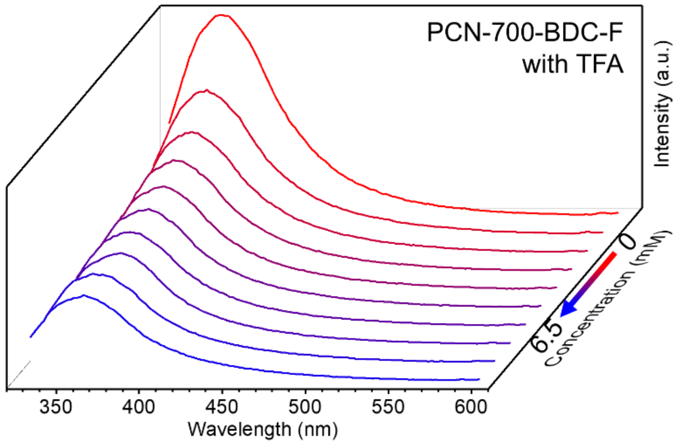

Figure S70. Emission spectra of PCN-700-BDC-F with TFA.

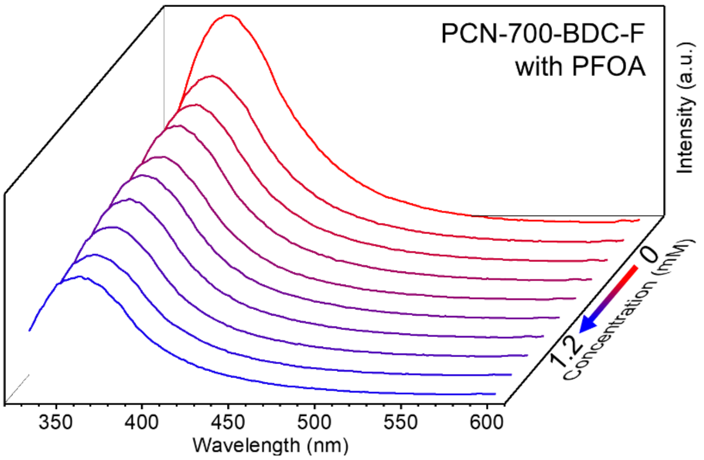

Figure S71. Emission spectra of PCN-700-BDC-F with PFOA.

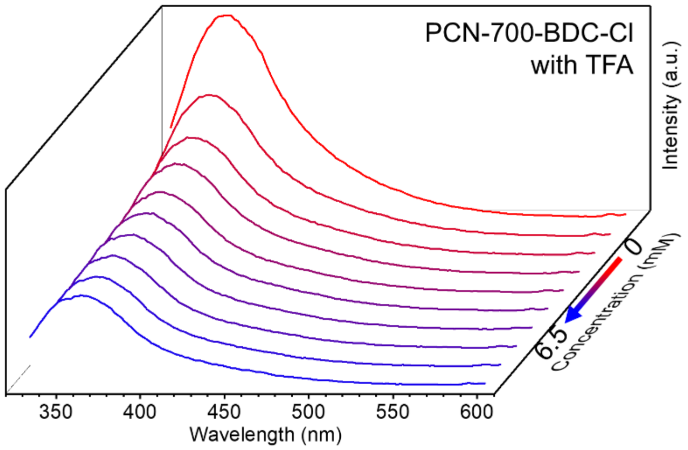

Figure S72. Emission spectra of PCN-700-BDC-Cl with TFA.

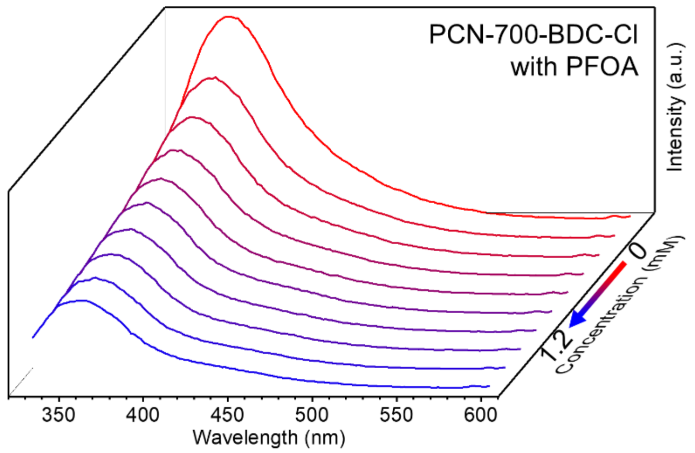

Figure S73. Emission spectra of PCN-700-BDC-Cl with PFOA.

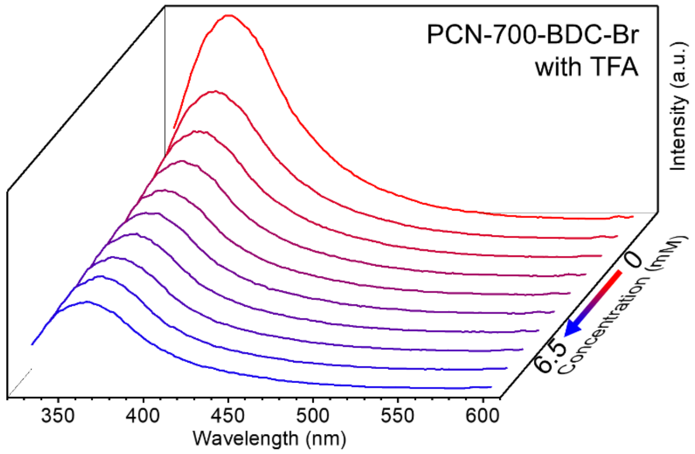

Figure S74. Emission spectra of PCN-700-BDC-Br with TFA.

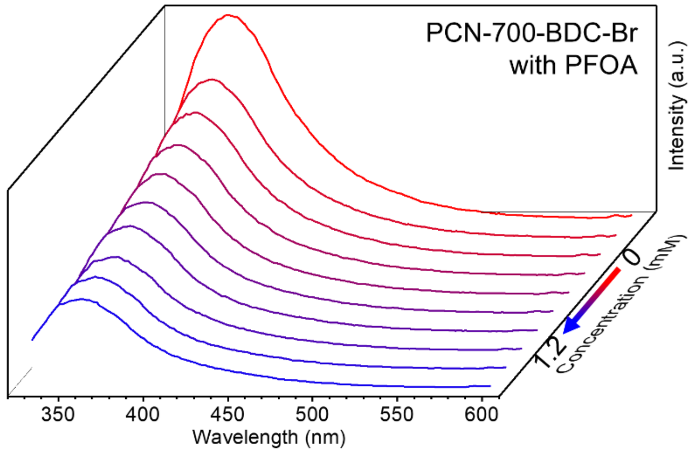

Figure S75. Emission spectra of PCN-700-BDC-Br with PFOA.

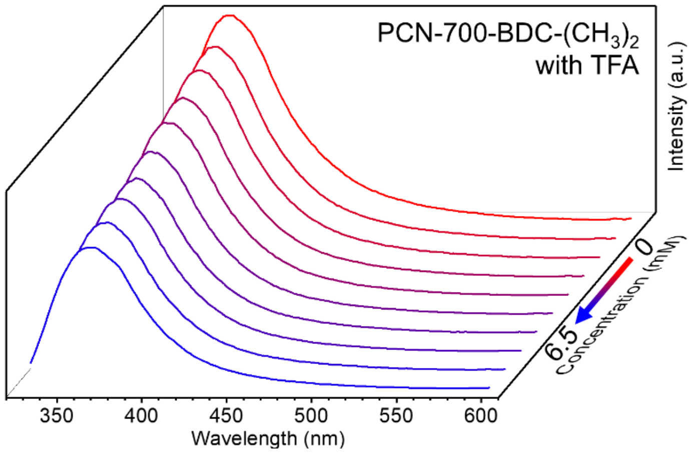

Figure S76. Emission spectra of PCN-700-BDC-(CH$_3$)$_2$ with TFA.

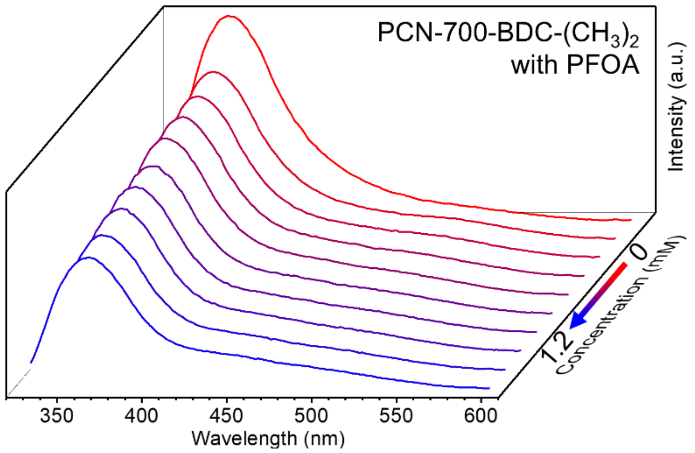

Figure S77. Emission spectra of PCN-700-BDC-(CH$_3$)$_2$ with PFOA.

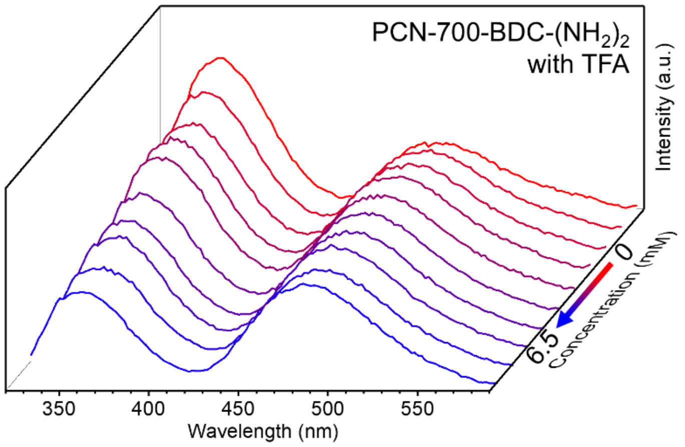

Figure S78. Emission spectra of PCN-700-BDC-(NH$_2$)$_2$ with TFA.

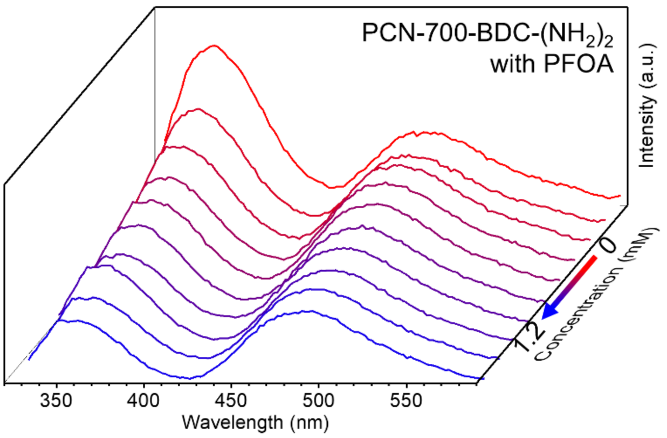

Figure S79. Emission spectra of PCN-700-BDC-$(NH_2)_2$ with PFOA.

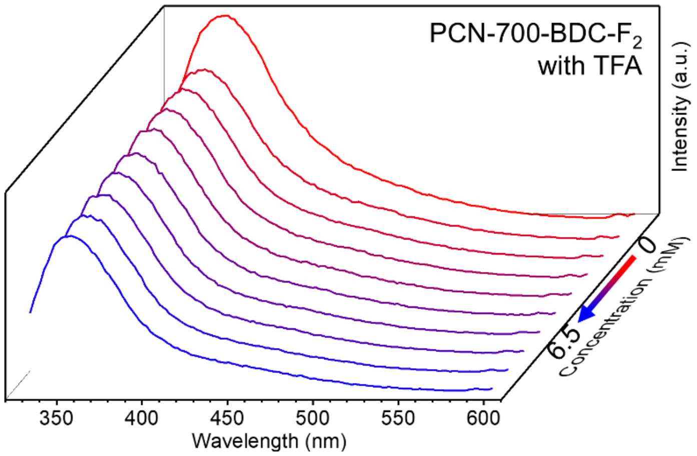

Figure S80. Emission spectra of PCN-700-BDC-F_2 with TFA.

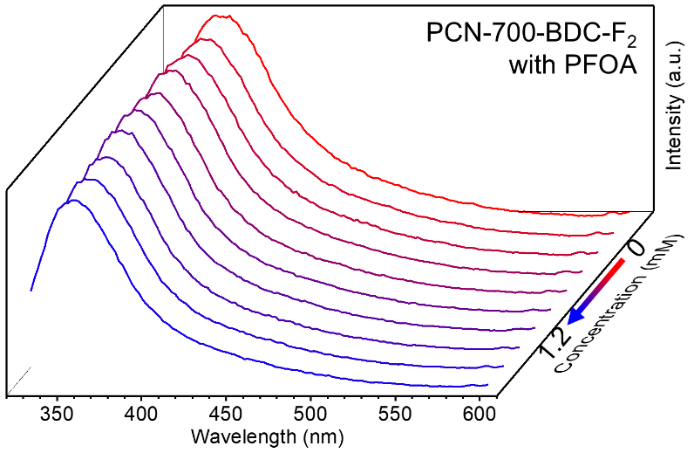

Figure S81. Emission spectra of PCN-700-BDC-F_2 with PFOA.

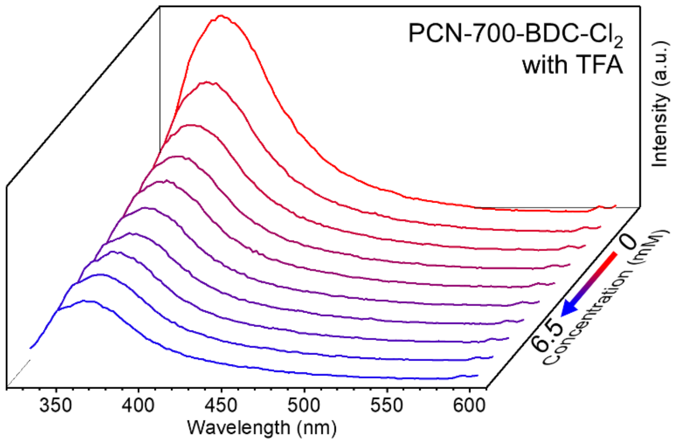

Figure S82. Emission spectra of PCN-700-BDC-Cl$_2$ with TFA.

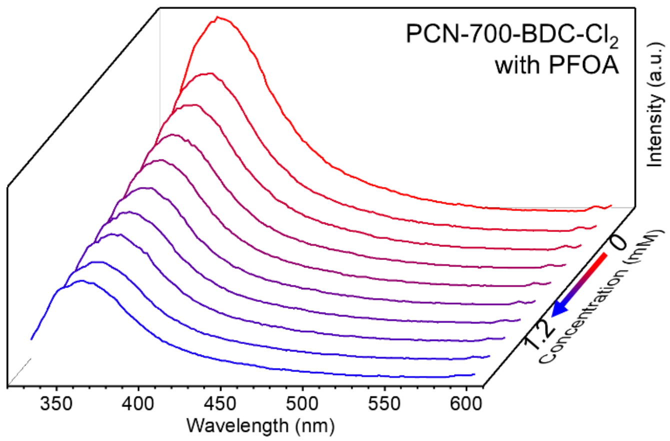

Figure S83. Emission spectra of PCN-700-BDC-Cl$_2$ with PFOA.

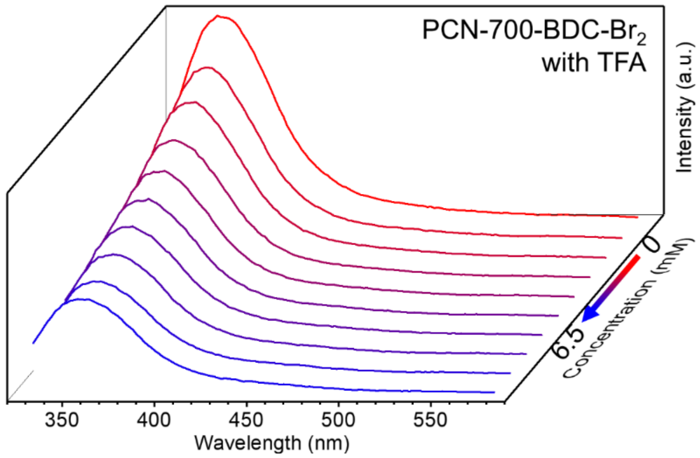

Figure S84. Emission spectra of PCN-700-BDC-Br$_2$ with TFA.

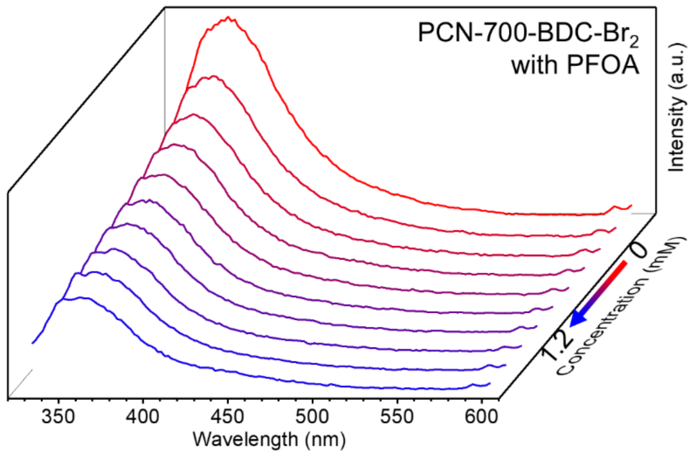

Figure S85. Emission spectra of PCN-700-BDC-Br2 with PFOA.

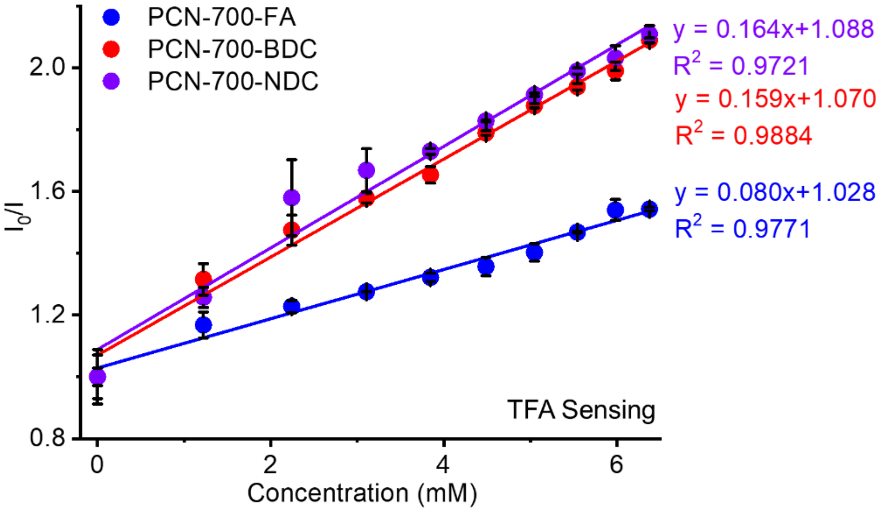

Figure S86. Intensity changes of PCN-700-FA, PCN-700-BDC and PCN-700-NDC with TFA.

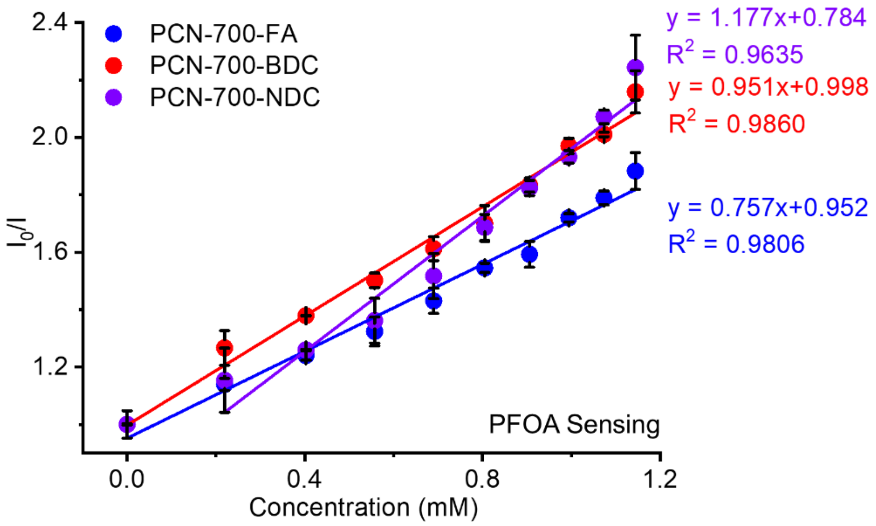

Figure S87. Intensity changes of PCN-700-FA, PCN-700-BDC and PCN-700-NDC with PFOA.

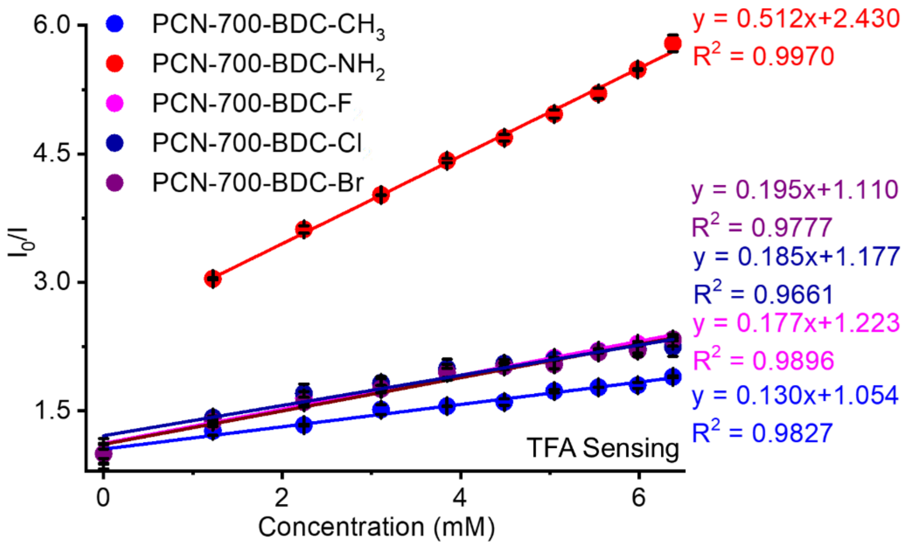

Figure S88. Intensity changes of PCN-700-BDC-CH_3, PCN-700-BDC-NH_2, PCN-700-BDC-F, PCN-700-BDC-Cl and PCN-700-BDC-Br with TFA.

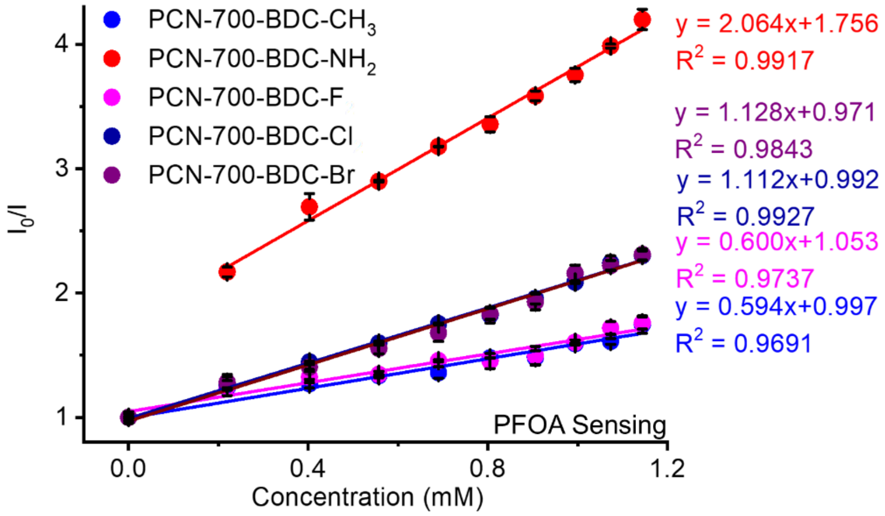

Figure S89. Intensity changes of PCN-700-BDC-CH_3, PCN-700-BDC-NH_2, PCN-700-BDC-F, PCN-700-BDC-Cl and PCN-700-BDC-Br with PFOA.

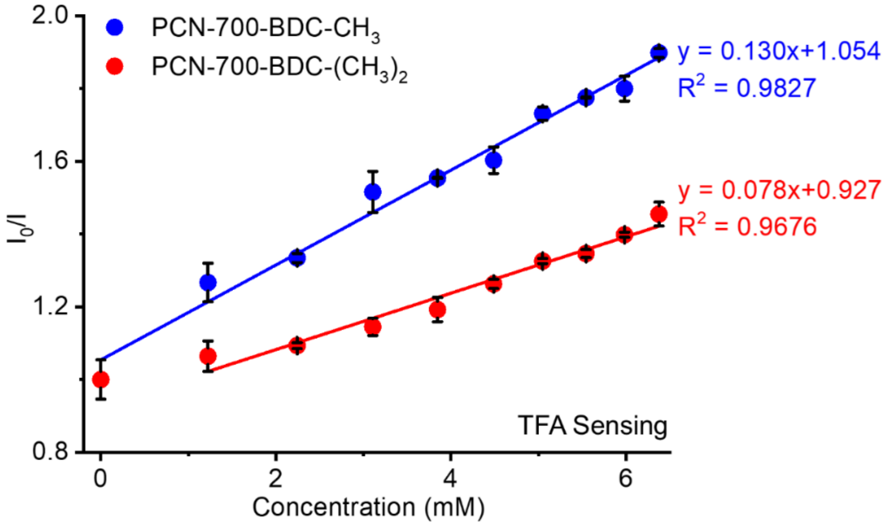

Figure S90. Intensity changes of PCN-700-BDC-CH_3 and PCN-700-BDC-$(CH_3)_2$ with TFA.

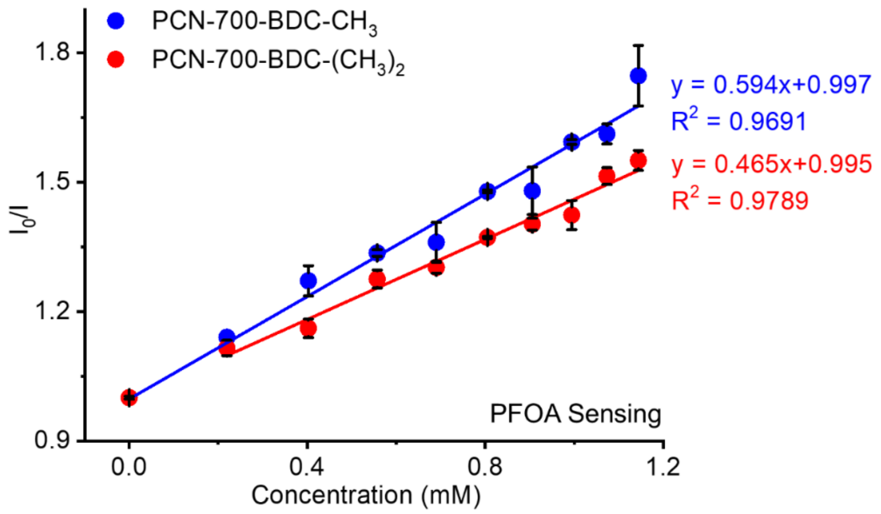

Figure S91. Intensity changes of PCN-700-BDC-CH_3 and PCN-700-BDC-$(CH_3)_2$ with PFOA.

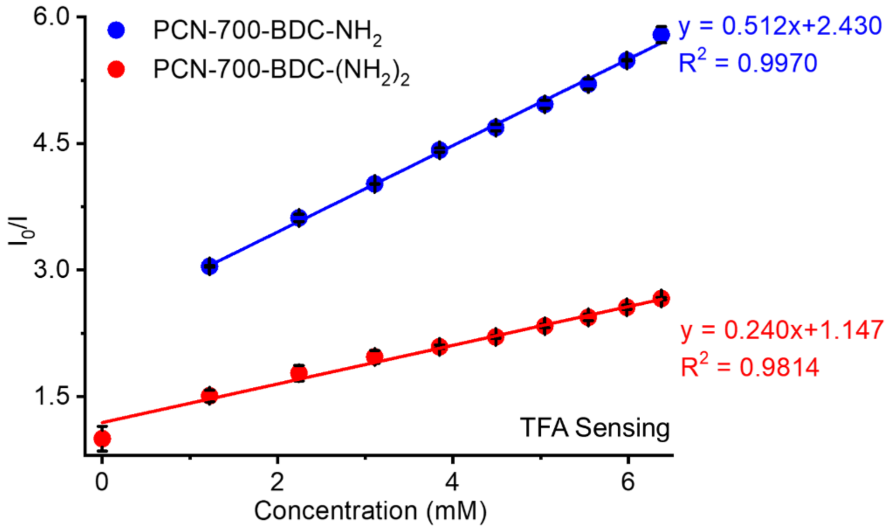

Figure S92. Intensity changes of PCN-700-BDC-NH_2 and PCN-700-BDC-$(NH_2)_2$ with TFA.

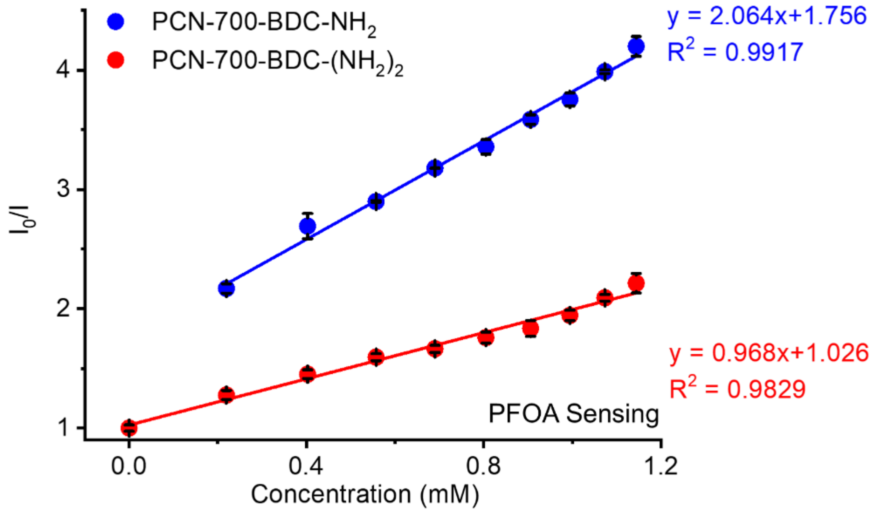

Figure S93. Intensity changes of PCN-700-BDC-NH_2 and PCN-700-BDC-$(NH_2)_2$ with PFOA.

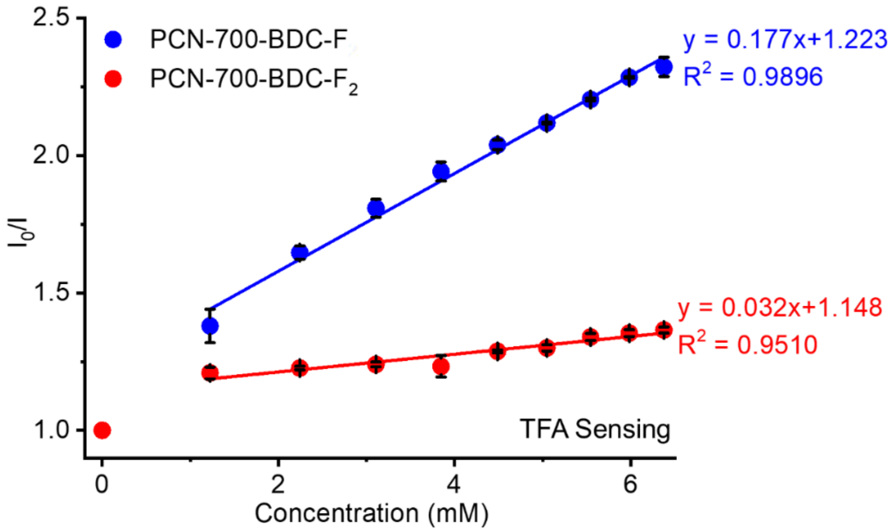

Figure S94. Intensity changes of PCN-700-BDC-F and PCN-700-BDC-F_2 with TFA.

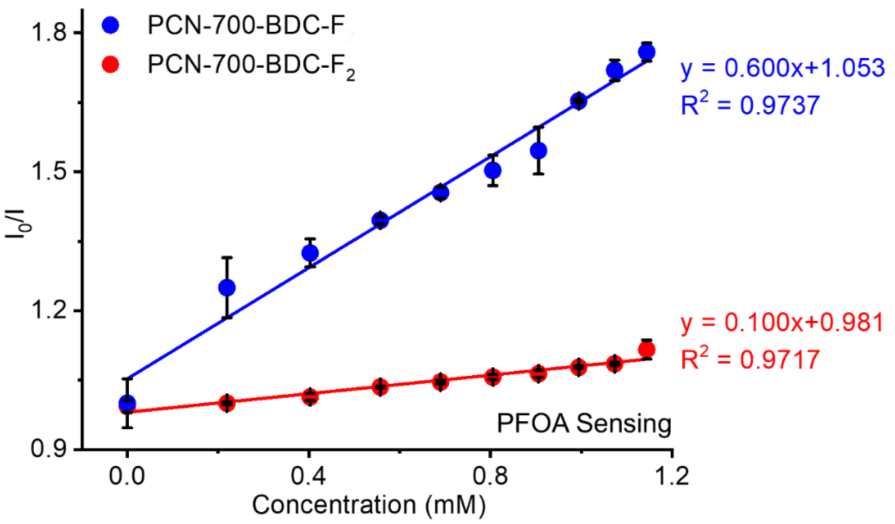

Figure S95. Intensity changes of PCN-700-BDC-F and PCN-700-BDC-F2 with PFOA.

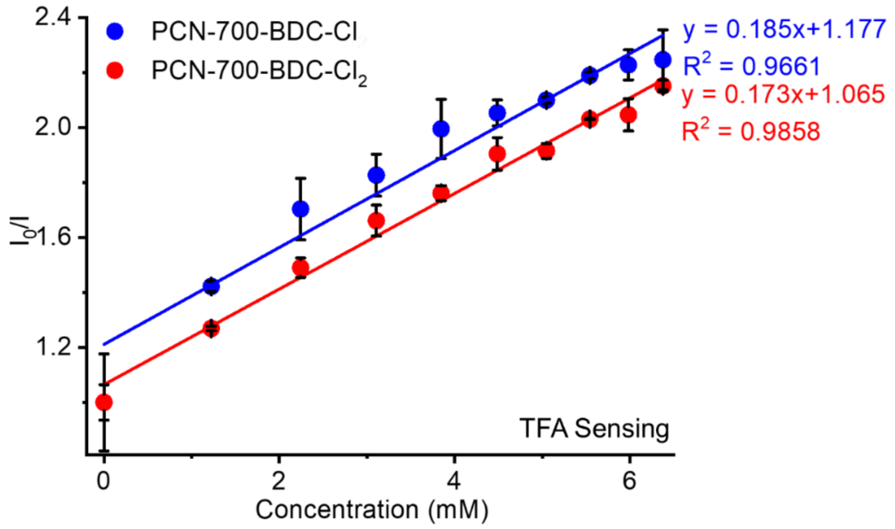

Figure S96. Intensity changes of PCN-700-BDC-Cl and PCN-700-BDC-Cl2 with TFA.

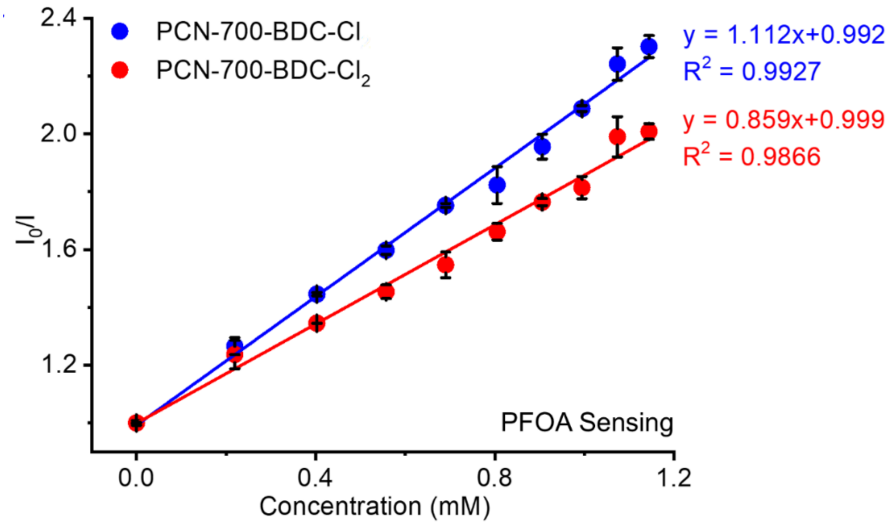

Figure S97. Intensity changes of PCN-700-BDC-Cl and PCN-700-BDC-Cl_2 with PFOA.

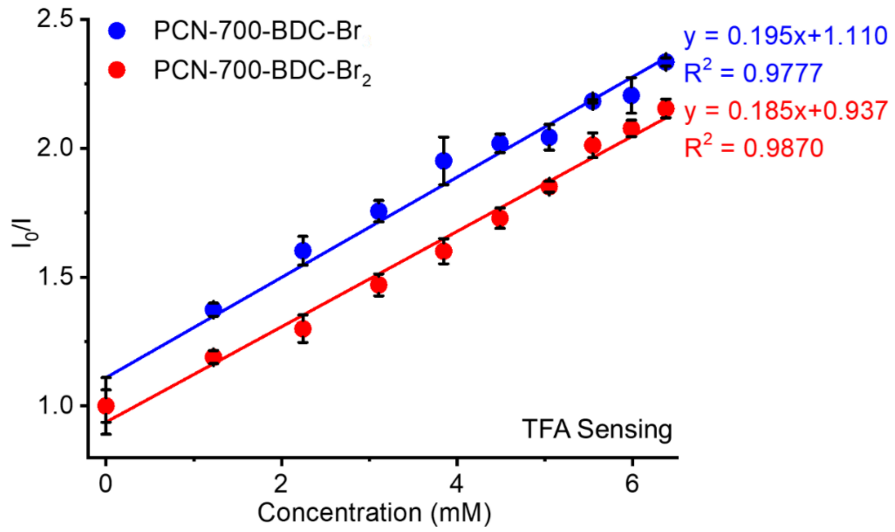

Figure S98. Intensity changes of PCN-700-BDC-Br and PCN-700-BDC-Br_2 with TFA.

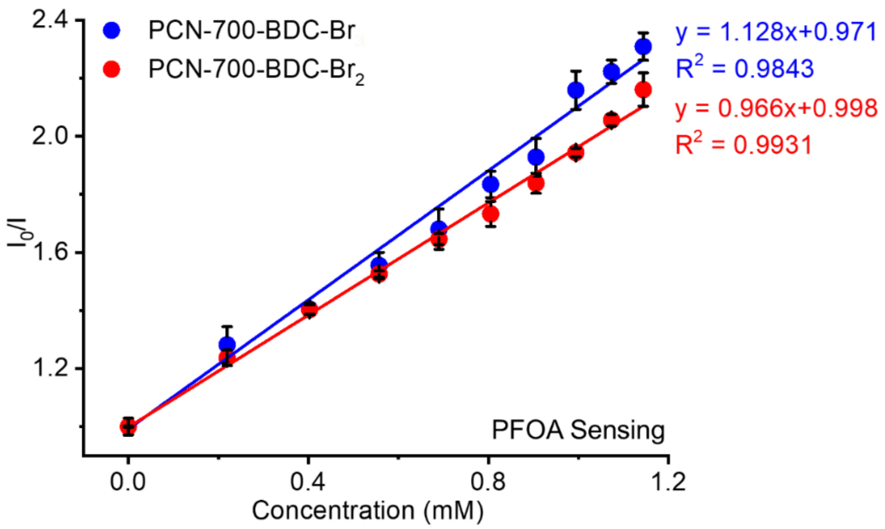

Figure S99. Intensity changes of PCN-700-BDC-Br and PCN-700-BDC-Br$_2$ with PFOA.

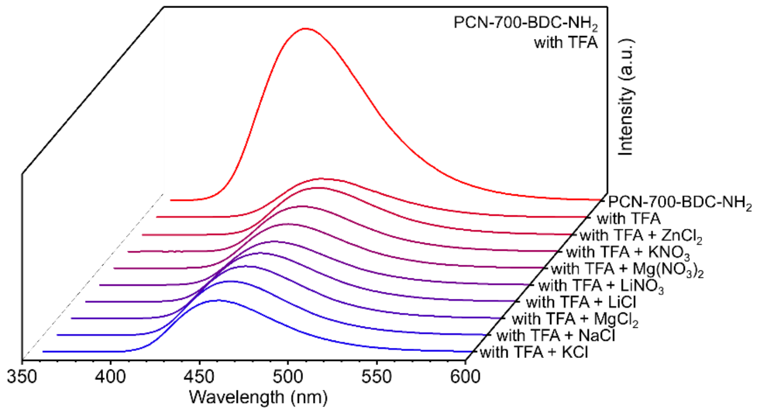

Figure S100. Emission spectra of PCN-700-BDC-NH$_2$ with TFA and 10 mM interfering species.

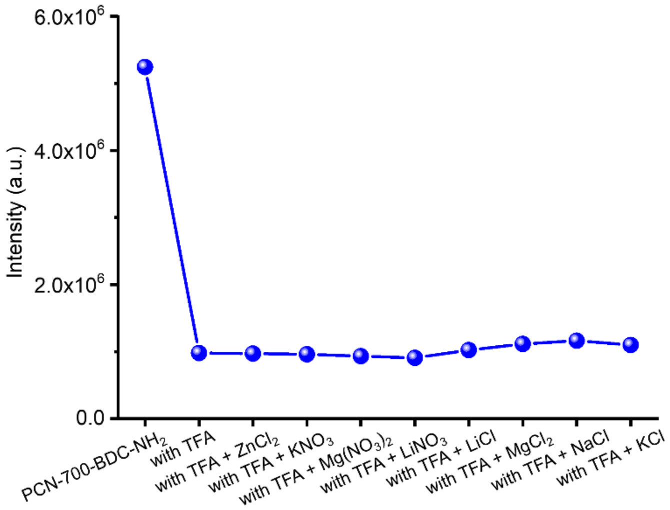

Figure S101. Intensity changes of PCN-700-BDC-NH$_2$ with TFA and 10 mM interfering species.

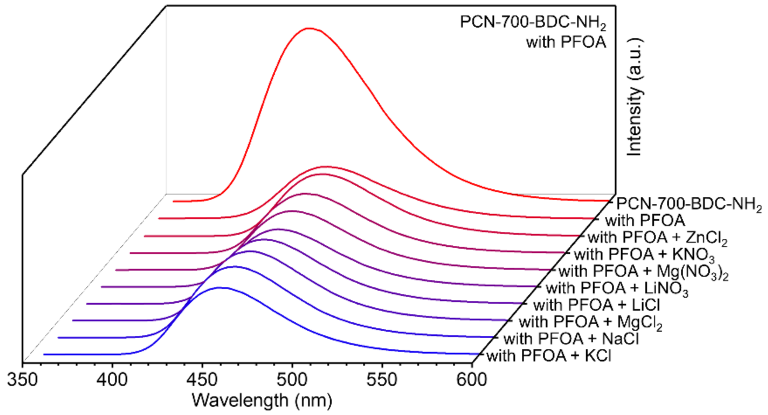

Figure S102. Emission spectra of PCN-700-BDC-NH2 with PFOA and 10 mM interfering species.

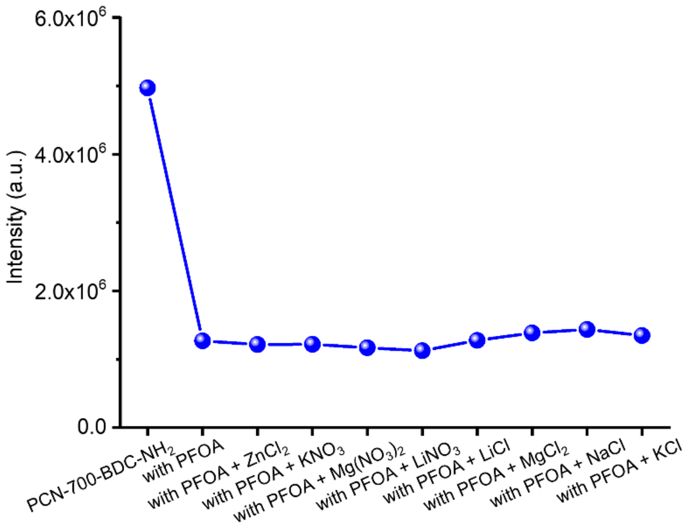

Figure S103. Intensity changes of PCN-700-BDC-NH2 with PFOA and 10 mM interfering species.

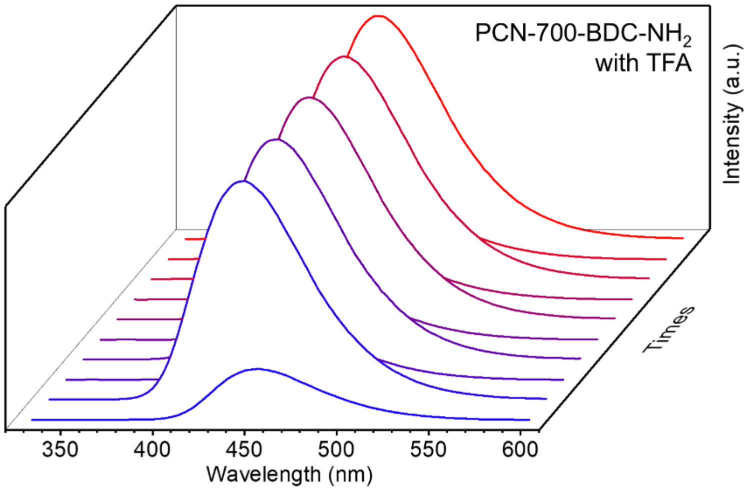

Figure S104. Emission spectra of PCN-700-BDC-NH_2 with TFA for 5 cycles.

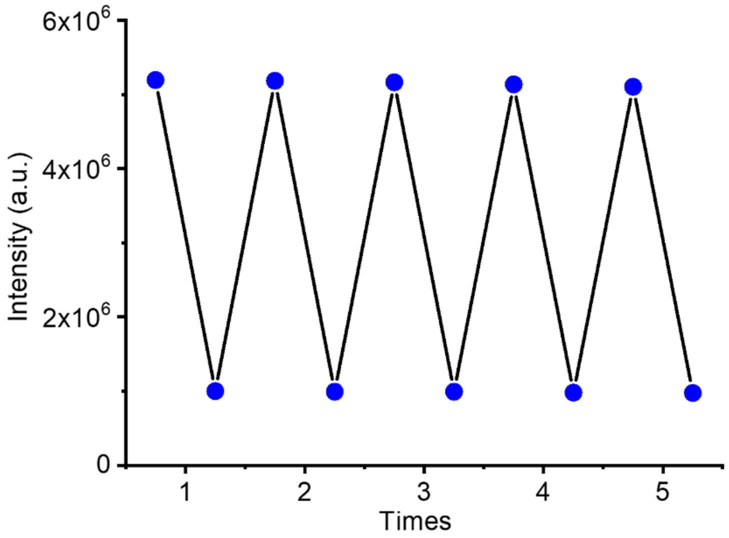

Figure S105. Intensity changes of PCN-700-BDC-NH_2 with TFA for 5 cycles.

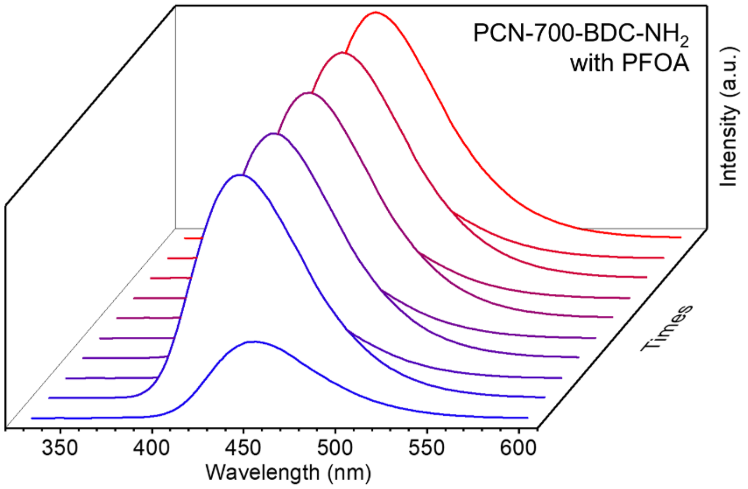

Figure S106. Emission spectra of PCN-700-BDC-NH2 with PFOA for 5 cycles.

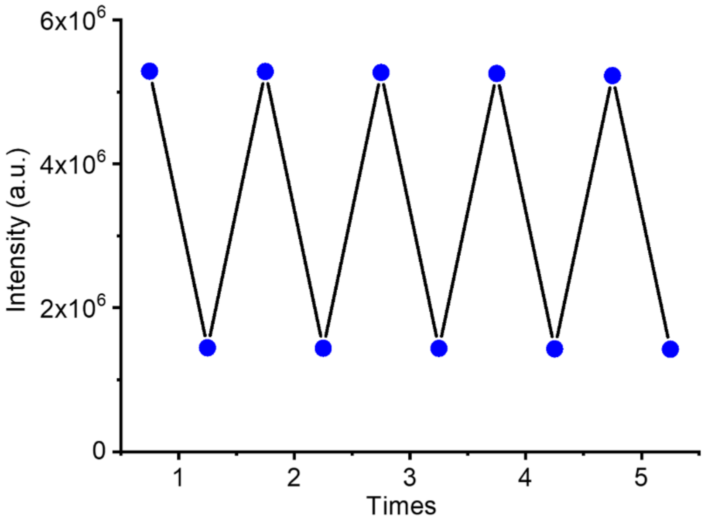

Figure S107. Intensity changes of PCN-700-BDC-NH2 with TFA for 5 cycles.

Sensing Mechanisum

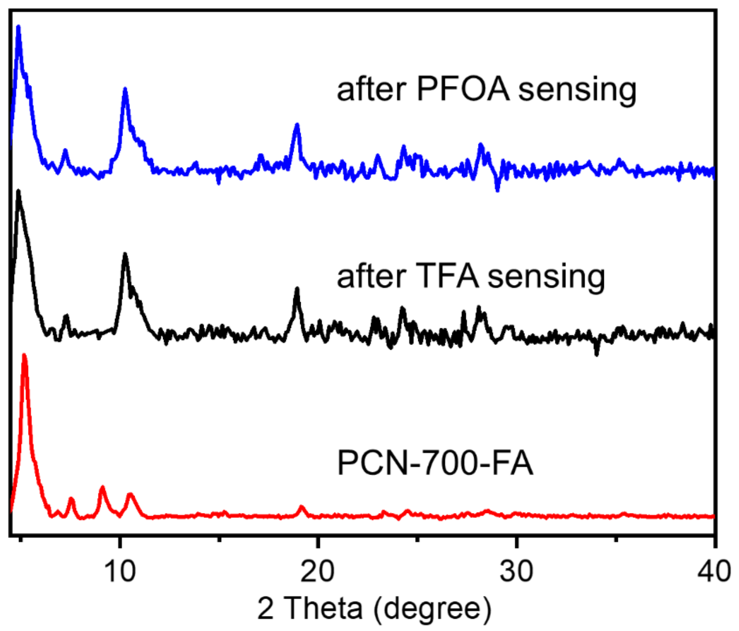

Figure S108. PXRD patterns of PCN-700-FA before and after sensing.

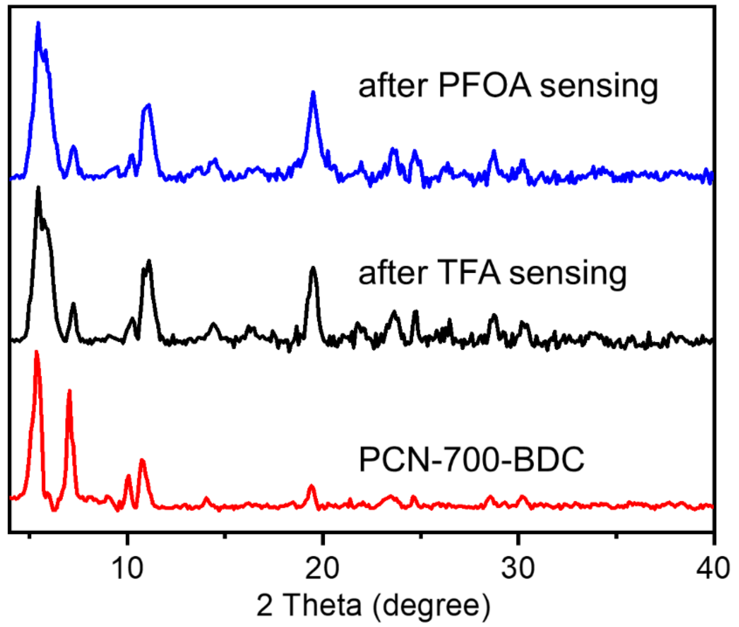

Figure S109. PXRD patterns of PCN-700-BDC before and after sensing.

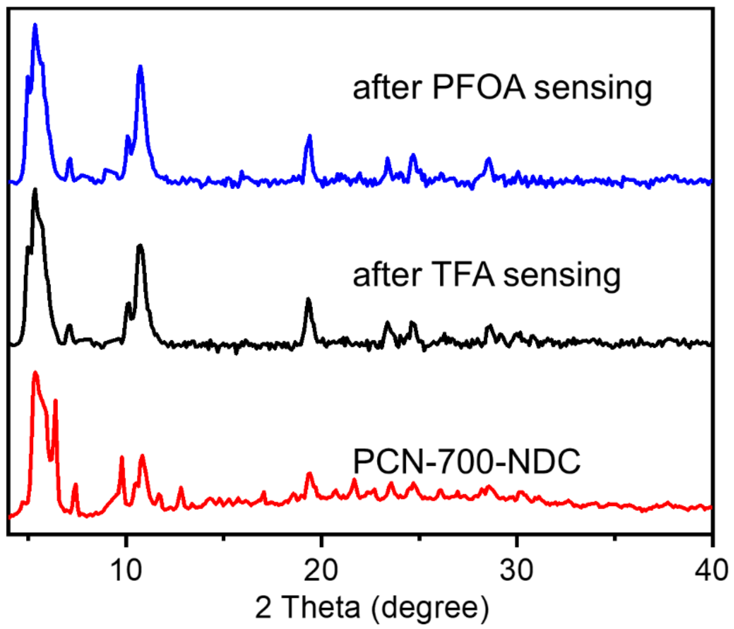

Figure S110. PXRD patterns of PCN-700-NDC before and after sensing.

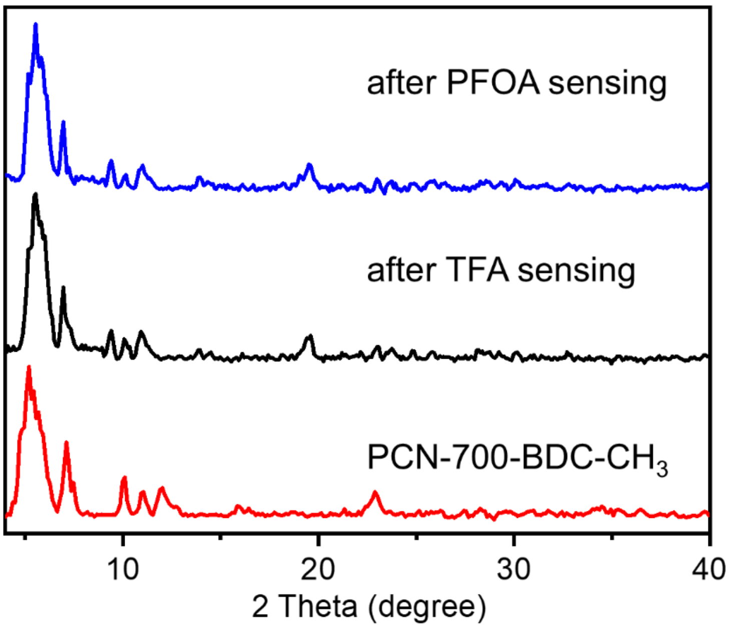

Figure S111. PXRD patterns of PCN-700-BDC-CH_3 before and after sensing.

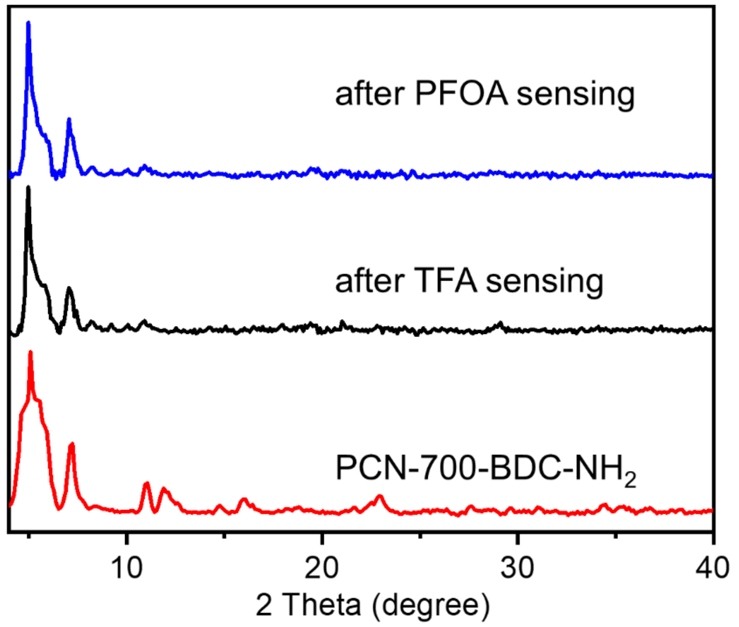

Figure S112. PXRD patterns of PCN-700-BDC-NH_2 before and after sensing.

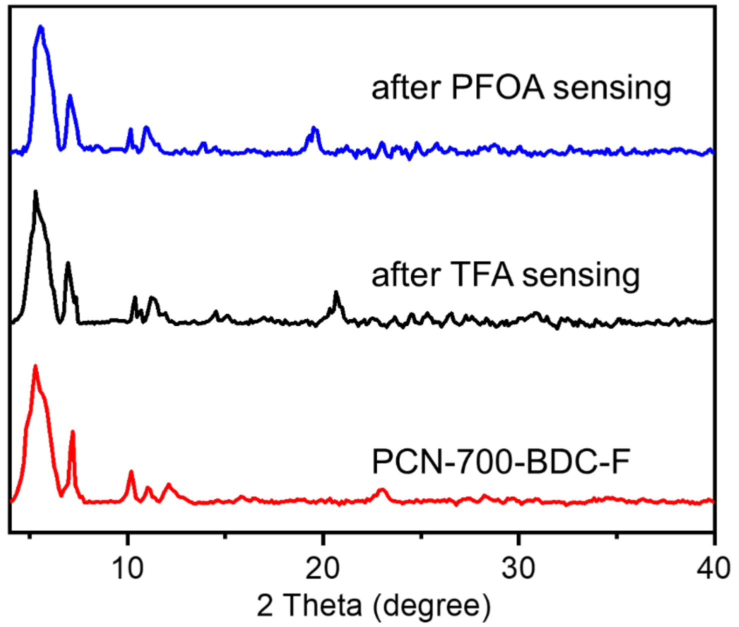

Figure S113. PXRD patterns of PCN-700-BDC-F before and after sensing.

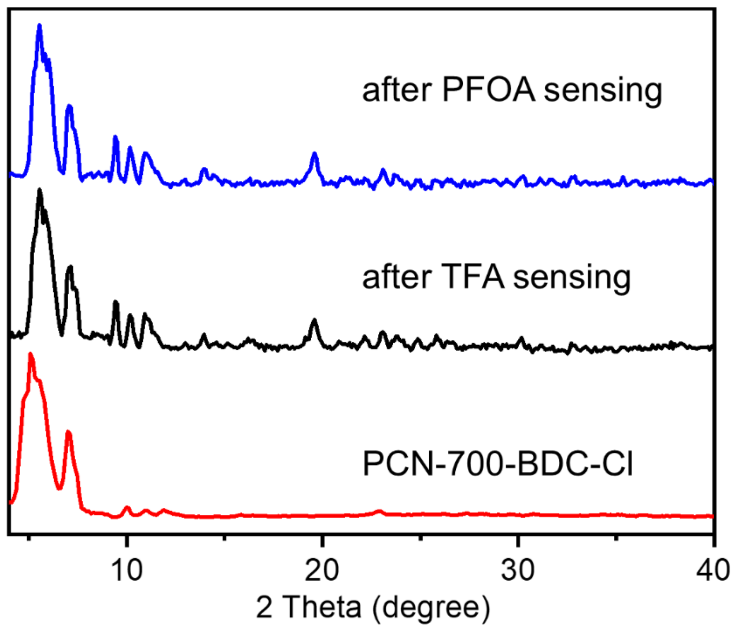

Figure S114. PXRD patterns of PCN-700-BDC-Cl before and after sensing.

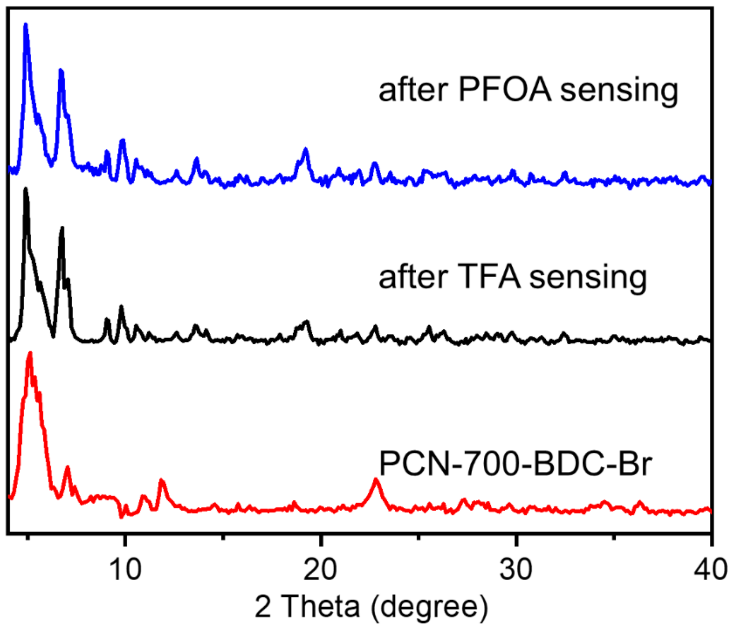

Figure S115. PXRD patterns of PCN-700-BDC-Br before and after sensing.

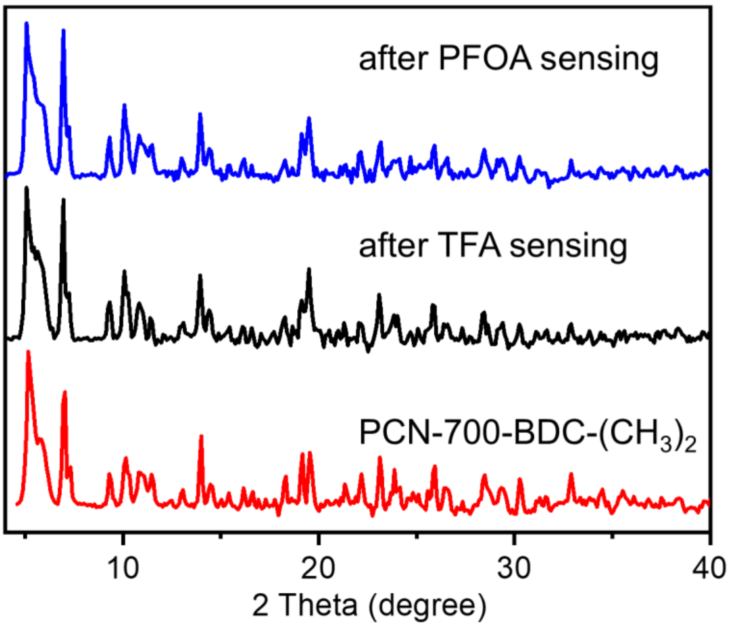

Figure S116. PXRD patterns of PCN-700-BDC-$(CH_3)_2$ before and after sensing.

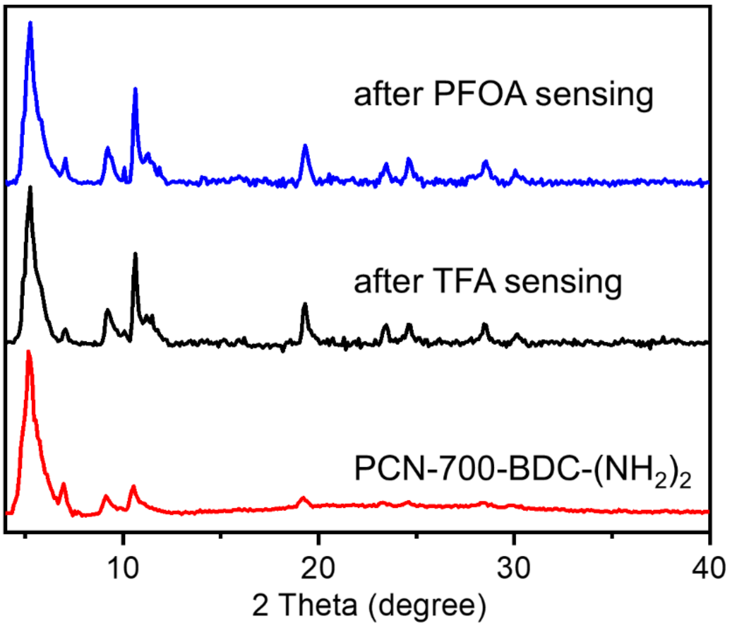

Figure S117. PXRD patterns of PCN-700-BDC-$(NH_2)_2$ before and after sensing.

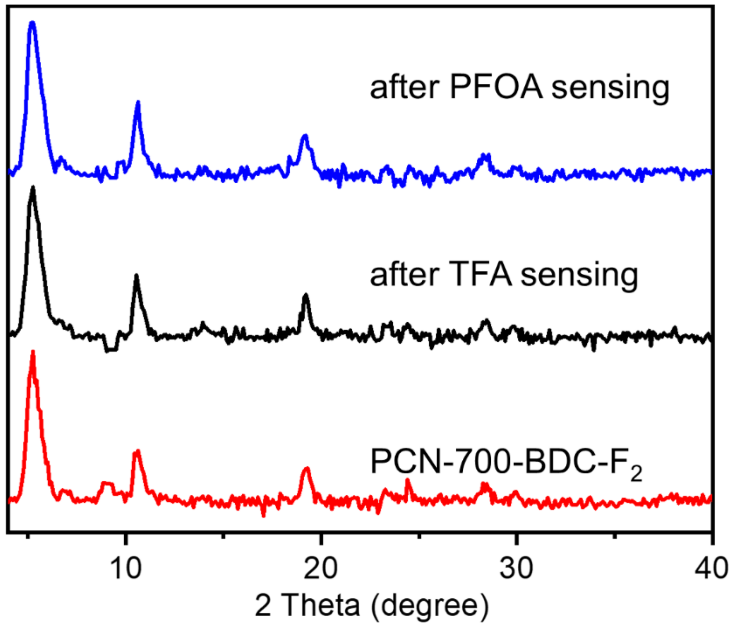

Figure S118. PXRD patterns of PCN-700-BDC-F_2 before and after sensing.

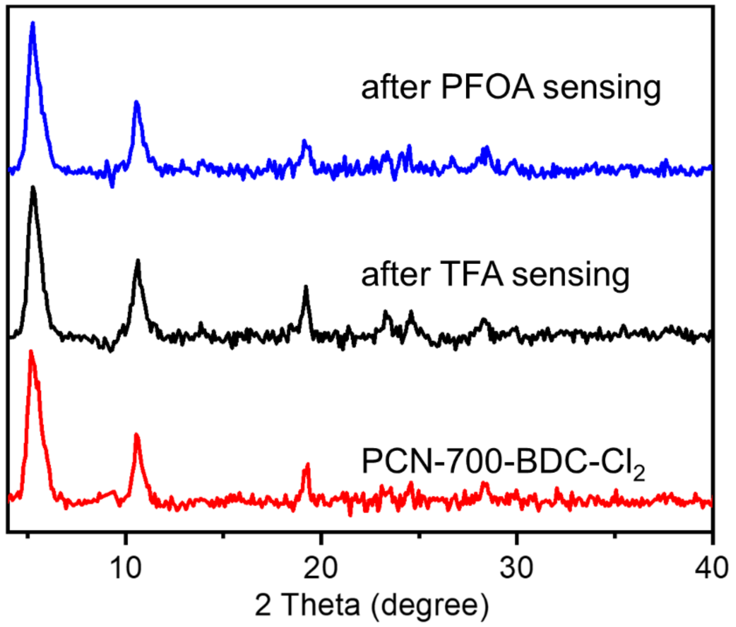

Figure S119. PXRD patterns of PCN-700-BDC-Cl_2 before and after sensing.

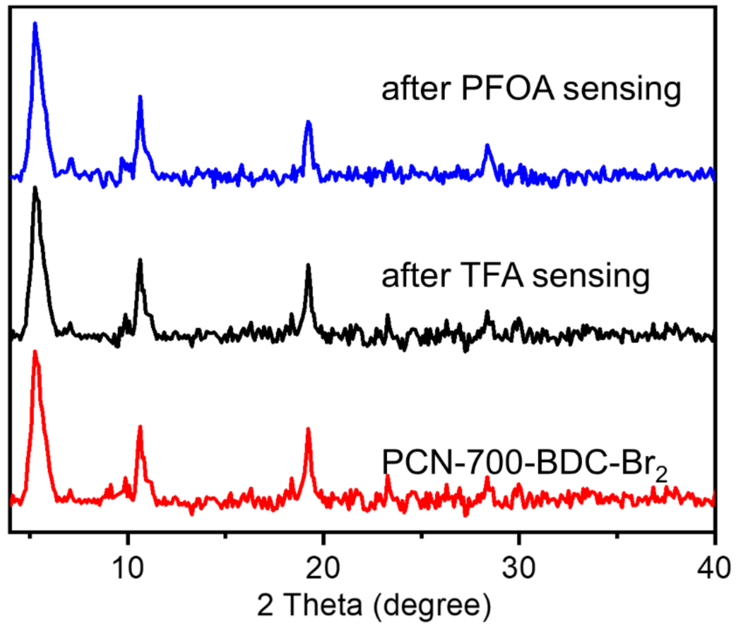

Figure S120. PXRD patterns of PCN-700-BDC-Br_2 before and after sensing.

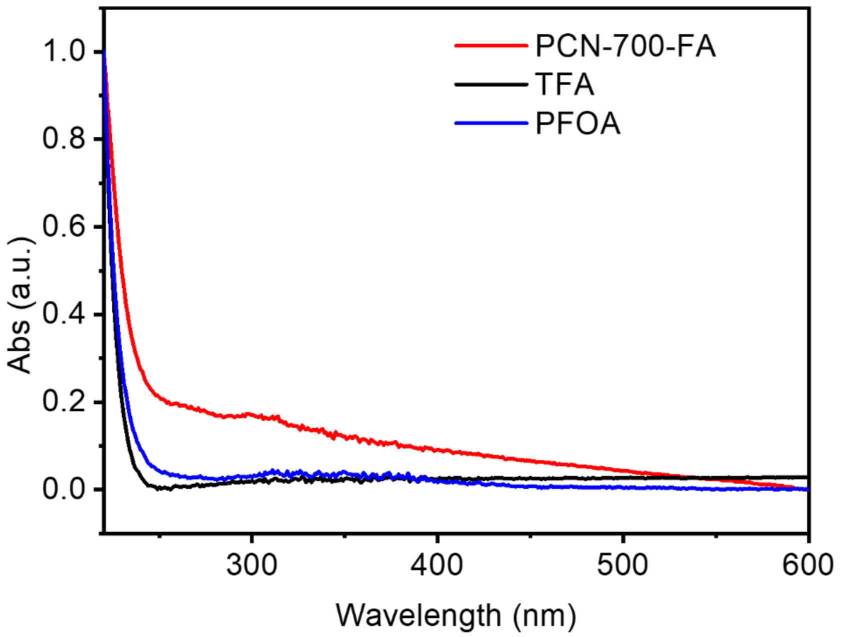

Figure S121. UV-vis spectra of PCN-700-FA, TFA and PFOA.

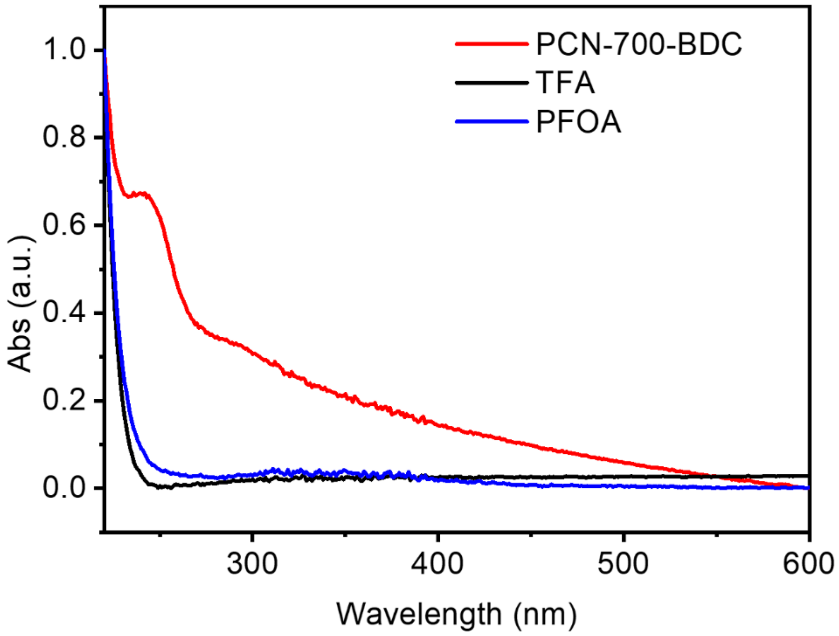

Figure S122. UV-vis spectra of PCN-700-BDC, TFA and PFOA.

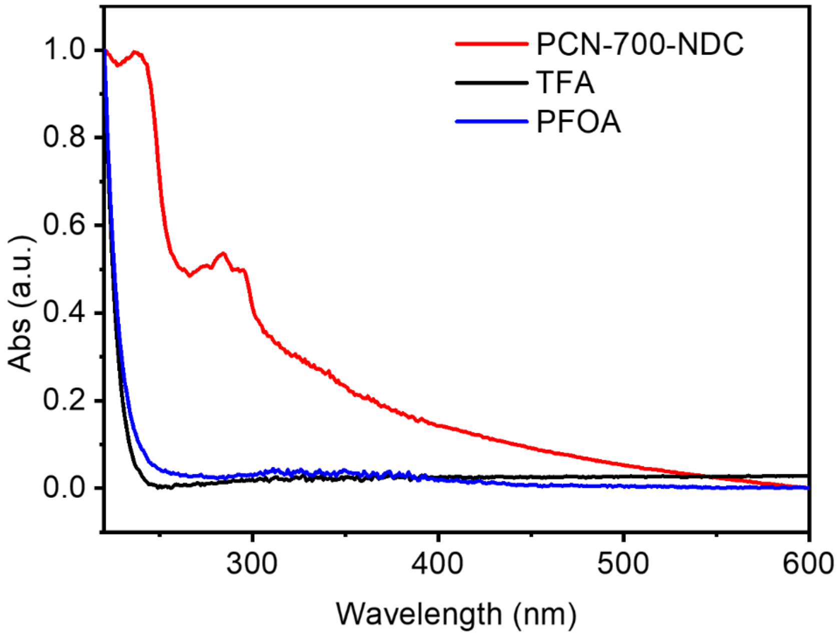

Figure S123. UV-vis spectra of PCN-700-NDC, TFA and PFOA.

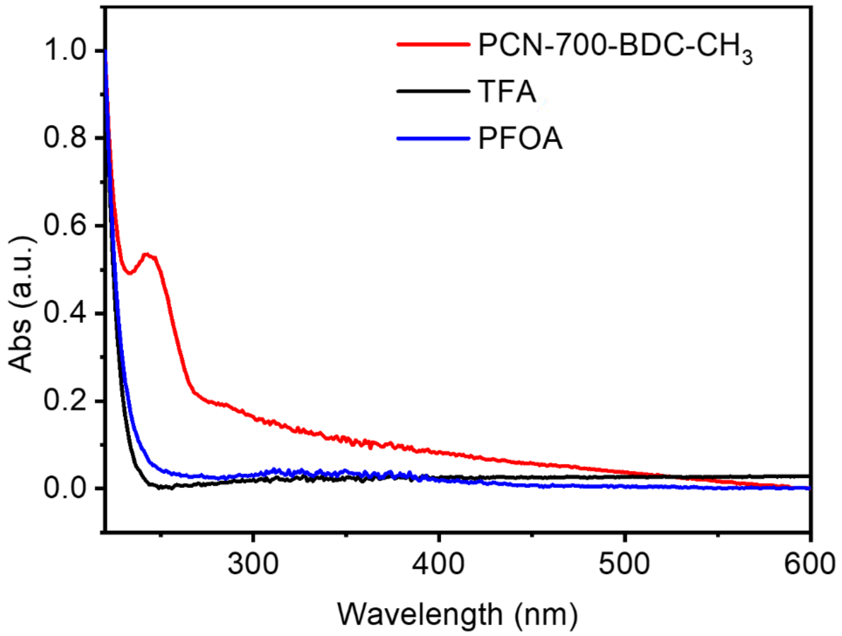

Figure S124. UV-vis spectra of PCN-700-BDC-CH_3, TFA and PFOA.

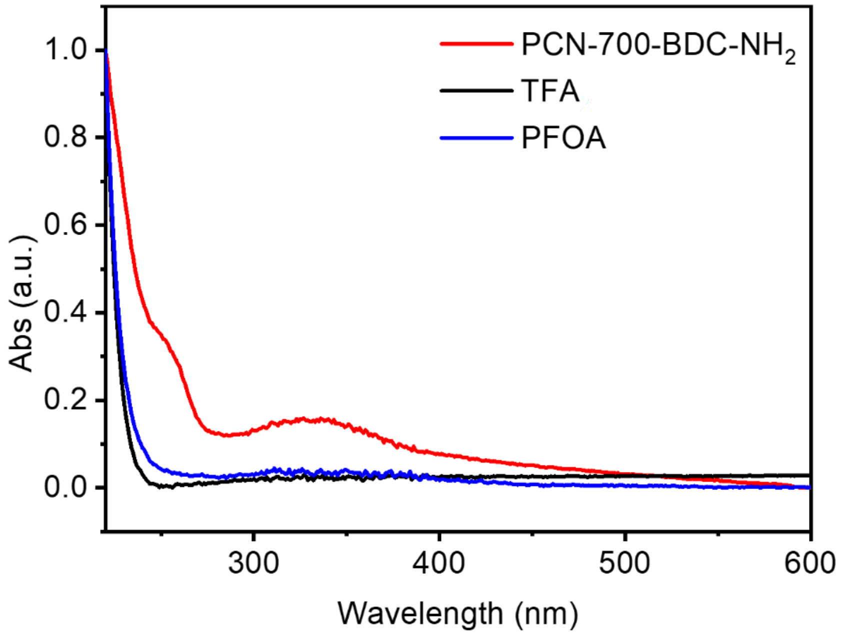

Figure S125. UV-vis spectra of PCN-700-BDC-NH_2, TFA and PFOA.

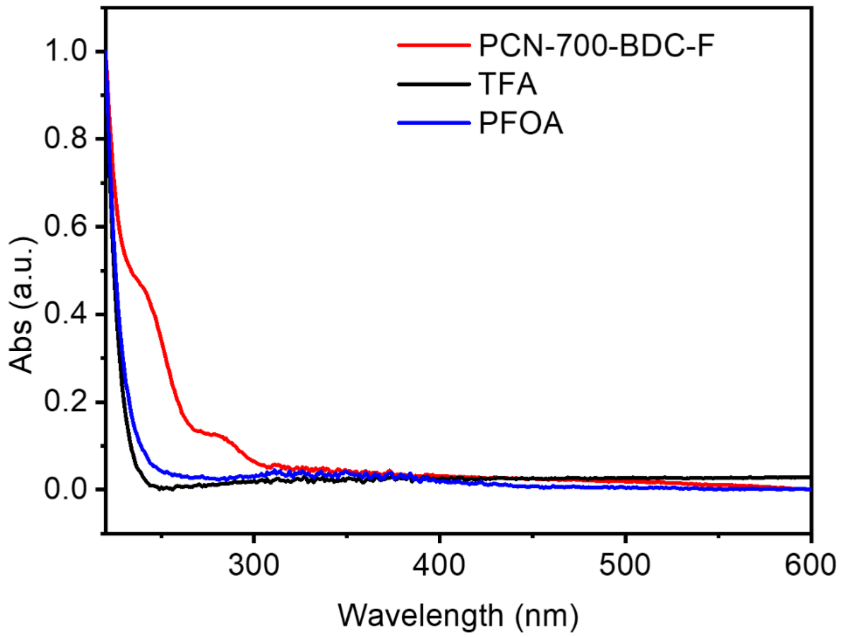

Figure S126. UV-vis spectra of PCN-700-BDC-F, TFA and PFOA.

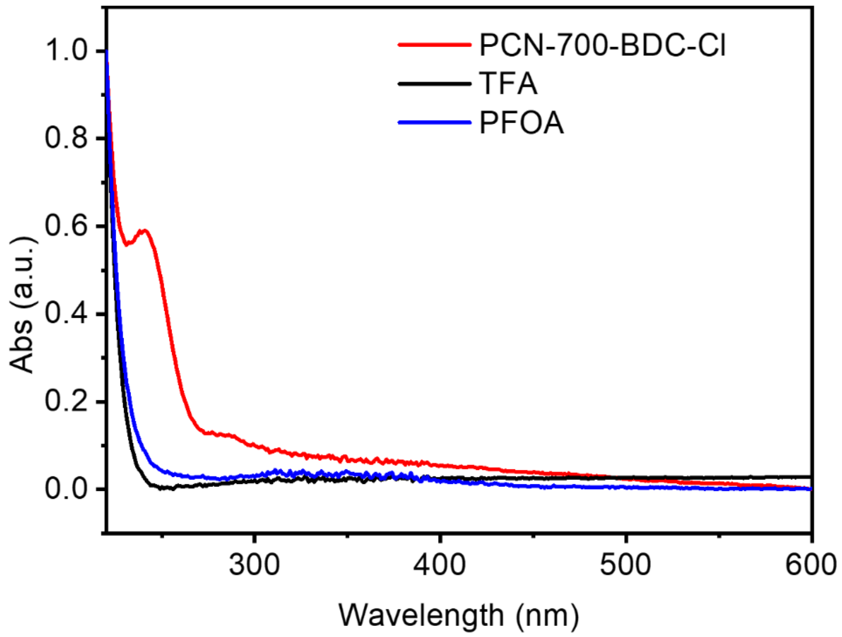

Figure S127. UV-vis spectra of PCN-700-BDC-Cl, TFA and PFOA.

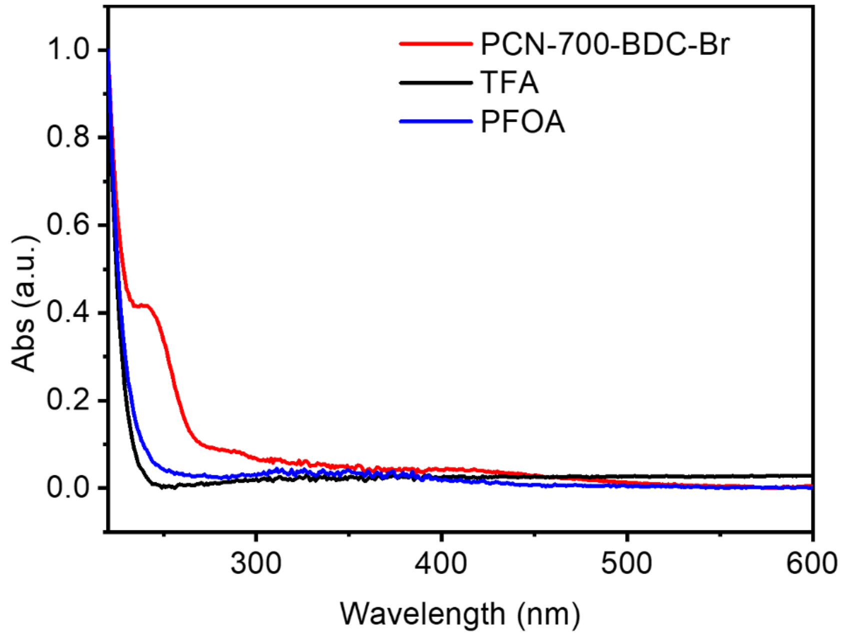

Figure S128. UV-vis spectra of PCN-700-BDC-Br, TFA and PFOA.

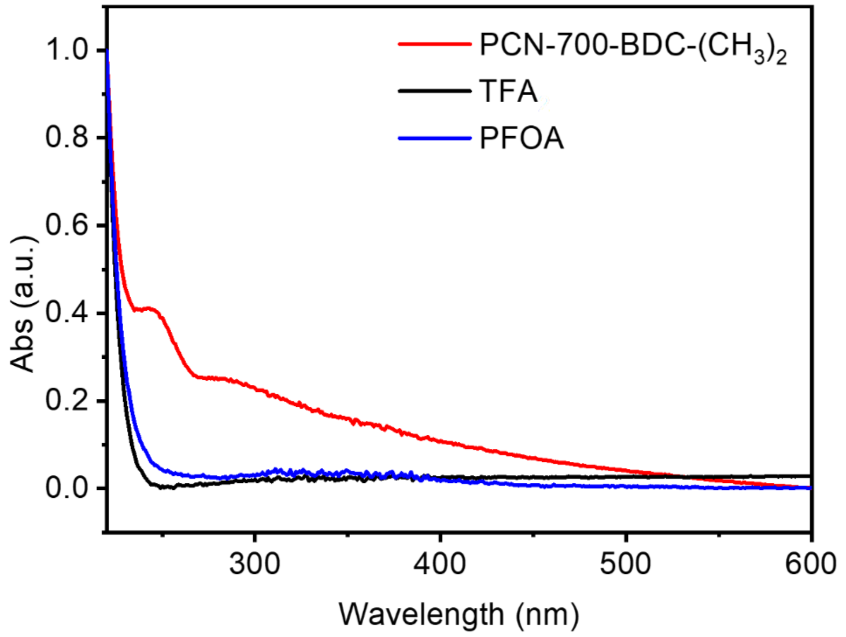

Figure S129. UV-vis spectra of PCN-700-BDC-$(CH_3)_2$, TFA and PFOA.

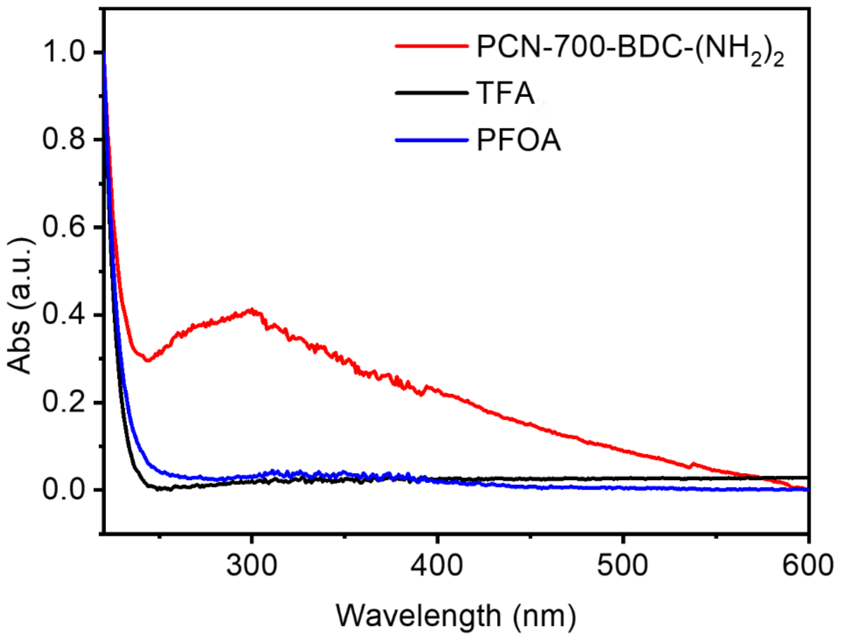

Figure S130. UV-vis spectra of PCN-700-BDC-$(NH_2)_2$, TFA and PFOA.

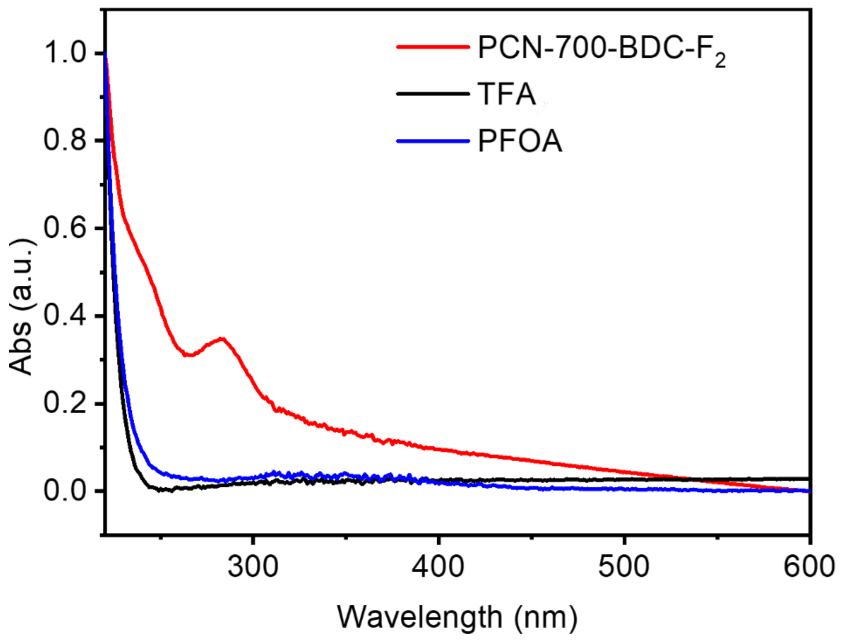

Figure S131. UV-vis spectra of PCN-700-BDC-F_2, TFA and PFOA.

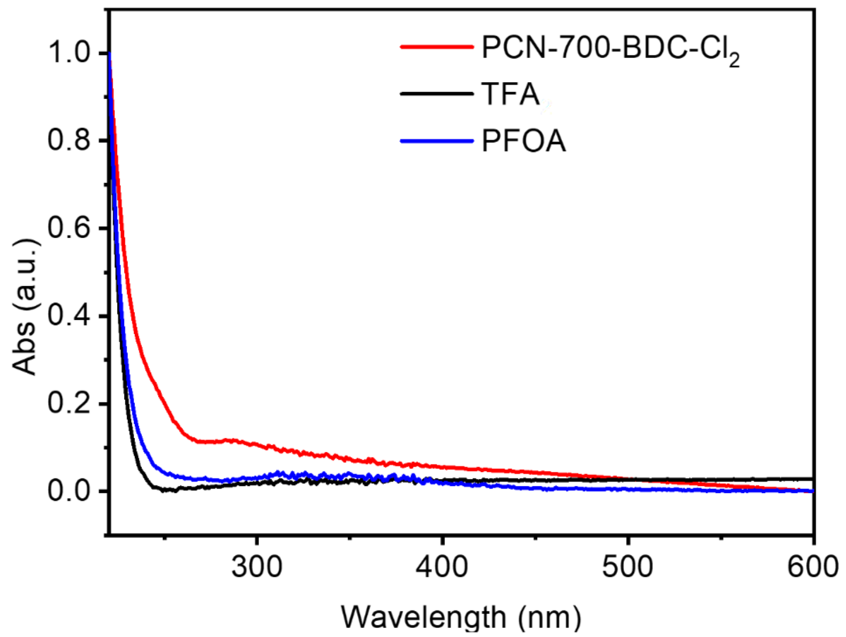

Figure S132. UV-vis spectra of PCN-700-BDC-Cl_2, TFA and PFOA.

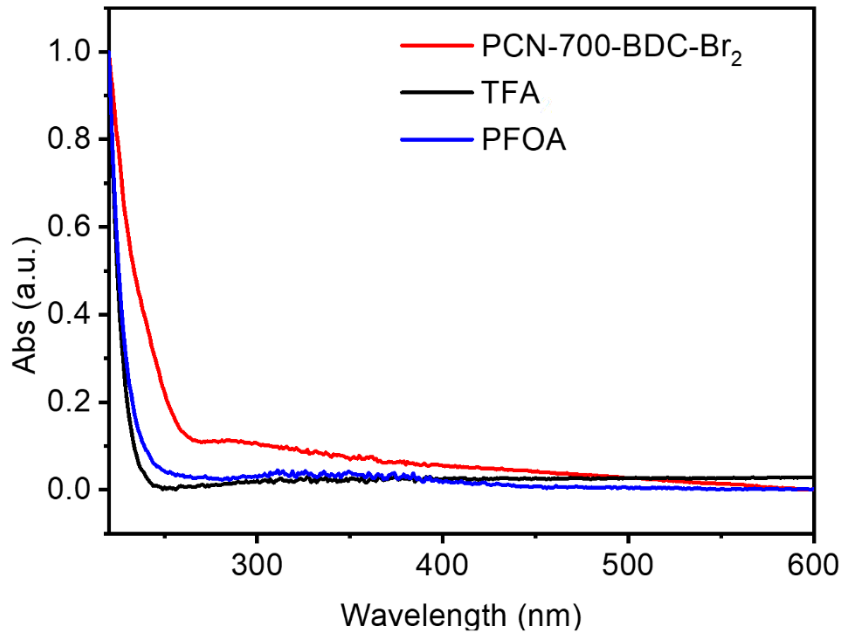

Figure S133. UV-vis spectra of PCN-700-BDC-Br_2, TFA and PFOA.

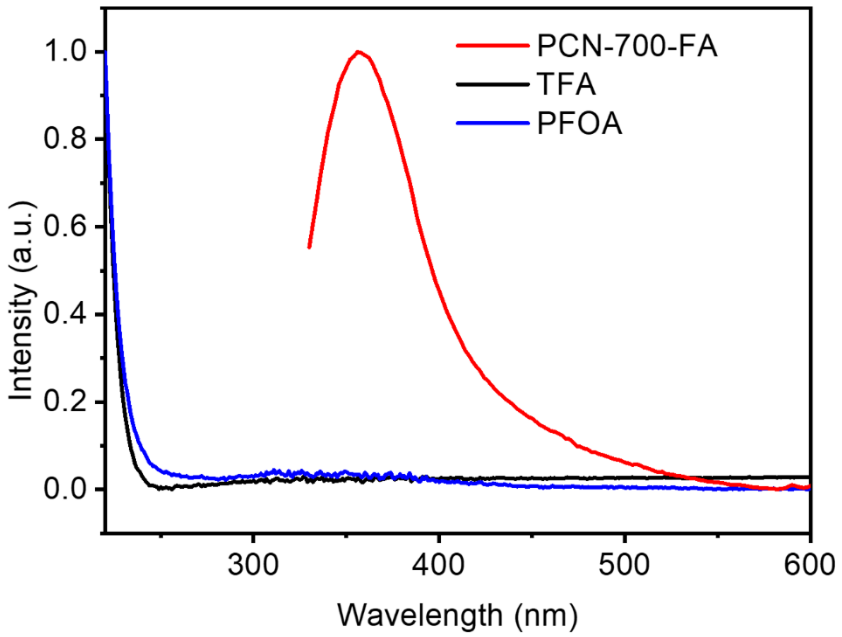

Figure S134. Emission spectrum of PCN-700-FA and UV-vis spectra of TFA and PFOA.

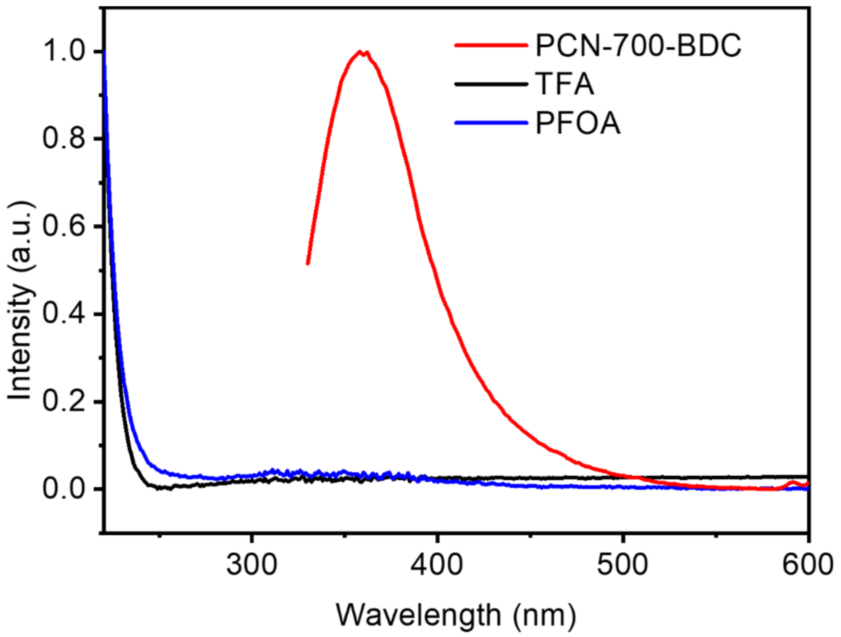

Figure S135. Emission spectrum of PCN-700-BDC and UV-vis spectra of TFA and PFOA.

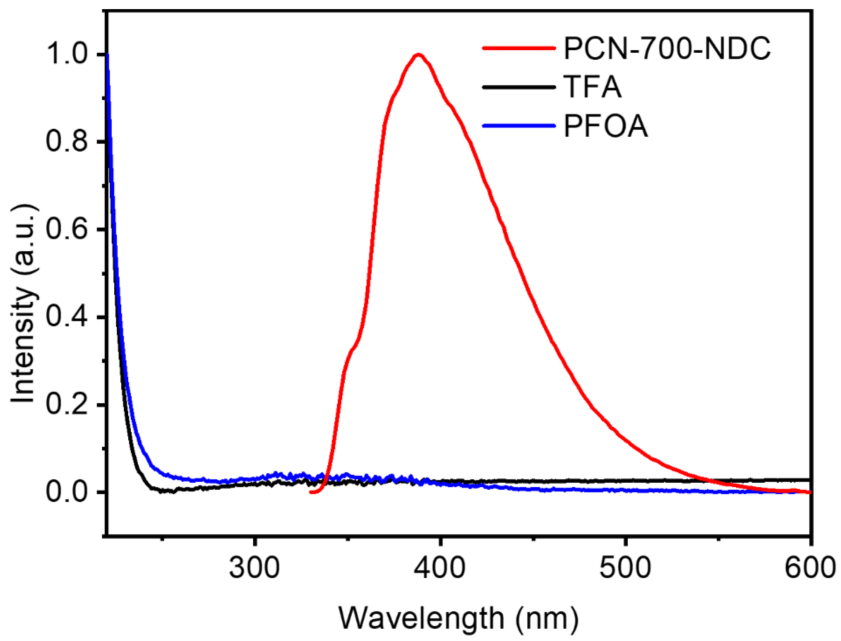

Figure S136. Emission spectrum of PCN-700-NDC and UV-vis spectra of TFA and PFOA.

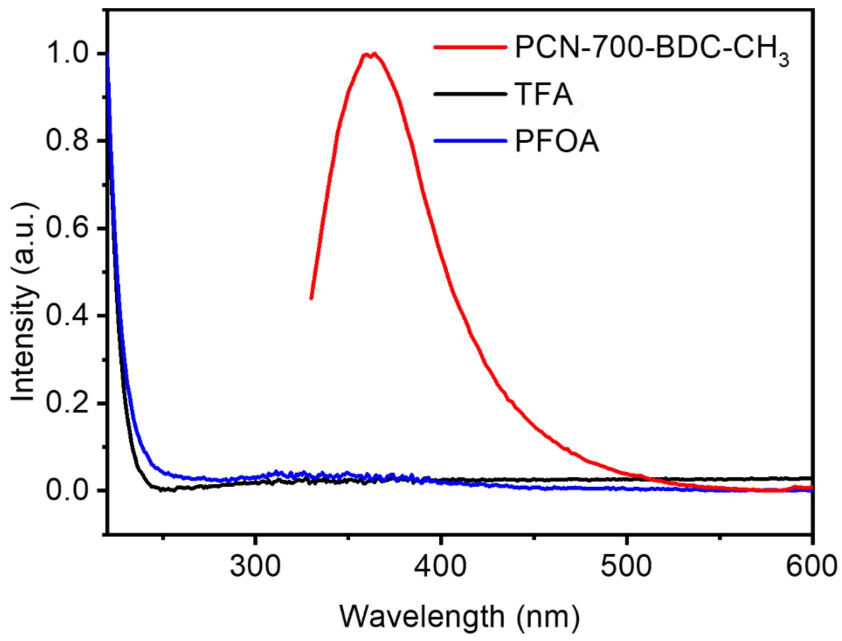

Figure S137. Emission spectrum of PCN-700-BDC-CH_3 and UV-vis spectra of TFA and PFOA.

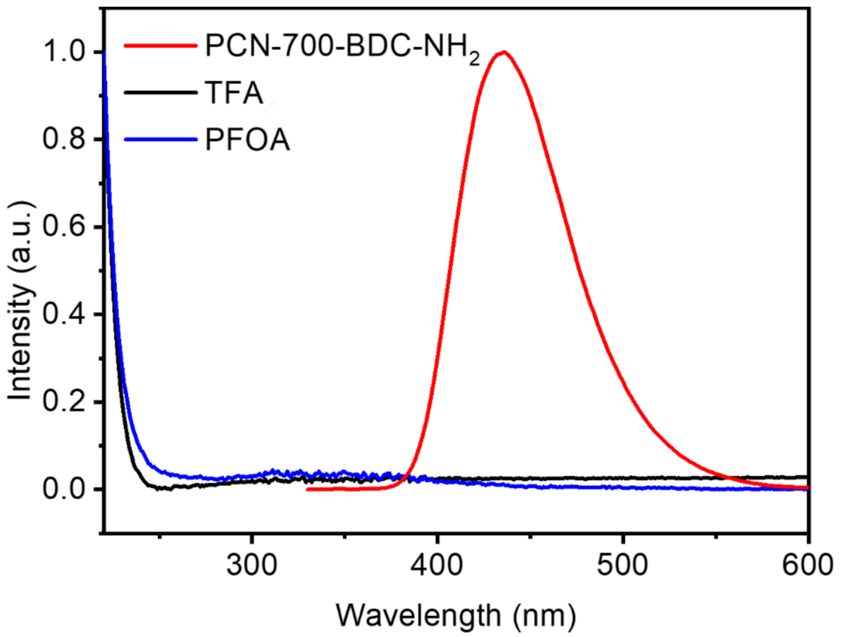

Figure S138. Emission spectrum of PCN-700-BDC-NH_2 and UV-vis spectra of TFA and PFOA.

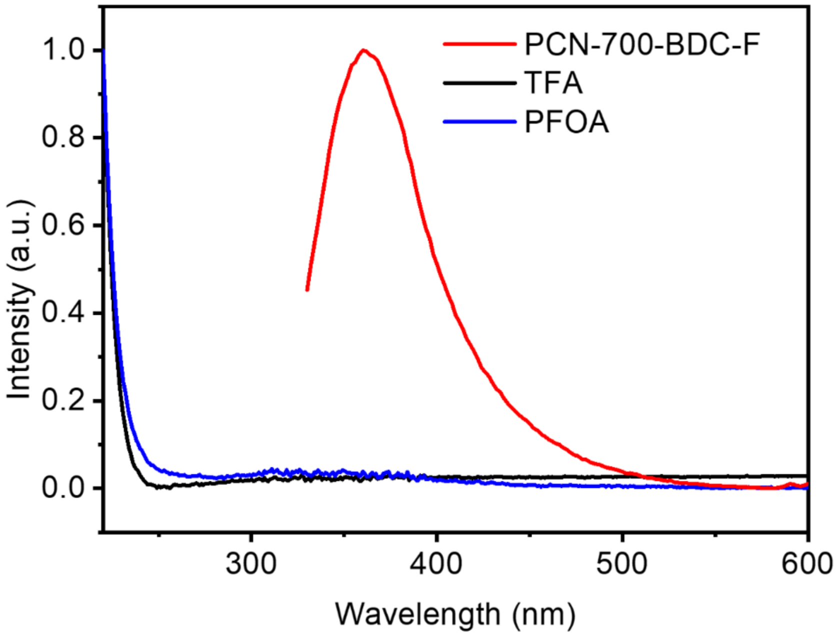

Figure S139. Emission spectrum of PCN-700-BDC-F and UV-vis spectra of TFA and PFOA.

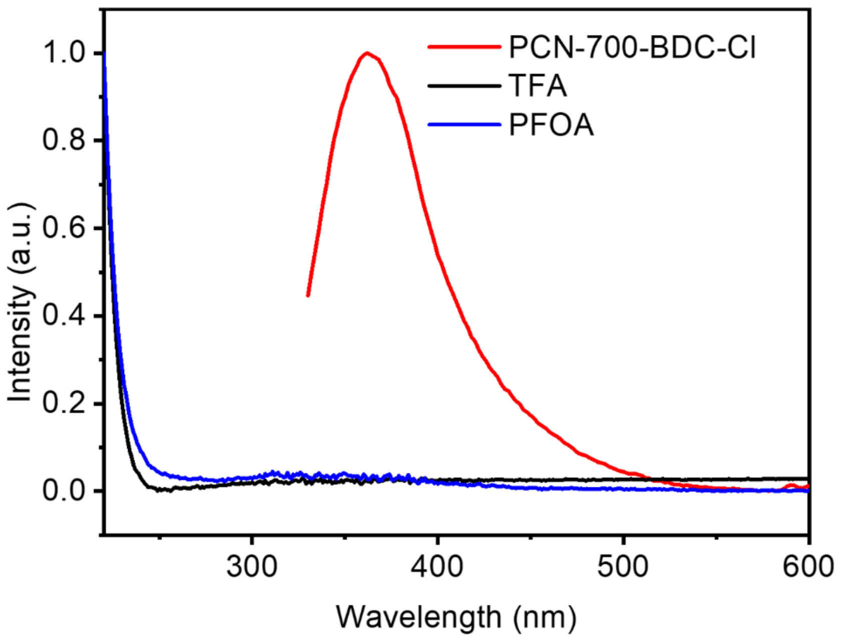

Figure S140. Emission spectrum of PCN-700-BDC-Cl and UV-vis spectra of TFA and PFOA.

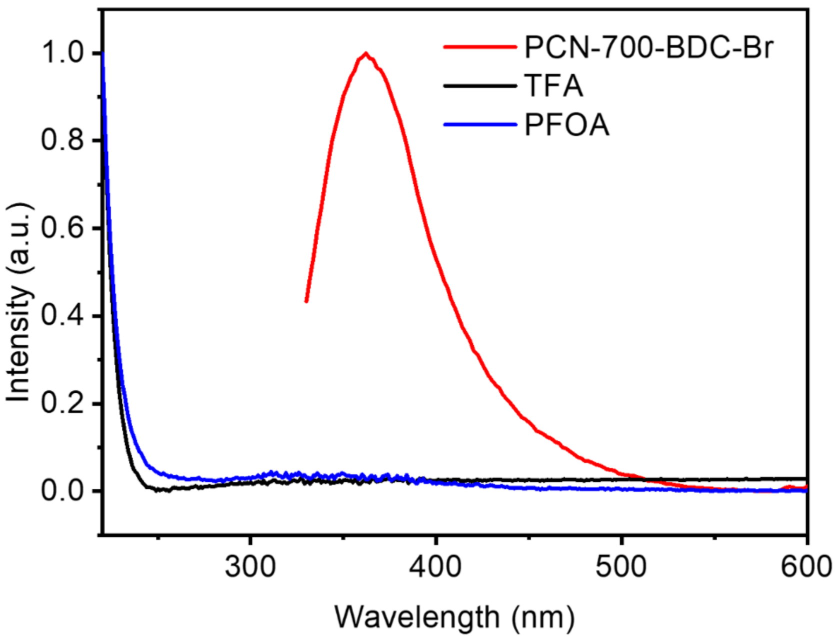

Figure S141. Emission spectrum of PCN-700-BDC-Br and UV-vis spectra of TFA and PFOA.

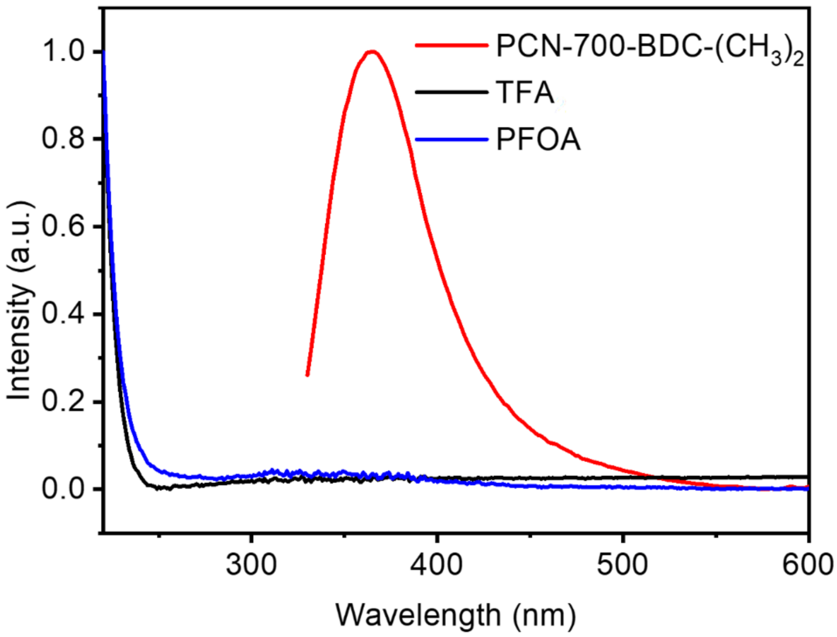

Figure S142. Emission spectrum of PCN-700-BDC-$(CH_3)_2$ and UV-vis spectra of TFA and PFOA.

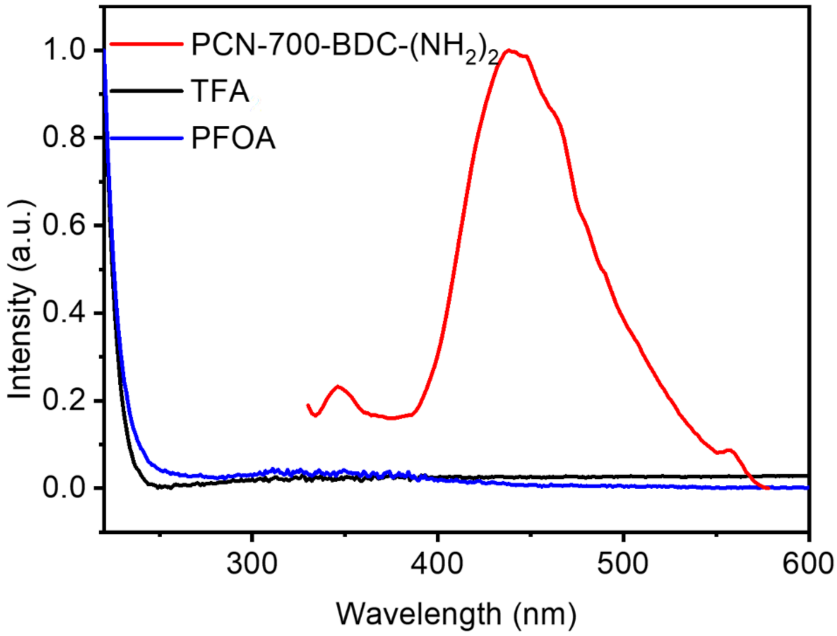

Figure S143. Emission spectrum of PCN-700-BDC-$(NH_2)_2$ and UV-vis spectra of TFA and PFOA.

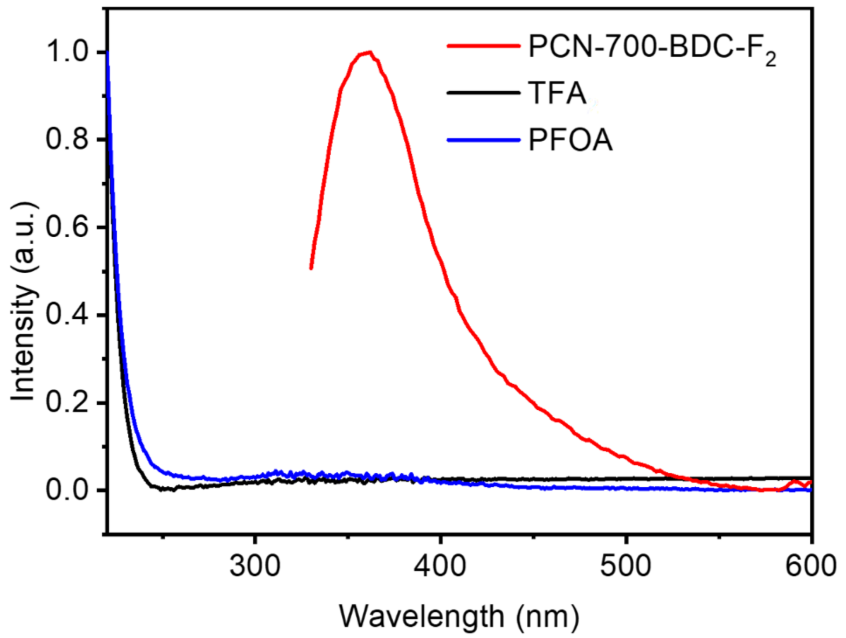

Figure S144. Emission spectrum of PCN-700-BDC-F_2 and UV-vis spectra of TFA and PFOA.

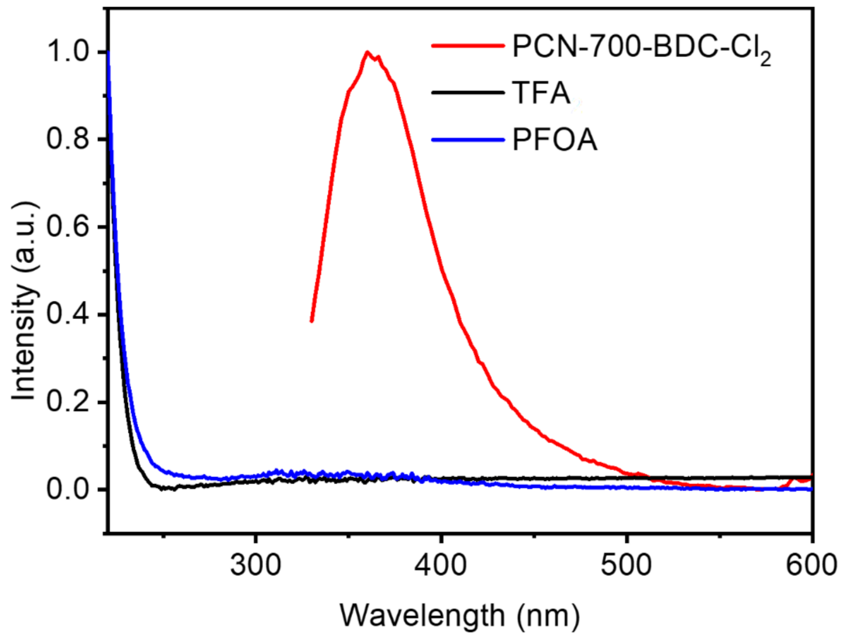

Figure S145. Emission spectrum of PCN-700-BDC-Cl_2 and UV-vis spectra of TFA and PFOA.

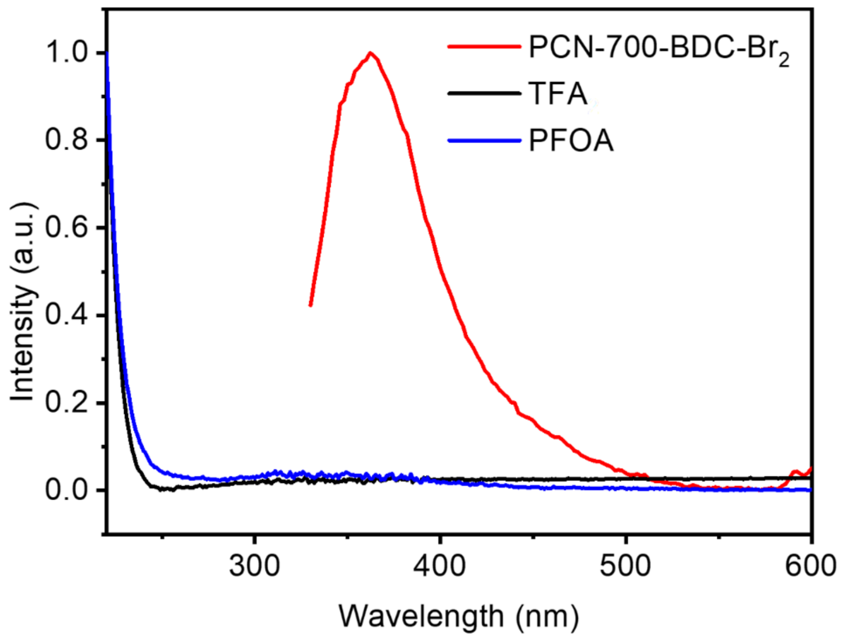

Figure S146. Emission spectrum of PCN-700-BDC-Br2 and UV-vis spectra of TFA and PFOA.

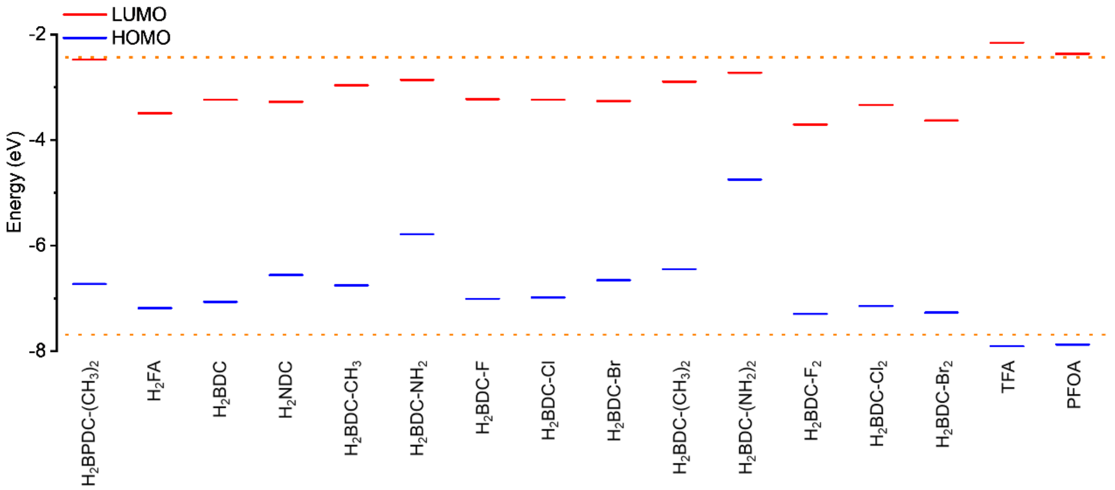

Figure S147. HOMO and LUMO energy levels of the linkers and analytes.

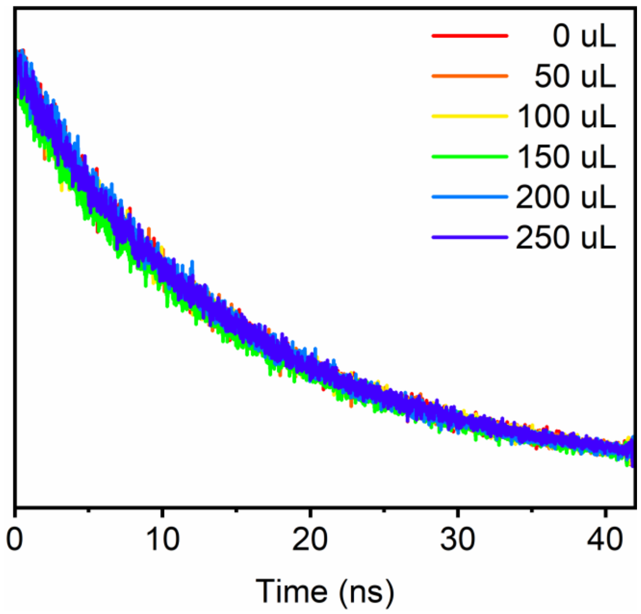

Figure S148. Lifetime tests of PCN-700-BDC-NH_2 with the additions of TFA (1 uL/mL).

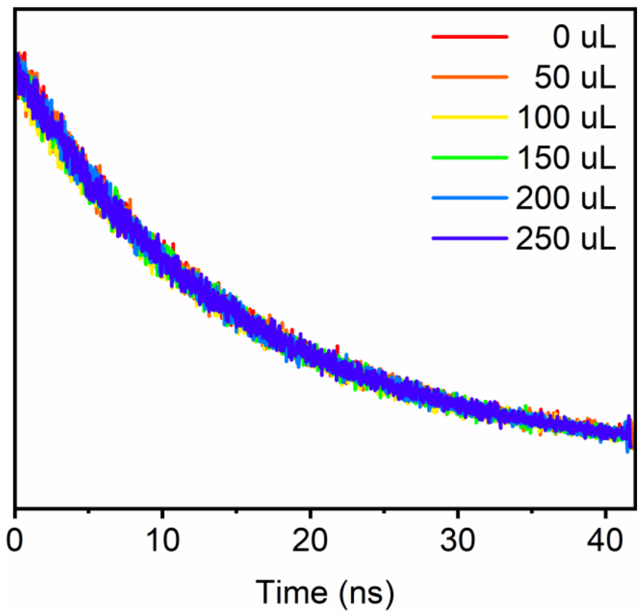

Figure S149. Lifetime tests of PCN-700-BDC-NH_2 with the additions of PFOA (1 mg/mL).

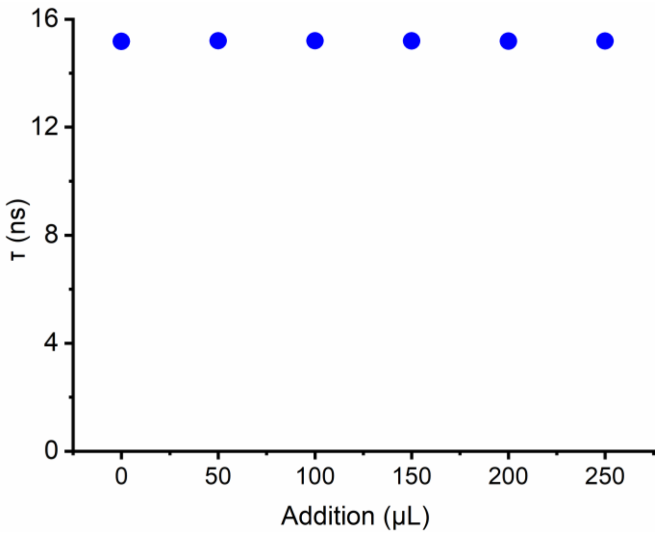

Figure S150. Lifetime changes of PCN-700-BDC-NH_2 with the additions of TFA (1 uL/mL).

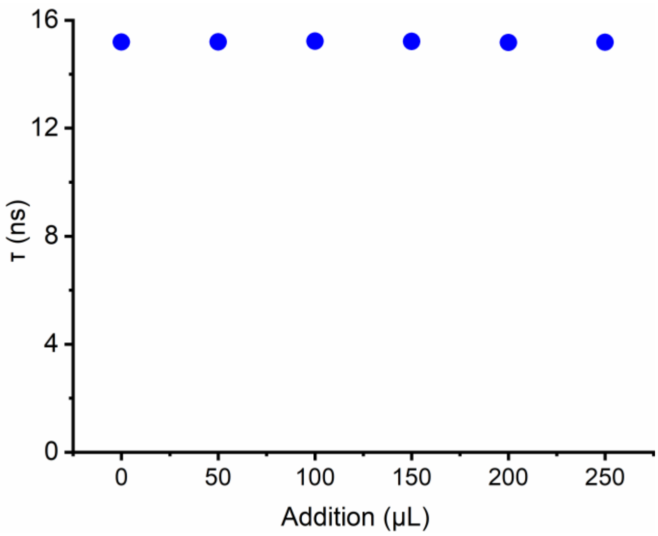

Figure S151. Lifetime changes of PCN-700-BDC-NH_2 with the additions of PFOA (1 mg/mL).

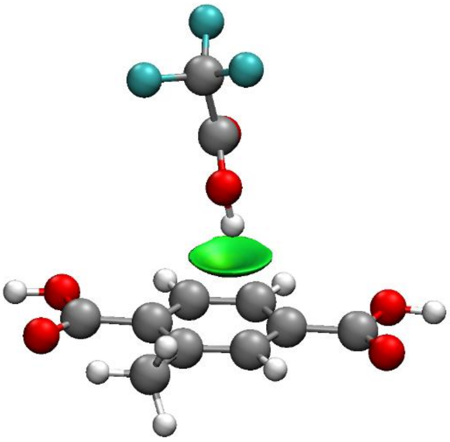

Figure S152. Interactions between $H_2BDC-CH_3$ and TFA. Atom code: C, grey; O, red; H, white; F, cyan.

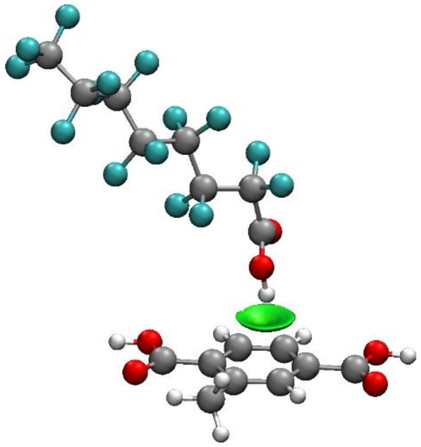

Figure S153. Interactions between $H_2BDC-CH_3$ and PFOA. Atom code: C, grey; O, red; H, white; F, cyan.

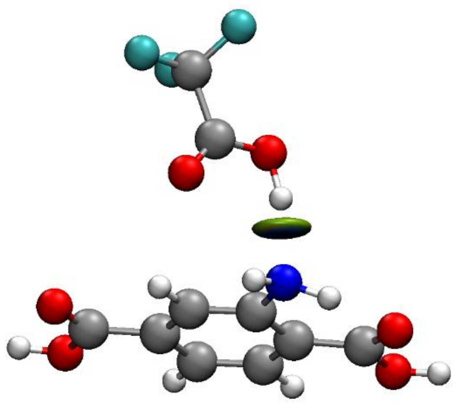

Figure S154. Interactions between $H_2BDC-NH_2$ and TFA. Atom code: C, grey; O, red; H, white; N, blue; F, cyan.

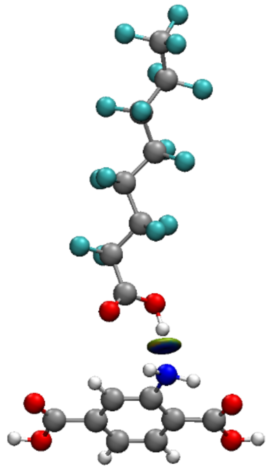

Figure S155. Interactions between $H_2BDC-NH_2$ and PFOA. Atom code: C, grey; O, red; H, white; N, blue; F, cyan.

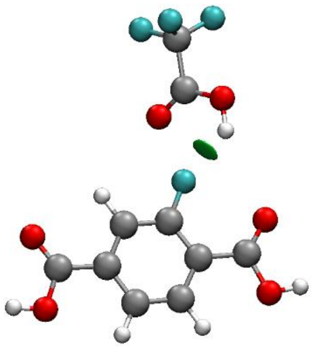

Figure S156. Interactions between H_2BDC-F and TFA. Atom code: C, grey; O, red; H, white; F, cyan.

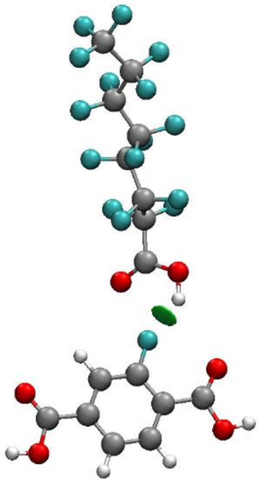

Figure S157. Interactions between H_2BDC-F and PFOA. Atom code: C, grey; O, red; H, white; F, cyan.

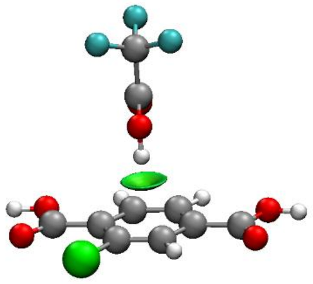

Figure S158. Interactions between H_2BDC-Cl and TFA. Atom code: C, grey; O, red; H, white; F, cyan; Cl, green.

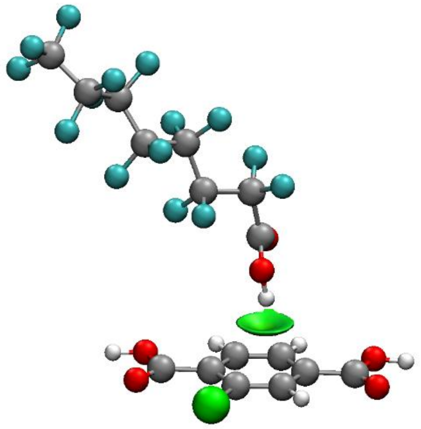

Figure S159. Interactions between H_2BDC-Cl and PFOA. Atom code: C, grey; O, red; H, white; F, cyan; Cl, green.

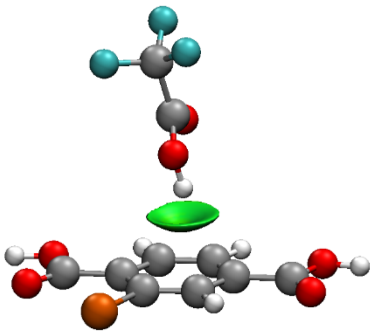

Figure S160. Interactions between H_2BDC-Br and TFA. Atom code: C, grey; O, red; H, white; F, cyan; Br, brown.

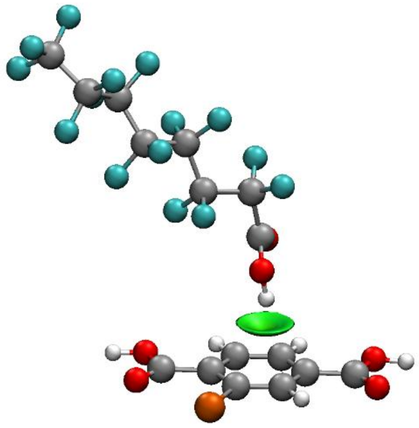

Figure S161. Interactions between H_2BDC-Br and PFOA. Atom code: C, grey; O, red; H, white; F, cyan; Br, brown.

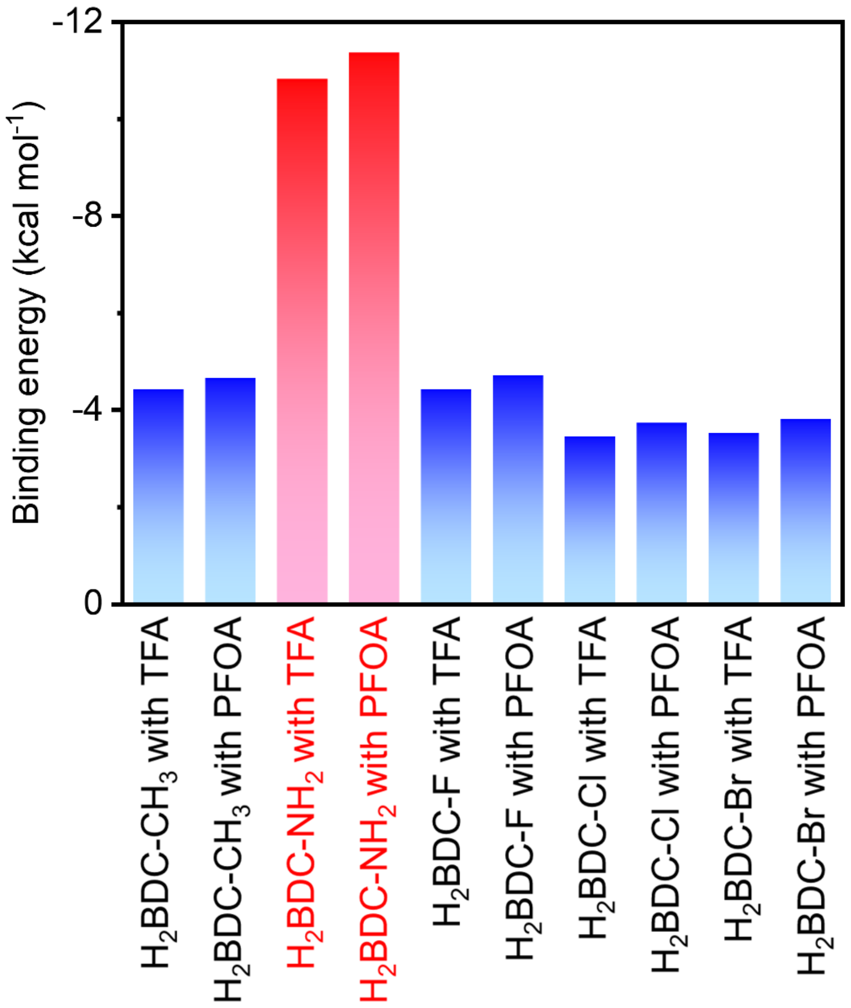

Figure S162. Binding energies between the ligands and TFA/PFOA.

Tables

Table S1. Crystallographic data and structure refinement details.

	**PCN-700-FA**	**PCN-700-BDC**	**PCN-700-NDC**
CCDC	1496835	1036875	1496837
Formula	$C_{68}H_{50}O_{32}Zr_6$	$C_{72}H_{58}O_{32}Zr_6$	$C_{76}H_{54}O_{32}Zr_6$
Formula wt	1926.40	1982.50	2026.51
Temperature (K)	110	110	110
Crystal System	tetragonal	tetragonal	tetragonal
Space Group	$P4_2/ncm$	$P4_2/mmc$	$P4_2/mmc$
a (Å)	35.053(17)	24.40(3)	23.679(8)
b (Å)	35.053(17)	24.40(3)	23.679(8)
c (Å)	12.665(6)	14.88(2)	16.816(6)
α (deg)	90	90	90
β (deg)	90	90	90
γ (deg)	90	90	90
Z	4	2	2
V (Å^3)	15561(11)	8861(16)	9428(4)
ρ_{calc} (g cm^{-3})	0.822	0.743	0.714
μ (mm^{-1})	0.429	0.377	0.356
F (000)	3816.0	1972.0	2012.0
R_{int}	0.2091	0.1359	0.1095
$^aR_1$, $^bwR_2$ ($I > 2\sigma(I)$)	0.0616, 0.1114	0.0565, 0.1271	0.0497, 0.1097
$^aR_1$, $^bwR_2$ (all data)	0.1440, 0.1247	0.0865, 0.1359	0.0728, 0.1154

$^aR_1 = \Sigma||F_o| - |F_c||/\Sigma|F_o|$, $^bwR_2 = [\Sigma w(F_o^2 - F_c^2)^2/\Sigma w(F_o^2)^2]^{1/2}$

	PCN-700-BDC-CH_3	PCN-700-BDC-NH_2	PCN-700-BDC-F
CCDC	2474686	2469147	2474687
Formula	$C_{36.5}H_8O_{16}Zr_3$	$C_{36}H_{20}N_{0.5}O_{16}Zr_3$	$C_{36}H_8F_{0.5}O_{16}Zr_3$
Formula wt	976.09	989.18	979.58
Temperature (K)	110	110	110
Crystal System	tetragonal	tetragonal	tetragonal
Space Group	$P4_2/mmc$	$P4_2/mmc$	$P4_2/mmc$
a (Å)	24.2170(10)	24.418(2)	24.2739(10)
b (Å)	24.2170(10)	24.418(2)	24.2739(10)
c (Å)	14.7356(14)	14.761(2)	14.6995(13)
α (deg)	90	90	90
β (deg)	90	90	90
γ (deg)	90	90	90
Z	4	4	4
V (Å^3)	8641.9(11)	8800.9(19)	8661.3(10)
ρ_{calc} (g cm^{-3})	0.750	0.747	0.751
μ (mm^{-1})	3.196	0.380	3.198
F (000)	1900.0	1950.0	1906.0
R_{int}	0.0975	0.1661	0.1707
$^aR_1$, $^bwR_2$ ($I > 2\sigma(I)$)	0.0644, 0.1752	0.1073, 0.2615	0.0675, 0.1685
$^aR_1$, $^bwR_2$ (all data)	0.0765, 0.1908	0.1080, 0.2623	0.1066, 0.1942

$^aR_1 = \Sigma||F_o| - |F_c||/\Sigma|F_o|$, $^bwR_2 = [\Sigma w(F_o^2 - F_c^2)^2/\Sigma w(F_o^2)^2]^{1/2}$

	**PCN-700-BDC-Cl**	**PCN-700-BDC-Br**	**PCN-700-BDC-$(CH_3)_2$**
CCDC	2469150	2469148	2471422
Formula	$C_{36}H_{24}Cl_{0.5}O_{16}Zr_3$	$C_{33.6}H_{24}Br_{0.2}O_{13.1}Zr_3$	$C_{37}H_{20}O_{16}Zr_3$
Formula wt	1003.94	926.97	994.19
Temperature (K)	110	110	110
Crystal System	tetragonal	tetragonal	tetragonal
Space Group	$P4_2/mmc$	$P4_2/mmc$	$P4_2/mmc$
a (Å)	24.383(2)	24.481(5)	24.2634(13)
b (Å)	24.383(2)	24.481(5)	24.2634(13)
c (Å)	14.808(2)	14.99(2)	14.6907(11)
α (deg)	90	90	90
β (deg)	90	90	90
γ (deg)	90	90	90
Z	4	4	4
V (Å^3)	8804(2)	8983(14)	8648.5(11)
ρ_{calc} (g cm^{-3})	0.757	0.685	0.764
μ (mm^{-1})	3.278	3.129	3.196
F (000)	1986.0	1830.0	1960.0
R_{int}	0.1511	0.4352	0.2243
$^aR_1$, $^bwR_2$ ($I > 2\sigma(I)$)	0.1171, 0.3055	0.1312, 0.3049	0.0818, 0.2234
$^aR_1$, $^bwR_2$ (all data)	0.1512, 0.3446	0.1944, 0.3736	0.0989, 0.2462

$^aR_1 = \Sigma||F_o| - |F_c||/\Sigma|F_o|$, $^bwR_2 = [\Sigma w(F_o^2 - F_c^2)^2/\Sigma w(F_o^2)^2]^{1/2}$

	PCN-700-BDC-$(NH_2)_2$	PCN-700-BDC-F_2	PCN-700-BDC-Cl_2
CCDC	2469140	2480185	2480187
Formula	$C_{36}H_{20}NO_{16}Zr_3$	$C_{36}H_{24}FO_{16}Zr_3$	$C_{36}H_{24}ClO_{16}Zr_3$
Formula wt	996.19	1005.21	1021.66
Temperature (K)	110	110	110
Crystal System	tetragonal	tetragonal	tetragonal
Space Group	$P4_2/mmc$	$P4_2/mmc$	$P4_2/mmc$
a (Å)	24.4400(16)	24.2774(6)	24.2872(13)
b (Å)	24.4400(16)	24.2774(6)	24.2872(13)
c (Å)	14.404(4)	14.8919(7)	14.8125(140
α (deg)	90	90	90
β (deg)	90	90	90
γ (deg)	90	90	90
Z	4	4	4
V (Å^3)	8604(3)	8777.2(6)	8737.4(12)
ρ_{calc} (g cm^{-3})	0.769	0.761	0.777
μ (mm^{-1})	3.217	3.168	3.445
F (000)	1964.0	1988.0	2020.0
R_{int}	0.0997	0.1147	0.1254
$^aR_1$, $^bwR_2$ ($I > 2\sigma(I)$)	0.1063, 0.2865	0.1184, 0.3381	0.1228, 0.3333
$^aR_1$, $^bwR_2$ (all data)	0.1508, 0.3364	0.1434, 0.3695	0.1455, 0.3602

$^aR_1 = \Sigma||F_o| - |F_c||/\Sigma|F_o|$, $^bwR_2 = [\Sigma w(F_o^2 - F_c^2)^2/\Sigma w(F_o^2)^2]^{1/2}$

	PCN-700-BDC-Br_2
CCDC	2480186
Formula	$C_{36}H_{24}BrO_{16}Zr_3$
Formula wt	1066.12
Temperature (K)	110
Crystal System	tetragonal
Space Group	$P4_2/mmc$
a (Å)	24.500(3)
b (Å)	24.500(3)
c (Å)	15.058(3)
α (deg)	90
β (deg)	90
γ (deg)	90
Z	4
V (Å^3)	9038(3)
ρ_{calc} (g cm^{-3})	0.784
μ (mm^{-1})	3.577
F (000)	2092.0
R_{int}	0.1456
$^aR_1$, $^bwR_2$ ($I > 2\sigma(I)$)	0.1261, 0.3245
$^aR_1$, $^bwR_2$ (all data)	0.1711, 0.3707

$^aR_1 = \Sigma||F_o| - |F_c||/\Sigma|F_o|$, $^bwR_2 = [\Sigma w(F_o^2 - F_c^2)^2/\Sigma w(F_o^2)^2]^{1/2}$

Table S2. LOD values of the samples towards TFA and PFOA.

	TFA (uM)	PFOA (uM)
PCN-700-FA	176	27
PCN-700-BDC	208	20
PCN-700-NDC	256	33
PCN-700-BDC-CH_3	485	86
PCN-700-BDC-NH_2	59	8
PCN-700-BDC-F	127	110
PCN-700-BDC-Cl	211	17
PCN-700-BDC-Br	215	29
PCN-700-BDC-$(CH_3)_2$	462	48
PCN-700-BDC-$(NH_2)_2$	375	93
PCN-700-BDC-F_2	516	171
PCN-700-BDC-Cl_2	225	70
PCN-700-BDC-Br_2	243	37

Table S3. Typical luminescence sensing abilities towards PFAS in literatures.

Sample	Analyte	Response	Quenching Constant/ Association Constant	Re.
CD **1**	PFOS	Enhancement	5.21×10^{6}	12
ITHD(Zn)	PFOA	Enhancement	7.1×10^{6}	13
	TFSA		5.4×10^{6}	
	HFBA		6.0×10^{6}	
	PFBS		4.0×10^{5}	
	PFSubA		2.5×10^{6}	
Eu-FDA-CH_2OH	PFOA	Quenching	2.58×10^{3}	14
Eu-FDA			7.30×10^{2}	
Eu-FDA-NH_2	DFSA		2.70×10^{3}	
Eu-FDA			1.19×10^{3}	
Eu-FDA-1	PFOA	Quenching	2.03×10	15
Eu-FDA-2			1.58×10^{2}	
Eu-FDA-3			5.63×10^{2}	
Eu-FDA-4			1.27×10^{2}	
Eu-FDA-5			2.32×10^{2}	
Eu-FDA-6			2.34×10^{2}	
Eu-FDA-7			5.24×10^{2}	
Eu-FDA-8			1.99×10^{2}	
U-1	PFOA	Enhancement	-	16
PCN-222<br>PCN-223<br>PCN-224	PFHxA<br>PFHpA<br>PFOA<br>PFNA<br>PFDA<br>PFOS	Quenching	-	17
Au@ZIF-on-MIL<br>AgAu@ZIF-on-MIL	PFNA<br>PFOA<br>GenX<br>PFHpA<br>PFHxA<br>PFPeA<br>HFO-15<br>TFMSA	Quenching	-	18
Eu-TPDC-NH_2	PFOA	Quenching	-	19
UiO-66-$N(CH_3)_3^+$@SRB	PFOA	Enhancement	-	20

PFOS: perfluorooctane sulfonate; PFOA: perfluorooctanoic acid; TFSA: tetrafluorosuccinic acid; HFBA: heptafluorobutyric acid; PFBS: perfluorobutanesulfonic acid; PFSubA: perfluorosuberic acid; DFSA: dodecafluorosuberic acid; PFHxA: perfluorohexanoic acid; PFHpA: perfluoroheptanoic acid; PFNA: perfluorononanoic acid; PFDA: perfluorodecanoic acid; GenX: perfluoro(2-methyl-3-oxahexanoic acid); PFPeA: perfluoropentanoic acid; HFO-15: 2,2,3,3,4,4,5,5,6,6,7,7,8,8,8-heptadecafluorooctan-1-ol; TFMSA: trifluoromethanesulfonic acid; SRB: sulforhodamine B.

References

(1) Yuan, S.; Lu, W.; Chen, Y.-P.; Zhang, Q.; Liu, T.-F.; Feng, D.; Wang, X.; Qin, J.; Zhou, H.-C. Sequential linker installation: precise placement of functional groups in multivariate metal-organic frameworks. *J. Am. Chem. Soc.* **2015**, *137*, 3177-3180.

(2) Neese, F.; Wennmohs, F.; Becker, U.; Riplinger, C. The ORCA quantum chemistry program package. *J. Chem. Phys.* **2020**, *152*, 224108.

(3) Neese, F. Software update: The ORCA program system-Version 5.0. *WIREs Comput. Mol. Sci.* **2022**, *12*, e1606.

(4) Grimme, S.; Hansen, A.; Ehlert, S.; Mewes, J.-M. r2SCAN-3c: A "Swiss army knife" composite electronic-structure method. *J. Chem. Phys.* **2021**, *154*, 064103.

(5) Kruse, H.; Grimme, S. A geometrical correction for the inter- and intra-molecular basis set superposition error in Hartree-Fock and density functional theory calculations for large systems. *J. Chem. Phys.* **2012**, *136*, 154101.

(6) Caldeweyher, E.; Bannwarth, C.; Grimme, S. Extension of the D3 dispersion coefficient model. *J. Chem. Phys.* **2017**, *147*, 034112.

(7) Caldeweyher, E.; Ehlert, S.; Hansen, A.; Neugebauer, H.; Spicher, S.; Bannwarth, C.; Grimme, S. A generally applicable atomic-charge dependent London dispersion correction. *J. Chem. Phys.* **2019**, *150*, 154122.

(8) Lu, T.; Chen, Q. Independent gradient model based on Hirshfeld partition: a new method for visual study of interactions in chemical systems. *J. Comput. Chem.* **2022**, *43*, 539-555.

(9) Lu, T.; Chen, F. Multiwfn: A multifunctional wavefunction analyzer. *J. Comput. Chem.* **2012**, *33*, 580-592.

(10) Lu, T. A comprehensive electron wavefunction analysis toolbox for chemists, Multiwfn. *J. Chem. Phys.* **2024**, *161*, 082503.

(11) Humphrey, W.; Dalke, A.; Schulten, K. VMD: Visual molecular dynamics. *J. Mol. Graphics* **1996**, *14*, 33-38.

(12) Chen, Z.; Lu, Y.-L.; Wang, L.; Xu, J.; Zhang, J.; Xu, X.; Cheng, P.; Yang, S.; Shi, W. Efficient recognition and removal of persistent organic pollutants by a bifunctional

molecular material. *J. Am. Chem. Soc*. **2023**, *145*, 260-267.
(13) Han, Z.; Guo, Y.; Wang, K.-Y.; Li, W.; Huo, J.; Huang, Q.; Bakhmutov, V. I.; Yang, Y.; Liang, R.-R.; Taylor, P. R.; Shi, W.; Zhou, H.-C. Enhanced-type quantitative luminescence recognition for per- and polyfluoroalkyl substances (PFAS) by a metal-organic framework single crystal. *Angew. Chem. Int. Ed*. **2025**, e15775.
(14) Han, Z.; Wang, K.-Y.; Liang, R.-R.; Guo, Y.; Yang, Y.; Wang, M.; Mao, Y.; Huo, J.; Shi, W.; Zhou, H.-C. Modular construction of multivariate metal-organic frameworks for luminescent sensing. *J. Am. Chem. Soc.* **2025**, *147*, 3866-3873.
(15) Han, Z.; Liu, Z.; Wang, K.-Y.; Wu, Y.; Huo, J.; Yang, Y.; Liang, R.-R.; Shi, W.; Zhou, H.-C. Systematic coordination symmetry engineering in Eu-metal-organic frameworks for luminescence recognition of perfluorooctanoic acid. *CCS Chem*. **2025**, 10.31635/ccschem.025.202506773.
(16) Dalapati, R.; Hunter, M.; SK, M.; Yang, X.; Zang, L. Fluorescence turn-on detection of perfluorooctanoic acid (PFOA) by perylene diimide-based metal-organic framework. *ACS Appl. Mater. Interfaces* **2024**, *16*, 32344-32356.
(17) Chen, B.; Yang, Z.; Qu, X.; Zheng, S.; Yin, D.; Fu, H. Screening and discrimination of perfluoroalkyl substances in aqueous solution using a luminescent metal-organic framework sensor array. *ACS Appl. Mater. Interfaces* **2021**, *13*, 47706-47716.
(18) Wang, M.; Han, Y.; Sun, G.; Chen, J.; Zhao, X.; Qiu, K.; Lu, S.; Liu, D.; Wang, S.; Wang, H. Polyfluoroalkyl substance-induced nanoclusters immobilized MOF-on-MOF architecture dissociation-driven machine learning-assisted ratio fluorescence sensor array. *Anal. Chem.* **2025**, *97*, 18010-18019.
(19) Su, L.; Niu, Q.; Liu, J.; Liu, X.; Wang, Y.; Ge, Z.; Ye, Y.; Li, Z. Ratiometric luminescence detection of PFOA by amine-functionalized lanthanide metal-organic-frameworks. *Chem. Eng. J.* **2025**, *520*, 166124.
